# Concretes Modified with Insulation Wool Recovered from Recycled Heating Pipes

**DOI:** 10.3390/ma19163502

**Published:** 2026-08-18

**Authors:** Anna Starczyk-Kołbyk, Emil Kardaszuk

**Affiliations:** 1 Institute of Civil Engineering, Faculty of Civil Engineering and Geodesy, Military University of Technology, Gen. S. Kaliskiego 2, 00-908 Warsaw, Poland; 2 Research Laboratory, Faculty of Civil Engineering and Geodesy, Military University of Technology, Gen. S. Kaliskiego 2, 00-908 Warsaw, Poland; emil.kardaszuk@wat.edu.pl

**Keywords:** waste mineral wool, recycling of insulation waste, microstructure of modified concretes, thermal properties of concretes with waste additive

## Abstract

This study evaluated the potential use of waste mineral wool recovered from heating pipe insulation as a functional additive in cement concrete. The research aimed to determine the effect of this fibrous recycled material on the mechanical, physical, durability, thermal, and microstructural properties of concrete. One reference mix and three modified mixes were prepared, incorporating waste mineral wool at 12%, 16%, and 20% by cement mass. All mixes were prepared using CEM I 32.5R cement, 0/2 mm basalt aggregate, 2/8 mm granite aggregate, and a superplasticizer. After 28 days of sample curing, compressive strength, splitting tensile strength, density, water absorption, frost resistance, and thermal parameters were determined. Microstructural observations were also performed on concrete fracture surfaces. The results showed that the addition of mineral wool reduced the compressive strength from 84.9 MPa for the reference concrete to 63.8–53.3 MPa for the modified concretes. At the same time, moderate dosing improved the splitting tensile strength, reaching a maximum of 3.93 MPa with a 16% addition. The most favorable thermal effect was achieved with a 12% addition, for which the thermal conductivity coefficient decreased from 1.3461 to 1.1988 W/(m·K). The results indicate that waste mineral wool can be used in concretes with limited structural function; however, its dosage requires optimization due to increased water absorption and decreased compressive strength.

## 1. Introduction

The construction sector is currently under significant pressure to reduce natural resource consumption, minimize the environmental footprint of building materials, and efficiently manage waste generated during the demolition, renovation, and modernization of technical infrastructure. Cement concrete remains one of the most widely used structural and utility materials, so even partially incorporating recycled components into its composition can have significant technological, environmental, and economic importance. Currently, particular attention is being paid to waste that is difficult to reuse because, due to its unusual morphology, low bulk density, porosity, or operational contamination, it does not have straightforward recycling paths.

One such material is mineral wool recovered from the insulation of heating pipes dismantled during the modernization of transmission networks. These types of materials were originally used as thermal insulation, and their fibrous and porous structure gave them beneficial thermal insulation properties. After its service life, however, the material may exhibit variable fiber geometry, partial degradation, contamination, and an irregular degree of fragmentation. While these characteristics limit its direct reuse, they also indicate its potential for use as a functional additive in cement composites.

Incorporating mineral wool into concrete can affect the composite’s properties in two ways. On the one hand, the fibrous material can limit microcrack propagation and improve concrete’s resistance to indirect tension. On the other hand, excessive fiber content can reduce the mix’s workability, hinder its compaction, increase air content, promote local agglomerations of the additive, and weaken the continuity of the cement matrix. Consequently, this may lead to reduced compressive strength, increased water absorption, and a deterioration of certain durability parameters. From a material design perspective, the key is not merely to confirm that waste wool can be incorporated into concrete, but rather to determine the dosage range that achieves a favorable balance between improved thermal properties and an acceptable level of mechanical and durability properties.

Previous studies on concrete modified with waste additives indicate that the effectiveness of these solutions hinges on the type of waste, its composition, the shape of its particles or fibers, how it is prepared, its dosage, and its compatibility with the cement matrix. Even a material with beneficial thermal insulation properties is not necessarily suitable for concrete if it excessively weakens the structure or increases moisture transport. Therefore, evaluating concrete containing waste mineral wool should simultaneously cover its mechanical, physical, durability, thermal, and microstructural properties.

This study focuses on cement concrete modified with waste mineral wool recovered from district heating pipe insulation. We prepared a reference concrete and three modified concretes with wool added at 12%, 16%, and 20% by cement mass. We analyzed the relationships among the material’s composition, structure, and properties, with a particular focus on the trade-off between improved thermal performance and the deterioration of some mechanical and durability properties.

## 2. Literature Review

Mineral wool, which primarily includes rock and glass wool, is widely used as an insulating material in construction and technical infrastructure. Its utility stems from its low bulk density, porous fibrous structure, low thermal conductivity, fire resistance, and acoustic properties [1,2]. However, once its service life ends or after demolition work, this material becomes a challenging waste to manage. This is primarily due to its low bulk density, large transport volume, potential moisture content, operational contaminants, and variable chemical composition and fiber morphology [1,3].

The literature highlights that mineral wool waste is among the most challenging fractions of construction and demolition waste to recycle effectively [1]. Even though this material is a mineral product, reusing it is not straightforward because it necessitates sorting, fragmentation, chemical composition assessment, contaminant removal, and controlling the organic binder content [1,3]. Consequently, a significant amount of insulation waste still goes to landfills, which conflicts with the principles of the circular economy and policies aimed at reducing construction waste sent for disposal [1,4].

Current research into the management of waste mineral wool explores several avenues: its reuse as an insulating material; its application as a filler or fibrous additive in mortars and concretes; its use as a component in alkali-activated materials; and thermochemical processing to obtain an additive with pozzolanic properties or a binder component [1,3,4]. For cement concrete, it is particularly interesting to use the waste’s fibrous structure to simultaneously modify its thermal properties and cracking mechanism [2,5].

Previous research suggests that waste mineral wool can be incorporated into cementitious materials mainly as a partial substitute for fine aggregate, a fibrous additive, or a component that modifies the microstructure [1,2,5]. Adding mineral wool fibers to masonry and plastering mortars typically resulted in lower bulk density and reduced thermal conductivity [2]. Concurrently, an increase in capillary water absorption was noted, which researchers linked to a more open pore structure and less compact matrix [2,6].

Research on mortars with recycled mineral wool showed that fibers can enhance flexural strength and limit shrinkage, but compressive strength usually declines as the additive’s proportion increases [2]. This mechanism can be attributed to the beneficial action of fibers under tensile or flexural stress, coupled with a simultaneous weakening of the material’s compact structure when subjected to compressive loads [2,5]. Fibers can bridge microcracks and impede their spread, but too much additive results in poorer compaction, localized fiber clustering, and increased porosity [5,7].

Similar trends are observed in concretes modified with waste fibers. Research on concretes incorporating waste glass wool revealed that increasing fiber content can decrease compressive strength yet simultaneously boost splitting tensile strength and flexural strength [5]. This set of results aligns with the classic behavior of fiber composites: fibers do not always enhance compressive load-bearing capacity, but they can improve resistance to crack initiation and propagation [5,7].

Adding a fibrous additive to concrete changes how the cement composite functions. Under compressive load, the compactness of the matrix, the quality of the paste–aggregate transition zone, porosity, and the uniform distribution of components are of decisive importance [7,8]. If a fibrous additive impairs the workability of the mix or makes compaction difficult, it can result in a reduction in compressive strength [2,5]. This is particularly likely for lightweight, porous materials with irregularly shaped fibers [1,5].

The effect of fibers on tensile properties is different. In splitting and flexural tests, fibers can function as dispersed micro-reinforcement [5,7]. Their presence helps to bridge microcracks, dissipate fracture energy, and limit the rapid propagation of cracks [5,7]. This effect is beneficial only up to a certain dosage level. Once this level is exceeded, the fibers can form clusters, increase the number of voids, and weaken the continuity of the cement matrix, thereby limiting the expected strengthening effect [2,5,7].

Therefore, regarding the results of these studies, the literature provides significant justification for the observed mechanism: a decrease in compressive strength alongside an improvement in splitting tensile strength for moderate additions of waste mineral wool [2,5]. It is particularly important that the optimal mechanical effect in splitting is not necessarily achieved with the highest additive dosage, but rather with a fiber content that ensures beneficial dispersion without excessively degrading the pore structure [5,7].

One of the primary reasons for using waste mineral wool in cementitious materials is its original thermal insulation function. The low thermal conductivity of the wool stems from its porous fibrous structure, where air trapped between the fibers restricts heat transfer [1,2]. When incorporated into mortar or concrete, this material can reduce the composite’s effective thermal conductivity, particularly if it leads to a decrease in bulk density and an increase in the proportion of closed pores [2,6].

Studies on mortars incorporating recycled mineral wool have shown that increasing the proportion of this waste can lead to a reduction in thermal conductivity, and consequently an increase in the material’s thermal resistance [2]. It should be emphasized, however, that this relationship is not always linear. Thermal properties depend not only on the amount of wool but also on its fragmentation, distribution, moisture content, the quality of the mix’s compaction, and the nature of the porosity formed in the hardened composite [2,6,8].

In this context, it is crucial to differentiate between pores that are beneficial for thermal insulation and those that are detrimental to durability. While increased porosity can lower thermal conductivity, it can also simultaneously boost water absorption, moisture transport, and susceptibility to frost degradation [2,6]. Furthermore, the material’s moisture content can significantly increase its thermal conductivity, as water conducts heat much more effectively than air [6]. For this reason, evaluating concrete modified with waste mineral wool should encompass not only the lambda parameter but also water absorption, density, frost resistance, and microstructure [2,6,8].

One of the most frequently cited limitations of cementitious materials modified with fibrous waste is their increased water absorption [2,6]. Fibrous additives, characterized by low density and high porosity, can increase the proportion of air voids, compromise the matrix’s impermeability, and create localized pathways for moisture transport [2,5]. For mortars containing mineral wool, researchers observed an increase in capillary water absorption and a decrease in surface hardness [2]. This implies that enhancing thermal properties might come at the expense of environmental resistance [2,6].

From a practical standpoint, the relationship between water absorption and resistance to freeze–thaw cycles is particularly crucial. A material with increased porosity and water absorption capacity may be more prone to damage under cyclic freezing conditions [6,8]. At the same time, the presence of fibers can partially limit disintegration and crack propagation, meaning that the ultimate durability effect hinges on the balance between increased porosity and the microcrack bridging effect [5,7].

The literature also indicates that the practical utilization of waste mineral wool necessitates addressing several technological challenges. These include waste sorting, compositional stability, the potential presence of organic binders, moisture content, dusting, workplace safety, uniform fiber dispersion within the mix, and ensuring consistent workability [1,3,4]. Consequently, implementing such a solution demands not only confirmation of its material efficacy but also the development of a waste preparation procedure and quality control measures [1,4].

A review of the literature indicates that waste mineral wool is increasingly being considered as a secondary raw material for building materials, including mortars, concretes, alkali-activated composites, and insulation materials [1,3,4]. However, research is still lacking on a specific waste fraction derived from district heating pipe insulation and its impact on cement concrete, particularly when simultaneously assessing mechanical, durability, thermal, and microstructural properties.

A particularly significant gap concerns determining the trade-off between improved thermal insulation and the deterioration of compressive strength and increased water absorption [2,5,6]. While many studies analyze mechanical or thermal properties separately, a multi-criteria approach is essential for a practical assessment of a material’s suitability [1,2]. This study addresses that need by including a reference series and three dosage levels of waste mineral wool, with the material assessment based on the results of mechanical, physical, frost resistance, thermal, and microstructural tests.

Based on the available literature, it can be assumed that the analyzed material has potential as a functional additive for concretes with limited structural function or for elements where improved thermal properties and waste utilization are important [1,2,5]. However, it requires careful dosing and technological control, as an excessive amount of mineral wool can lead to a less compact structure, reduced compressive strength, and increased susceptibility to moisture transport [2,5,6].

Although various recycled fibers and other waste-derived constituents have already been widely investigated in cement-based materials, their effects depend strongly on the origin, morphology, post-service condition, and dosage of the particular waste stream.

## 3. Aim, Scope, and Novelty of Research

The aim of the research in this article was to develop and experimentally verify the concept of cement concretes modified with waste mineral wool obtained from district heating pipe insulation. The primary research objective was to quantitatively assess the impact of this fibrous recyclate on selected performance properties of concrete, specifically its mechanical strength, physical properties related to porosity and water absorption, freeze–thaw resistance, thermal parameters, and microstructure characteristics. The chosen scope of research stems from the necessity for a multi-criteria assessment of cementitious materials containing waste mineral wool, as the literature indicates that such additives can simultaneously improve certain thermal properties while deteriorating some mechanical or durability parameters [1,2,3,5,6].

The scope of the study encompassed four interconnected areas:

1. Mechanical properties, including compressive strength and splitting tensile strength after 28 days of curing.
2. Physical and durability properties, including bulk density, surface water absorption, and resistance to freeze–thaw cycles.
3. Thermal properties, including thermal conductivity, volumetric specific heat, and thermal diffusivity.
4. Microstructural observations, including digital optical microscopy of concrete fractures and assessment of the composite’s macro-, meso-, and microstructure.

The novelty of this work lies in using waste mineral wool, sourced directly from district heating pipe insulation, as a functional additive in cement concrete, and in simultaneously evaluating its impact on mechanical, durability, thermal, and microstructural properties. Unlike approaches that focus solely on improving thermal insulation or only on mechanical modification, this study analyses the actual material trade-off: reduced thermal conductivity with the simultaneous risk of decreased strength, increased porosity, and higher water absorption [2,5,6]. The results can serve as a basis for further developing solutions that align with the circular economy and sustainable design of cement composites [1,3,4].

## 4. Materials and Methods

### 4.1. Materials

Ordinary Portland cement CEM I 32.5R was used as the hydraulic binder for the concrete mixes. The aggregate skeleton consisted of crushed basalt fine aggregate with a 0/2 mm fraction and crushed granite coarse aggregate with a 2/8 mm fraction. The maximum aggregate grain size was 8 mm. Tap water with a pH of approximately 7.0 was used as mixing water. A high-range water-reducing admixture based on an aqueous solution of modified polycarboxylate ethers was used in all mixes at a constant amount of 1.50% by cement mass.

The waste material consisted of post-service mineral wool recovered from the dismantling of thermal insulation from district-heating pipes. The recovered wool was used as a fibrous waste additive after its separation from the dismantled insulation system. No additional chemical modification or thermal processing was applied within the experimental program.

The material exhibited an irregular, loose, and fibrous morphology resulting from its original insulation function, post-service condition, and the dismantling process. Digital optical microscopy confirmed the presence of individual fibers and fiber bundles with irregular geometry, including fibers embedded in the cement matrix. However, the experimental program did not include an independent determination of the chemical composition, residual organic binder content, gravimetric moisture content, or a statistically representative distribution of fiber length and diameter. The measurement presented in Figures should therefore be regarded as a representative observation of an individual visible fiber segment rather than a complete fiber-size distribution.

No quantitative assessment of possible residual organic binder or operational contamination was performed. Therefore, the investigated additive should be regarded as a heterogeneous post-service mineral-wool waste rather than a standardized commercial fiber product. This limitation should be considered when reproducing the results or applying the proposed solution to mineral-wool waste obtained from other sources.

The mineral wool was incorporated at 12%, 16%, and 20% by cement mass, corresponding to 48.6, 64.8, and 81.0 kg/m^3^ for mixtures M1, M2, and M3, respectively. The reference concrete contained no mineral wool.

### 4.2. Concrete Mix Compositions

Four series of concrete were prepared: one reference series, designated R, and three modified series, designated M1, M2, and M3 (Table 1 and Table S1). Each series maintained a constant amount of cement, water, aggregates, and chemical admixture. The variable under investigation was the quantity of waste mineral wool.

The mineral wool was introduced as an additional fibrous component and did not replace cement, mixing water, or either aggregate fraction. Therefore, the modified mixtures were not designed according to a constant-volume replacement procedure. The cement content, water content, aggregate contents, and superplasticizer dosage remained constant in all series, whereas the total mass of the batch increased with the mineral-wool dosage. The water-to-cement ratio was constant and equal to 0.486, and the superplasticizer dosage was maintained at 1.50% by cement mass.

The mineral-wool additions of 48.6, 64.8, and 81.0 kg/m^3^ corresponded to 12%, 16%, and 20% of the cement mass, respectively. An equivalent volumetric fraction of the wool was not calculated because the density of the heterogeneous post-service fibrous material in the condition used for batching was not independently determined. Consequently, the dosage values should be interpreted as mass-based additions rather than as volumetrically equivalent substitutions.

The addition of mineral wool was accompanied by a slight increase in the measured air content. The air content of the fresh mixtures increased from 4.5% for R to 4.6%, 4.7%, and 4.7% for M1, M2, and M3, respectively, while the air content of hardened concrete increased from 5.0% to 5.2–5.4%. These results indicate that the fibrous additive affected the packing and air-void structure despite the unchanged water and superplasticizer contents.

### 4.3. Preparation and Curing of Samples

The components of the concrete mixtures were weighed according to the adopted formulations and then mixed in a horizontal laboratory mixer. In the first stage, the dry components—i.e., aggregates and cement—were homogenized. Then, mixing water and an aqueous solution of the superplasticizer were added. For modified mixtures, particular attention was paid to the even distribution of the fibrous additive and the effective compaction of the mixture (Table S2).

The mixtures were placed in plastic molds and mechanically compacted on a vibrating table. Depending on the type of test, cubic samples measuring 100 × 100 × 100 mm and 150 × 150 × 150 mm were prepared. After demolding, the samples were cured for 28 days in water at 18 ± 2 °C. Tests were conducted under laboratory conditions, with an air temperature of approximately 20–22 °C and a relative humidity of about 54–58%. The method for preparing and curing the samples followed the requirements for preparing samples for strength tests [9,10].

The concrete was placed in the molds in two layers, and each layer was mechanically compacted on a vibrating table for approximately 10–15 s. The same nominal compaction procedure was adopted for all series. The increasing mineral-wool content was qualitatively observed to reduce the ease of placing and compacting the mixtures because of the irregular and low-density fibrous structure of the additive. However, the experimental program did not include separate quantitative fresh-state tests, such as slump, flow-table spread, or Vebe time, nor was the additional compaction energy required for individual mixtures measured. Therefore, the influence of mineral wool on workability and compaction difficulty is described qualitatively and should not be interpreted as a quantitatively established rheological relationship.

The mixing water and superplasticizer dosage were not adjusted to compensate for the presence of mineral wool. Thus, the study evaluates the effect of increasing the fibrous-additive content under constant water-to-cement ratio and constant admixture dosage.

### 4.4. Mechanical Tests

The compressive strength was determined on 100 × 100 × 100 mm cubic samples after 28 days of curing. The test was conducted using a calibrated hydraulic testing machine, with a stress increase rate of 0.6 ± 0.2 MPa/s. The testing procedure and the requirements for the testing machine were adopted in accordance with the relevant testing standards [11,12].

The splitting tensile strength was determined using the Brazilian method on cubic samples. The test utilized a splitting apparatus equipped with positioning elements and pads that complied with standard requirements. The stress increase rate was 0.05 ± 0.01 MPa/s. The test was performed in accordance with the procedure for determining the splitting tensile strength of concrete samples [13].

The compressive-strength results were calculated as arithmetic mean values from three specimens tested for each concrete series and are denoted as mean cube compressive strength, f_cm,cube_. No statistical characteristic strength value was determined.

### 4.5. Bulk Density and Water Absorption

The bulk density of the concrete was determined based on the mass of water-saturated samples and the volume calculated from their actual dimensions. Mass measurements were performed using a calibrated electronic balance, and sample dimensions were determined with a calibrated electronic caliper. The density determination was carried out in accordance with the procedure specific to hardened concrete [14].

Surface water absorption was determined on 150 × 150 × 150 mm cubic samples. Samples were subjected to water saturation until a constant mass was achieved and then dried at 107 ± 2 °C until a constant mass was reached. Water absorption was calculated based on the mass difference between saturated and dry samples. The procedure for assessing water absorption was based on standard requirements applicable to ordinary concrete [15].

### 4.6. Frost Resistance Testing

Frost resistance was evaluated using the F100 regime, comprising 100 freeze–thaw cycles. Samples were frozen in air at approximately −18 ± 2 °C and subsequently thawed in water at 18 ± 2 °C. For each series, both control samples and those subjected to freeze–thaw cycles were prepared. The assessment focused on residual compressive strength after F–T cycles and changes in mass resulting from surface scaling and spalling. The testing parameters were established based on concrete requirements and national guidelines concerning frost resistance [16,17].

### 4.7. Thermal Properties Testing

Thermal parameters were determined using a portable ISOMET 2114 m equipped with a surface probe. The following parameters were measured: thermal conductivity λ, expressed in W/(m·K); volumetric heat capacity c_v_, expressed in J/(m^3^·K); and thermal diffusivity a, expressed in m^2^/s. The parameter previously described as “specific heat” was therefore corrected to “volumetric heat capacity”, as the instrument output and the reported units referred to heat capacity per unit volume rather than per unit mass.

For each concrete series and each thermal parameter, ten valid measurement results were obtained (n = 10). The measurements were conducted under stable laboratory conditions at an air temperature of approximately 21–22 °C and a relative humidity of approximately 56–58%. The surface probe was applied directly to the concrete specimen surface during measurement [2,6].

All specimens had previously undergone the curing procedure described in Section 4.3. However, the gravimetric moisture content of the specimens at the time of thermal testing was not independently determined. Consequently, the thermal results refer to the specimens in their laboratory-conditioned state during testing and should not be interpreted as values obtained under a standardized oven-dry or fully water-saturated condition.

The experimental records did not include a quantitative measurement of the probe contact pressure or the thermal contact resistance between the probe and the concrete surface. The same measurement procedure and equipment configuration were applied to all concrete series to maintain comparable test conditions.

### 4.8. Microstructural Observations

A Keyence VHX-7200 digital optical microscope was used to conduct macro-, meso-, and microstructural observations. The fracture surfaces of concrete samples were examined using reflected light. The evaluation included the structure of the cement matrix, the paste–aggregate interfacial transition zone, the presence of air and capillary pores, microcracks, local agglomerations of fibrous additives, and the distribution of waste mineral wool within the concrete structure. Microstructural observations were used to complement the interpretation of mechanical and transport properties, consistent with the approach applied in the analysis of cement composites modified with fibrous and waste additives [5,7,8].

The microscopic examination was qualitative and was performed on selected fracture surfaces considered representative of the investigated concrete series. The observations were used to identify visible structural features, including mineral-wool fibers and fiber bundles, air voids, local matrix heterogeneity, microcracks, and the paste–aggregate interfacial region. No automated image segmentation, stereological analysis, quantitative pore-area determination, pore-size distribution, fiber-orientation analysis, or statistical assessment of fiber dispersion was performed. Consequently, the microscopy results are interpreted as qualitative supporting evidence and not as a quantitative characterization of the entire concrete volume.

### 4.9. Specimen Number and Statistical Analysis

For the compressive-strength, splitting-tensile-strength, bulk-density, and water-absorption tests, three specimens were tested for each concrete series (n = 3). For the internal and external frost-resistance assessments, six specimens were used for each series and each measurement stage (n = 6), including measurements performed before and after 100 freeze–thaw cycles. For each thermal parameter—thermal conductivity, volumetric heat capacity, and thermal diffusivity—ten valid measurement results were obtained for each series (n = 10). The microscopic observations were qualitative and were not subjected to statistical analysis.

**Figure 1:**
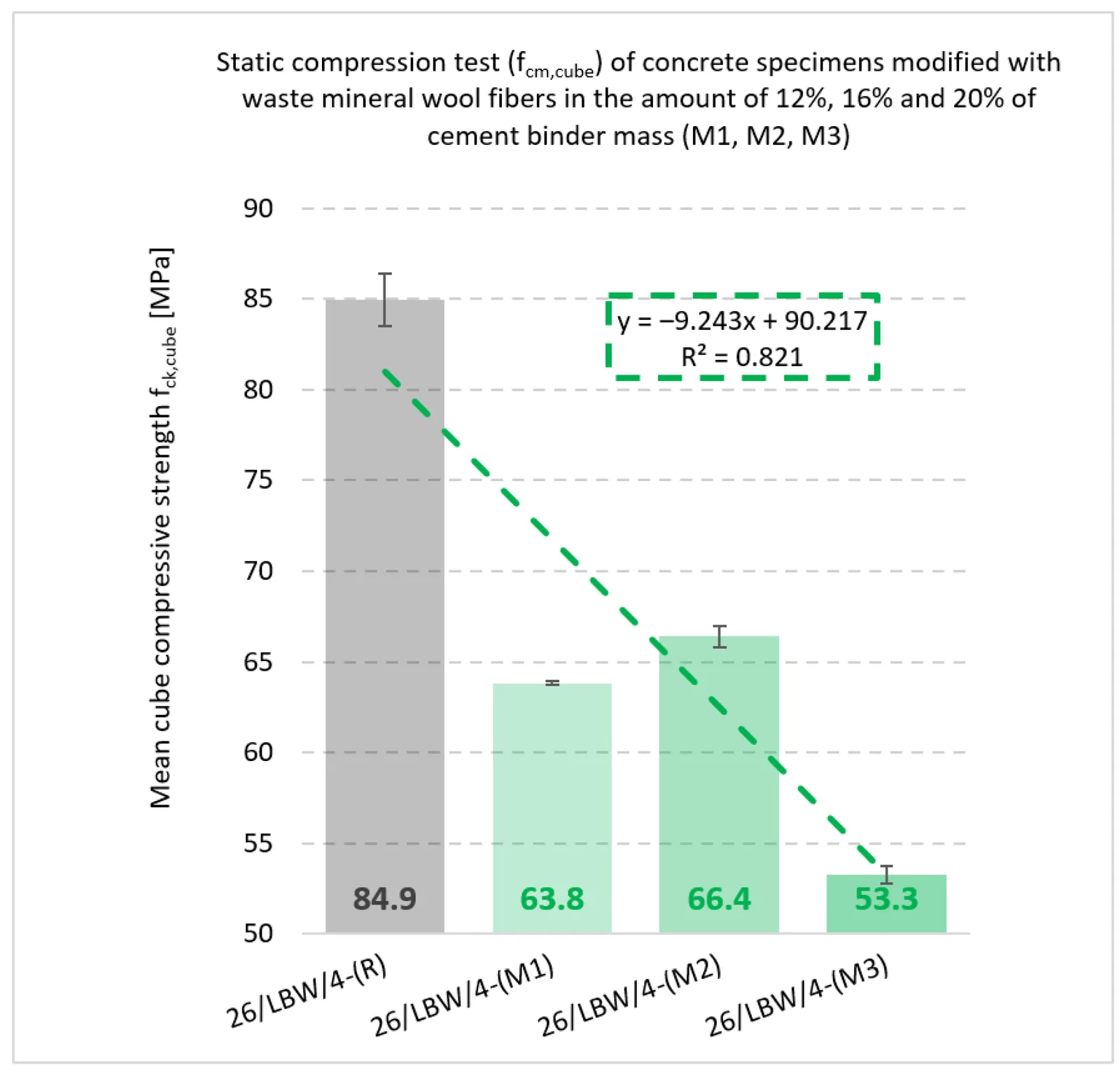
Mean cube compressive strength (f_cm,cube_) of cubic samples for the reference and material-modified series (R, M1, M2, and M3) (author’s own work).

**Figure 2:**
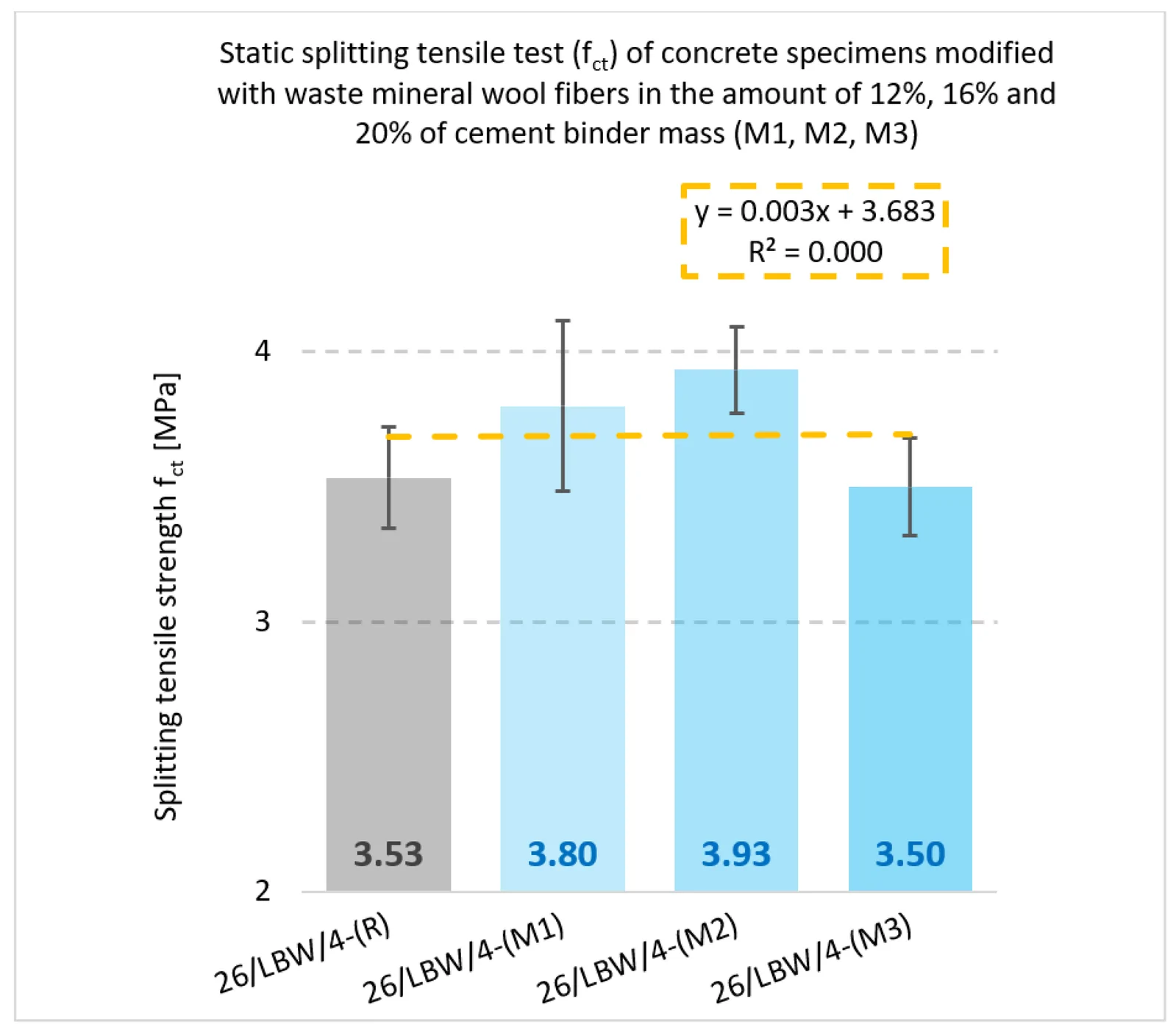
Splitting tensile strength (f_ct_) of cubic samples for the reference series and the material-modified series (R, M1, M2, and M3) (author’s own work).

**Figure 3:**
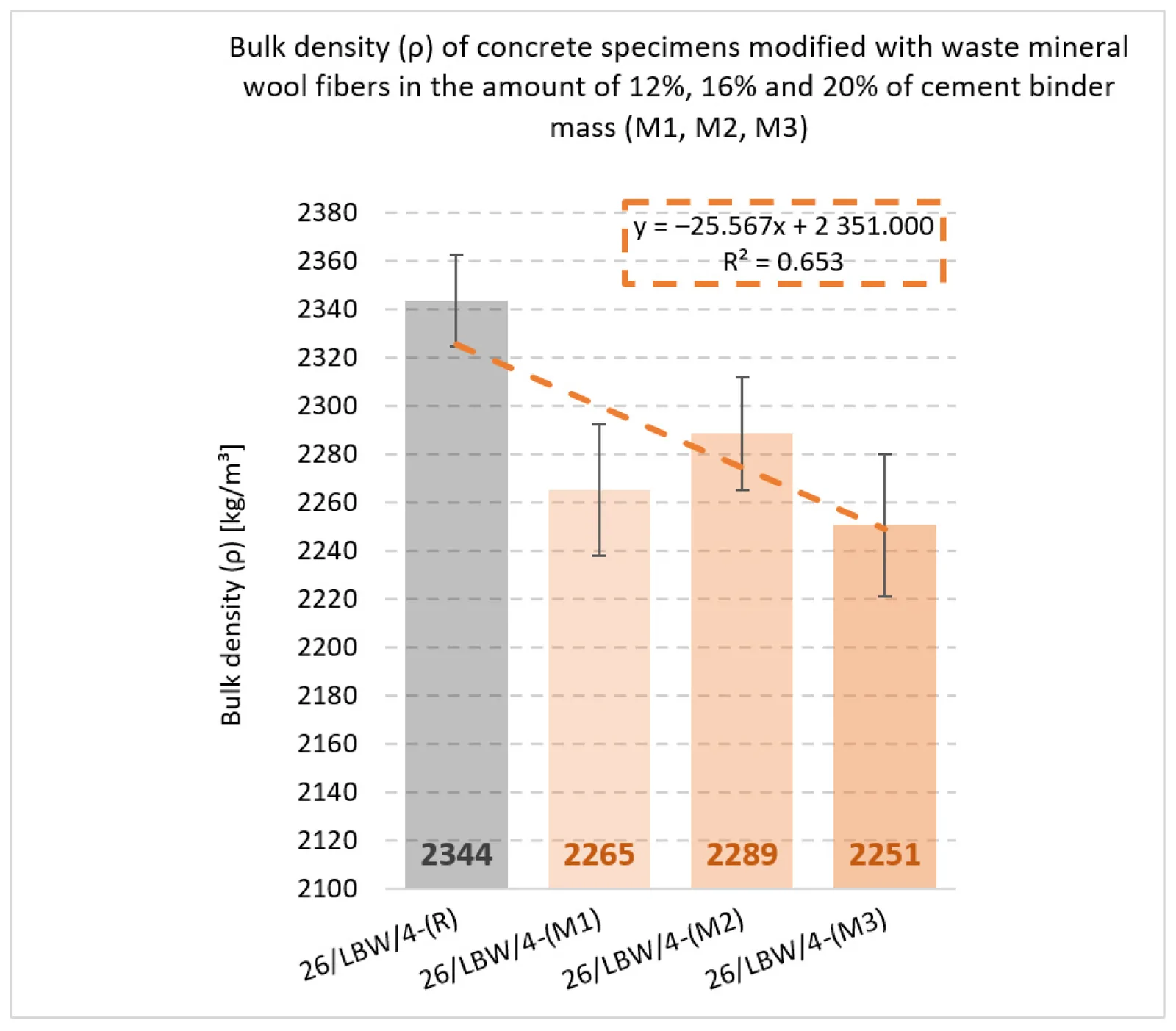
Results of bulk density (ρ) tests. Bulk density (ρ) of cubic samples for the reference series and the material-modified series (R, M1, M2, and M3) (author’s own work).

**Figure 4:**
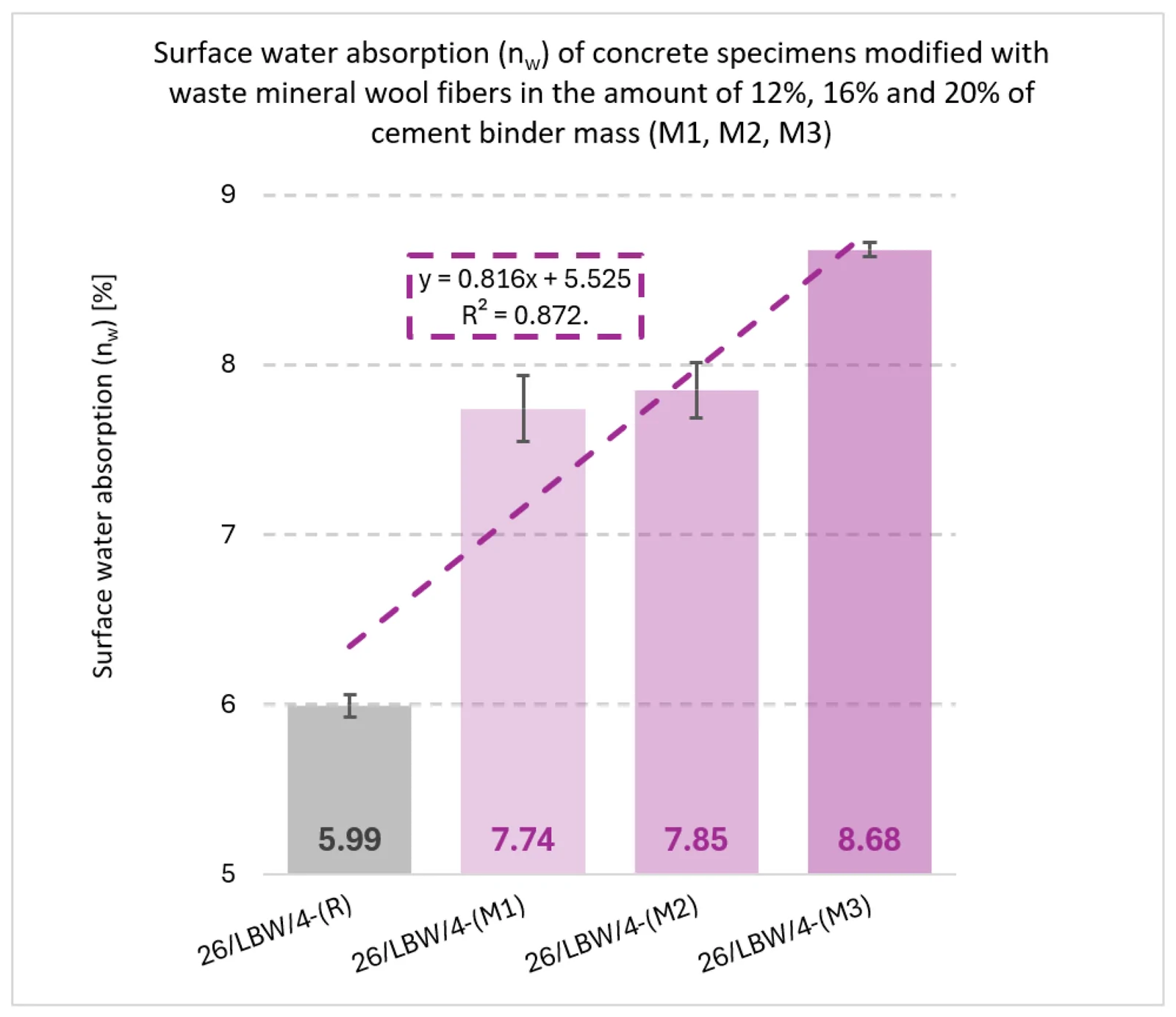
Results of surface water absorption (n_w_) tests for cubic samples from the reference series and the material-modified series (R, M1, M2, and M3) (author’s own work).

**Figure 5:**
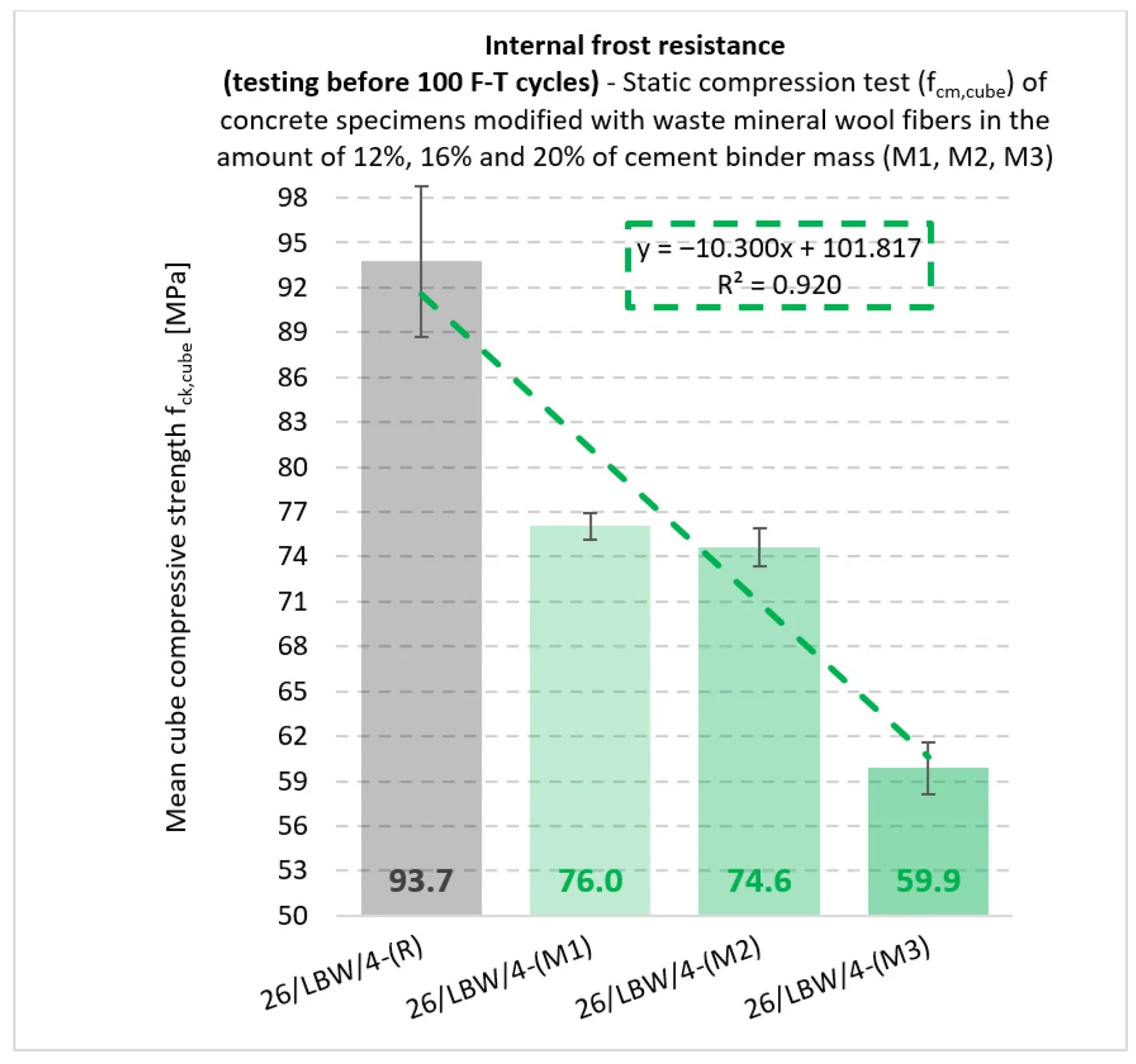
Results of tests on the reduction in mean cube compressive strength (f_cm,cube_) (internal frost resistance test of concrete before 100 F-T cycles) for cubic samples of the reference and material-modified series (R, M1, M2 and M3) (author’s own work).

**Figure 6:**
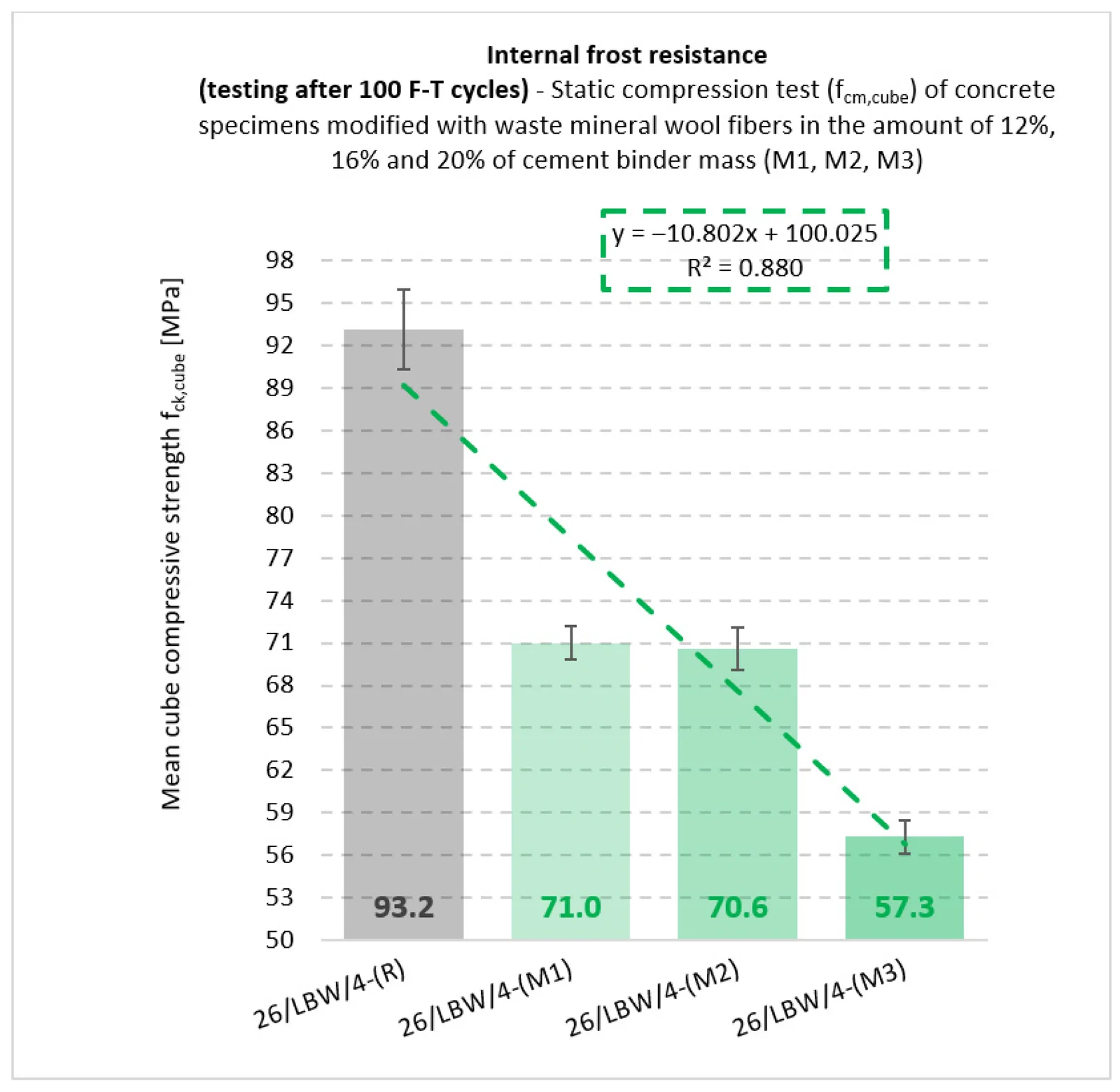
Results of tests on the reduction in mean cube compressive strength (f_cm,cube_) (internal frost resistance test of concrete after 100 F-T cycles) for cubic samples of the reference and material-modified series (R, M1, M2 and M3) (author’s own work).

**Figure 7:**
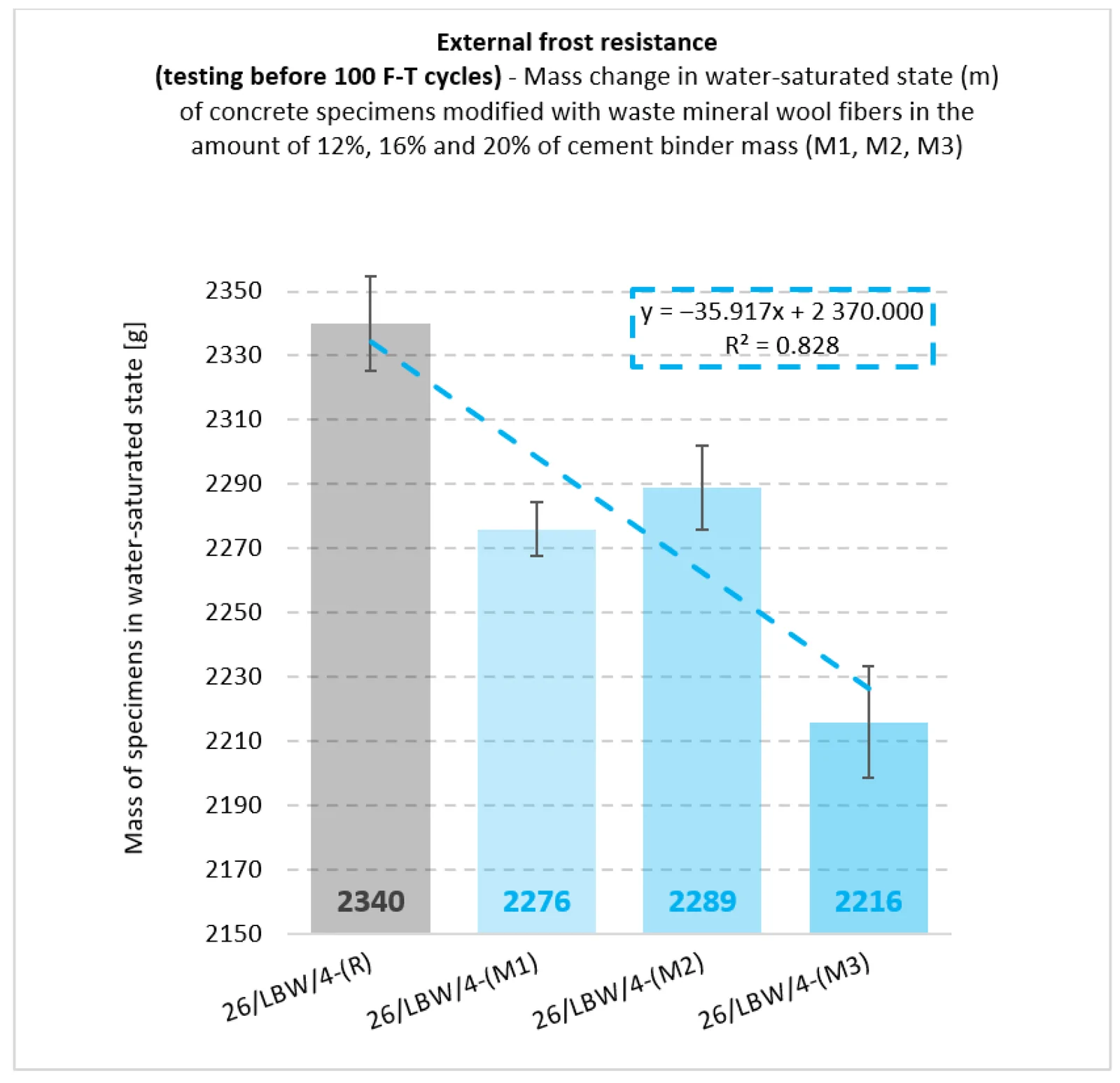
Results of mass change tests (external frost resistance test of concrete before 100 F-T cycles) on cubic samples for the reference series and the material-modified series (R, M1, M2, and M3) (author’s own work).

**Figure 8:**
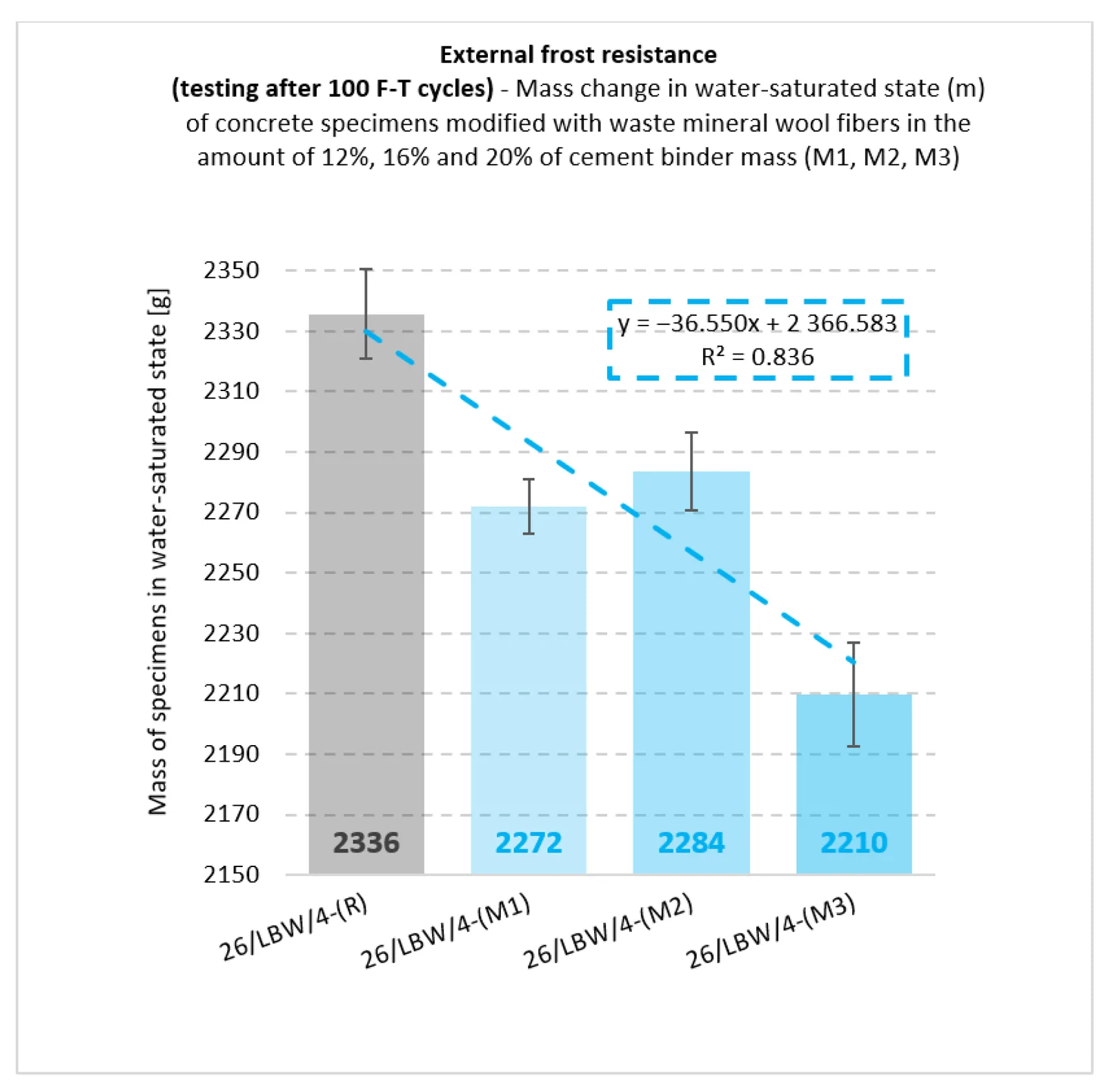
Results of mass change tests (external frost resistance test of concrete after 100 F-T cycles) on cubic samples for the reference series and the material-modified series (R, M1, M2, and M3) (author’s own work).

**Figure 9:**
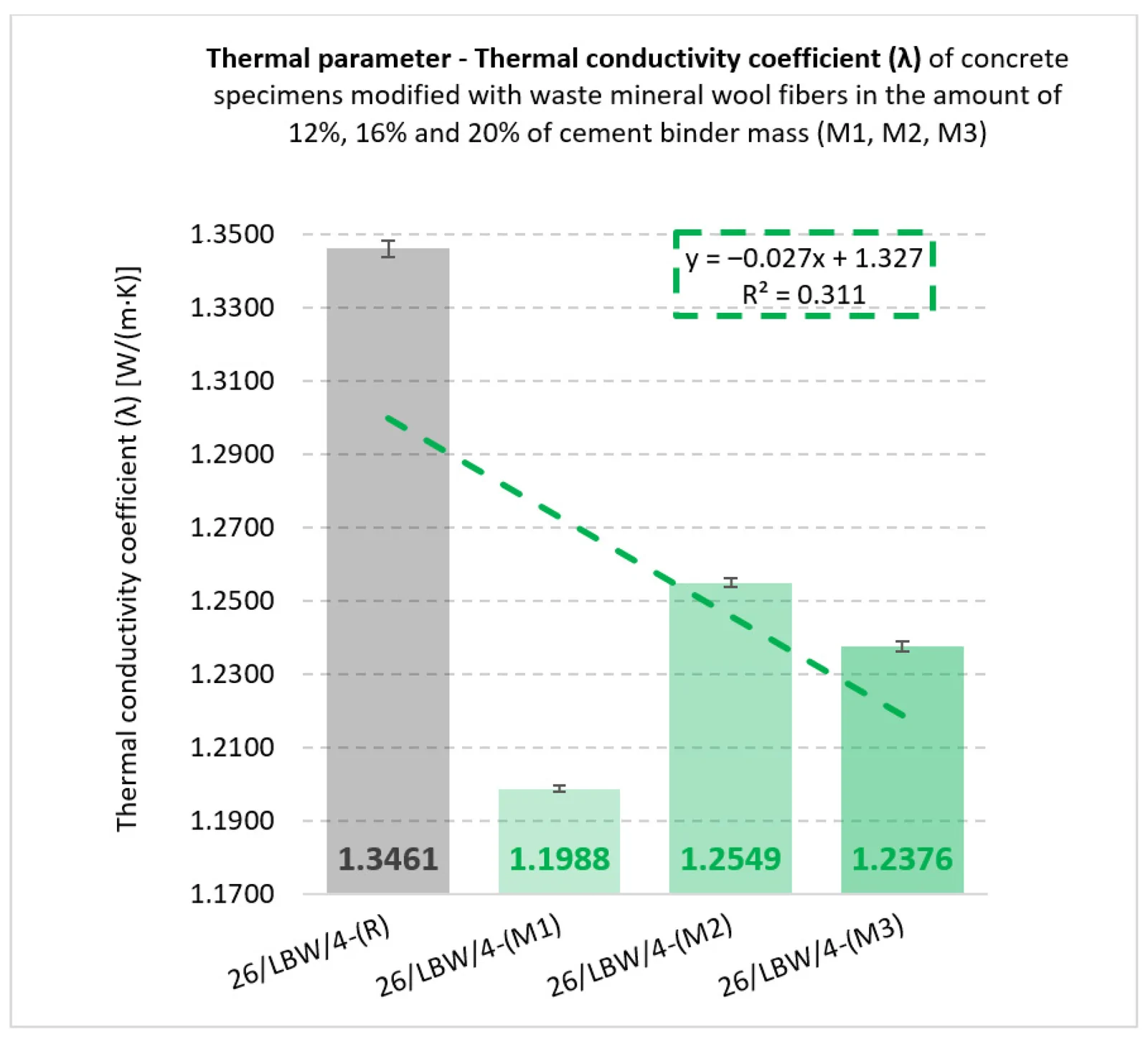
Results of thermal parameter tests. Test results for the thermal conductivity coefficient (λ) for the reference and the material-modified series (R, M1, M2, M3) (author’s own work).

**Figure 10:**
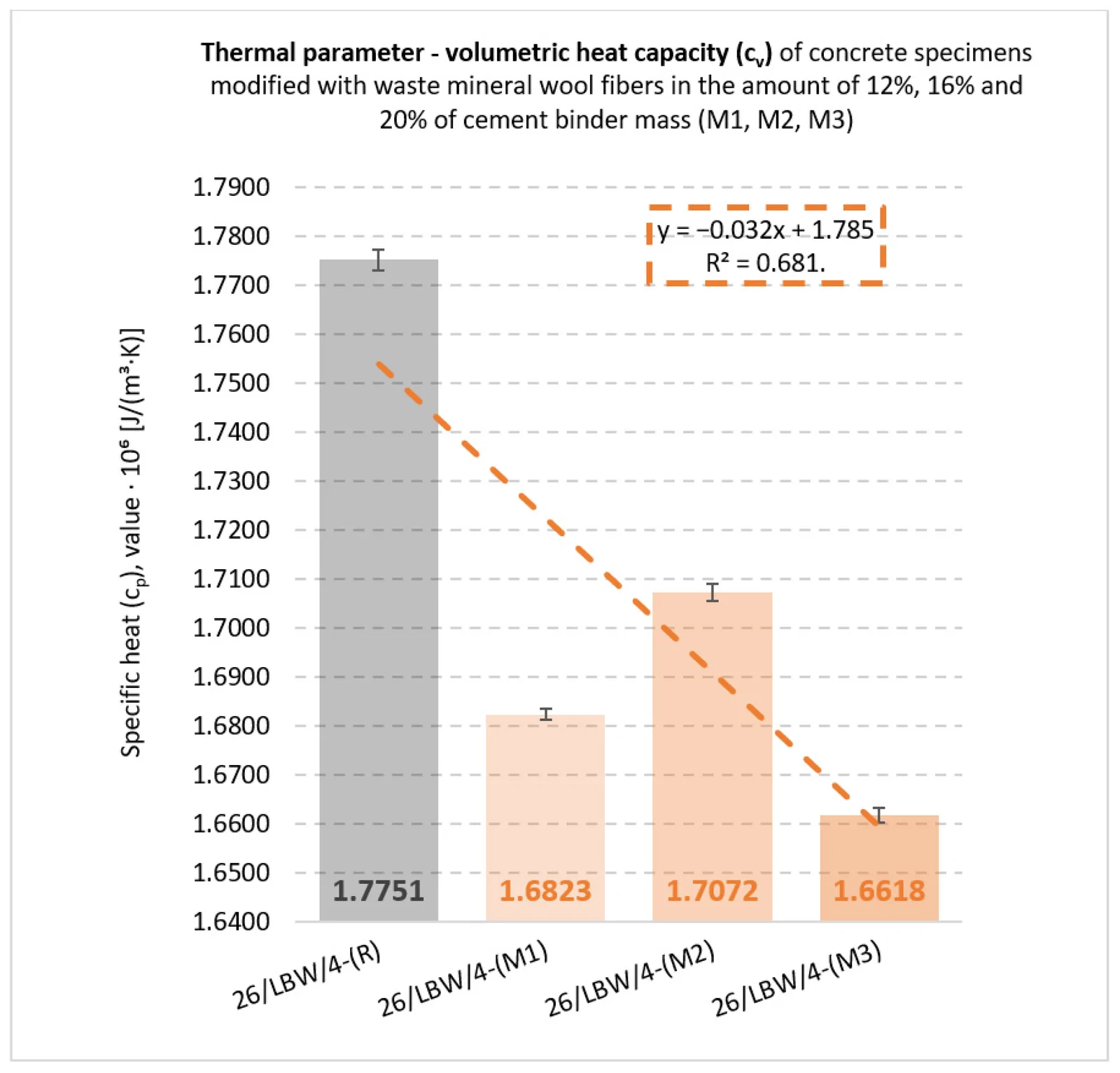
Results of thermal parameter tests. Test results for the volumetric heat capacity (c_v_) in the reference series and the material-modified series (R, M1, M2, M3) (author’s own work).

**Figure 11:**
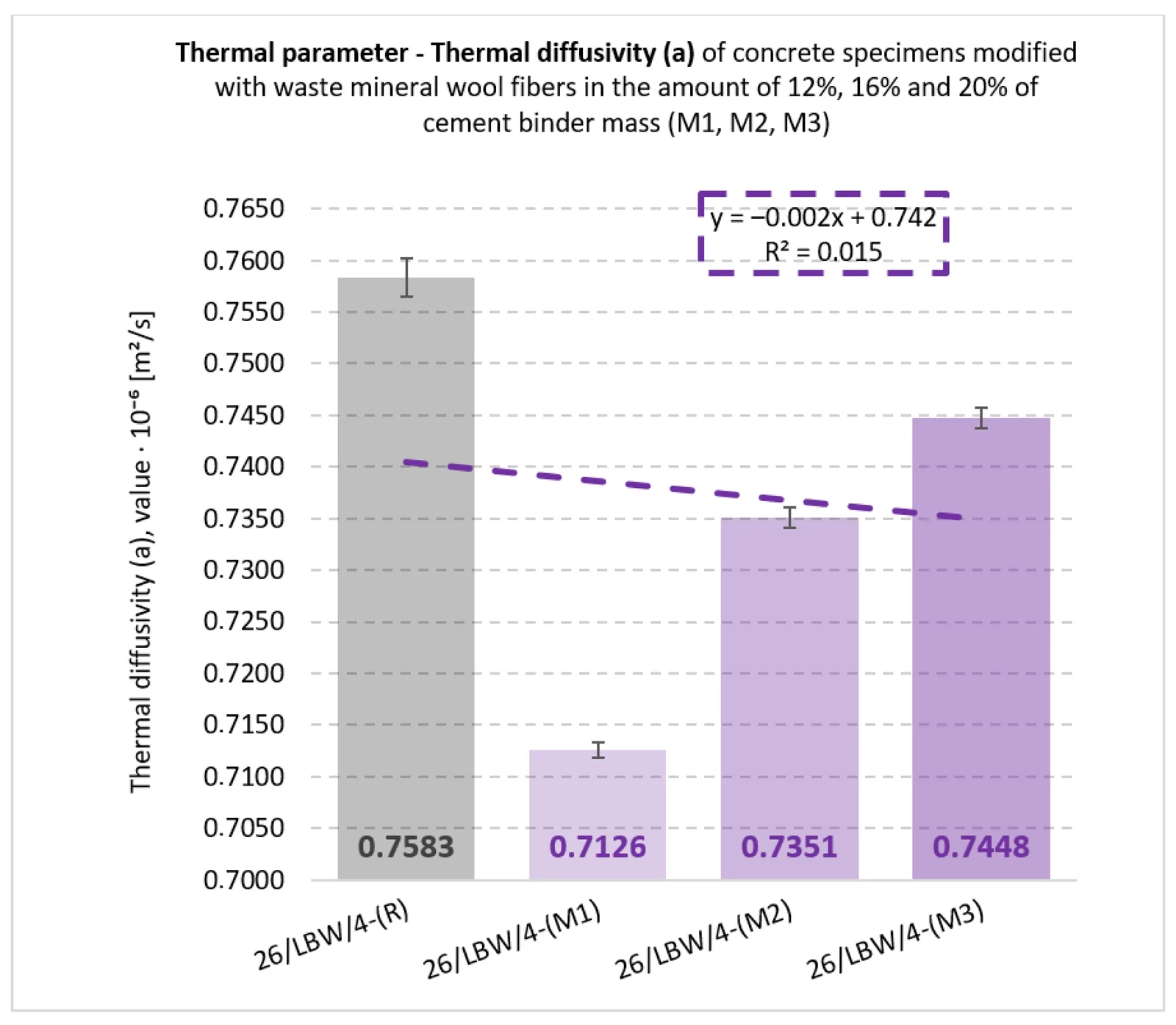
Results of thermal parameter tests. Results of tests for thermal diffusivity of the material (a) for the reference series and the modified series (R, M1, M2, M3) (author’s own work).

The numerical results are presented as arithmetic mean values. Error bars in Figures 1–11 represent one standard deviation (±SD) calculated from the results obtained for the corresponding series. Descriptive statistics, including the arithmetic mean, median, minimum, maximum, standard deviation, coefficient of variation, and 95% confidence interval, were calculated for the individual datasets. The linear trend line was used descriptively to illustrate the overall direction of change across the four mixture series. The trend-line equations and coefficients of determination (R^2^) were not treated as inferential statistical tests or predictive models. Detailed descriptive-statistics tables are provided in the Supplementary Materials.

## 5. Test Results

### 5.1. Compressive Strength

Figure 1 presents the mean cube compressive strength (f_cm,cube_) determined after 28 days of curing. The values represent arithmetic means calculated from three specimens tested for each concrete series (n = 3), and the error bars represent one standard deviation (±SD). The findings indicate a decrease in mean cube compressive strength values with an increasing proportion of waste mineral wool fibers. The M3 series showed the most significant decrease in strength (f_cm,cube_), indicating that the highest modification level had the most detrimental impact on the cement concrete’s mechanical properties. Figure 1 illustrates the test results for mean cube compressive strength (f_cm,cube_).

An analysis of the chart’s results revealed that incorporating waste mineral wool fibers at 12%, 16%, and 20% by mass of the cement binder reduced the mean cube compressive strength (f_cm,cube_) values compared to the reference series. The base sample yielded a value of 84.9 MPa, while the modified series recorded 63.8 MPa, 66.4 MPa, and 53.3 MPa, respectively. This represents a strength reduction of 24.9%, 21.8%, and 37.2% when compared to the reference sample. The trend line’s behavior, described by the equation (y = −9.243x + 90.217), and the coefficient of determination (R^2^ = 0.821) confirm a clear downward trend. The results suggest that adding waste mineral fibers disrupts the homogeneity of the cement composite’s microstructure, which led to a decrease in its mean cube compressive strength (Table S3).

### 5.2. Splitting Tensile Strength

A bar chart was prepared for the final test results concerning the splitting tensile strength (f_ct_) of cement concrete samples. Detailed test results are presented in Figure 2.

The chart presents the results of the static splitting tensile test (f_ct_) for reference concrete and concretes modified with waste mineral wool fibers at 12%, 16%, and 20% by mass of the cement binder. A value of 3.53 MPa was obtained for the reference sample; for the modified series, the values were 3.80 MPa, 3.93 MPa, and 3.50 MPa, respectively. Introducing the fibrous additive in amounts corresponding to series M1 and M2 positively affected the splitting tensile strength, increasing the tested parameter by approximately 7.6% and 11.3%, respectively, compared to the reference series.

The highest (f_ct_) value was obtained for series M2, indicating an optimal dosage level for the additive. For series M3, the (f_ct_) value decreased to a level similar to the reference sample, indicating a reduction in the homogeneity of the cement composite’s microstructure due to an excessive fiber content. The linear trend equation (y = 0.003x + 3.683) and the coefficient of determination (R^2^ = 0.000) indicate that the relationship between fiber content and splitting strength is not linear, suggesting non-linear material behavior. The results suggest that waste mineral wool fibers can positively influence concrete properties regarding indirect tensile strength, but only with a properly selected dosage level.

The maximum (f_ct_) value was recorded for series M2, suggesting an optimal fiber dosage range exists for the tensile stress transfer mechanism. It can be assumed that this level of modification achieved the most favorable balance between the fibers’ microcrack bridging effect and the degree of disruption to the cement matrix continuity (microstructural material imperfections).

The increased strength observed in the M1 and M2 series helped to limit crack propagation during splitting. The decrease in values for the M3 series suggests that once the waste fiber content exceeds 20%, the reinforcing effect diminishes due to the material’s structural heterogeneity. Linear trend analysis confirms that there is no straightforward linear relationship between the modification level and the splitting tensile strength. The regression equation (y = 0.003x + 3.683) and the coefficient of determination (R^2^ = 0.000) both indicate that the linear model does not adequately describe the phenomenon under investigation. The influence of waste mineral fiber content on strength (f_ct_) is not monotonic; it is purely optimizing and beneficial up to a 16% waste fiber dosage level (Table S4).

### 5.3. Bulk Density (ρ) of Concrete Samples

A bar chart was prepared to present the final test results for the bulk density of cement concrete samples. Detailed test results are presented in Figure 3.

The chart presents the bulk density test results for reference concrete samples and those modified with waste mineral wool fibers at 12%, 16%, and 20% by mass of the binder cement. The highest bulk density value was obtained for the reference series R, i.e., 2344 kg/m^3^. Lower values were recorded for the modified series, specifically 2265 kg/m^3^ for M1; 2289 kg/m^3^ for M2; and 2251 kg/m^3^ for M3. Compared to the reference sample R, decreases of 79 kg/m^3^, 55 kg/m^3^, and 93 kg/m^3^ were observed, respectively, corresponding to reductions of 3.37%, 2.35%, and 3.97%. The results obtained indicate that using waste mineral wool fibers leads to a reduction in the bulk density of concrete (ρ). This is also confirmed by the trend line described by the equation (y = −25.567x + 2351.000) and the coefficient of determination (R^2^ = 0.653), which indicates a decreasing dependency. The decrease in density can be linked to impaired mechanical compaction of the concrete mixture, increased porosity (negative air macropores), and a disruption in the homogeneity of the cement composite’s microstructure (imperfections).

Studies on bulk density (ρ) showed that incorporating waste mineral wool fibers in amounts of 12%, 16%, and 20% by mass of cement binder reduces the bulk density compared to reference concrete R. The reference series R yielded a value of 2344 kg/m^3^, while the modified series recorded 2265 kg/m^3^ (M1 series), 2289 kg/m^3^ (M2 series), and 2251 kg/m^3^ (M3 series). This corresponds to a reduction in bulk density (ρ) of 3.37%, 2.35%, and 3.97% relative to the reference sample R. Despite a slight increase in value for the M2 series compared to M1, the overall trend remains decreasing, as confirmed by the linear regression equation (y = −25.567x + 2351.000) and the coefficient of determination.

The results suggest that the presence of waste mineral fibers impairs the compactness of the composite’s microstructure due to increased material macroporosity, hindered mixture compaction, and a loss of microstructural homogeneity. From the perspective of concrete technology, the observed effect should be considered unfavorable at the highest level of material modification (20% by mass of cement binder) with the addition of waste mineral fibers. Bulk density (ρ) test results indicate that using waste mineral wool fibers in amounts of 12%, 16%, and 20% by mass of cement binder reduces the bulk density of cement concrete. The trend of changes is not perfectly monotonic because the M2 series achieved a higher density than M1 (the overall direction of changes is decreasing). This is confirmed by the linear regression equation (y = −25.567x + 2351.000) and the high coefficient of determination (R^2^ = 0.653), which indicates a strong negative correlation between the level of modification and the bulk density (Table S5).

### 5.4. Surface Water Absorption (n_w_) of Concrete Samples

A bar chart was prepared to present the final test results for the surface water absorption (n_w_) of cement concrete samples. Detailed test results are presented in Figure 4.

The surface water absorption (n_w_) test results showed that incorporating waste mineral wool fibers at 12%, 16%, and 20% by mass of the cement binder significantly increased surface water absorption (n_w_) compared to the reference concrete R. The reference series R achieved a value of 5.99%, whereas the modified series recorded 7.74% (M1 series), 7.85% (M2 series), and 8.68% (M3 series). This corresponds to an increase in absorption of 29.2%, 31.1%, and 44.9% relative to the reference sample R. Linear regression analysis confirmed this observed trend, yielding the equation (y = 0.816x + 5.525) and a coefficient of determination (R^2^ = 0.872), which points to a strong positive correlation between the extent of material modification using waste mineral wool fibers and surface absorption.

The results obtained suggest that the presence of waste mineral fibers reduces the composite’s structural integrity, primarily due to increased open porosity (surface macropores and capillary pores), hindered mechanical compaction of the concrete mix, and weakened microstructural homogeneity (material imperfections in the microstructure of the concrete). The high coefficient of determination (R^2^) indicates a good fit of the linear model to the experimental data and confirms a significant positive correlation between the modification level and surface water absorption (n_w_). From a material analysis perspective, this indicates that an increased proportion of waste mineral fibers correlates with greater water-accessible open porosity and a deterioration in the continuity of the matrix (Table S6).

### 5.5. External and Internal Frost Resistance of Concrete Samples

The final laboratory test results for the surface and internal frost resistance of concrete are presented in bar charts, complete with standard error bars and trend line(s) that include the linear equation(s) for the analyzed lines. Detailed results of frost resistance tests before 100 F-T cycles, showing the reduction in mean cube compressive strength (f_cm,cube_), are presented in Figure 5.

The presented test results indicate that the mean cube compressive strength of concrete samples, in terms of internal frost resistance, decreased as the proportion of waste mineral fibers increased. The reference series R achieved a value of 93.7 MPa, while the modified series yielded 76.0 MPa for M1, 74.6 MPa for M2, and 59.9 MPa for M3. Compared to the reference sample R, this represents a decrease in the tested parameter (f_cm,cube_ during internal frost resistance testing) of 18.9%, 20.4%, and 36.1%. The linear trend equation (y = −10.300x + 101.817) and a high coefficient of determination (R^2^ = 0.920) confirm a strong inverse relationship between the level of modification and the mean cube compressive strength. The results suggest that an increase in the content of waste mineral fibers negatively impacts the structure of the cement composite, leading to a reduction in its mechanical resistance within the analyzed scope.

Detailed results of frost resistance tests after 100 F-T cycles, showing the reduction in mean cube compressive strength (f_cm,cube_), are presented in Figure 6.

The chart presents the mean cube compressive strength results for concrete samples after 100 freeze–thaw cycles, as part of the internal frost resistance assessment. The reference series R achieved a value of 93.2 MPa; for the modified series, the values were 71.0 MPa for M1, 70.6 MPa for M2, and 57.3 MPa for M3. Compared to the reference sample, this indicates a decrease in the tested parameter by 22.2 MPa (23.8%), 22.6 MPa (24.2%), and 35.9 MPa (38.5%). The average value for all analyzed series was 73.03 MPa; the median was 70.8 MPa; the range 35.9 MPa. The linear trend equation (y = −10.802x + 100.025) and the coefficient of determination (R^2^ = 0.880) indicate a clear decreasing relationship between the modification level and compressive strength after freeze–thaw cycles. The findings suggest that an increase in the content of waste mineral fibers adversely affects the mechanical resistance of concrete under exposure conditions (Table S7).

Detailed results of frost resistance tests before 100 F-T cycles, regarding changes in the mass of water-saturated samples (m), are presented in Figure 7.

The chart shows the mass of water-saturated concrete samples before undergoing 100 freeze–thaw cycles as part of the external frost resistance test. For the reference series R, a value of 2340 g was obtained, while for the modified series, the values were 2276 g for series M1; 2289 g for series M2; and 2216 g for series M3. Compared to the reference sample R, this represents a mass decrease of 64 g (2.74%); 51 g (2.18%); and 124 g (5.30%). The average value for all analyzed series was 2280.25 g; the median was 2282.5 g; the range was 124 g. The linear trend equation (y = −35.917x + 2370.000) and the coefficient of determination (R^2^ = 0.828) indicate a decreasing relationship between the level of material modification and the mass of saturated samples (Table S8).

Detailed results of frost resistance tests after 100 F-T cycles, regarding changes in the mass of water-saturated samples (m), are presented in Figure 8.

The chart shows the mass of water-saturated concrete samples after 100 freeze–thaw cycles in the external frost resistance test. For the reference series R, a value of 2336 g was obtained, while for the modified series, the values were 2272 g for series M1, 2284 g for series M2, and 2210 g for series M3. Compared to the reference sample, this indicates a mass decrease of 64 g (2.74%), 52 g (2.23%), and 126 g (5.39%). The average value for all analyzed series was 2275.5 g; the median was 2278 g, and the range was 126 g. The linear trend equation (y = −36.550x + 2366.583) and the coefficient of determination (R^2^ = 0.836) indicate a clear decreasing relationship between the modification level and the mass of samples after freeze–thaw cycles (Tables S9 and S10).

### 5.6. Thermal Conductivity Coefficient

A series of tests was conducted to determine the values of thermal parameters, namely: (λ)—the material’s thermal conductivity coefficient [W/(m·K)]; (c_v_)—volumetric heat capacity [J/(m^3^·K), value·10^6^]; and (a)—the material’s thermal diffusivity [m^2^/s, value·10^−6^]. A column chart was developed, featuring trend lines and standard error bars referenced to the population standard deviation, and linear equations were determined for the indication trend lines (trend lines described by a linear function).

The results of the thermal parameter tests, specifically the thermal conductivity coefficient (λ) for the reference and material-modified series, are presented in Figure 9.

The chart presents the thermal conductivity coefficient (λ) values for reference concrete samples and those modified with waste mineral wool fibers at 12%, 16%, and 20% of the mass of the cement binder. The reference series R yielded a value of 1.3461 W/(m·K), while the modified series showed values of 1.1988 W/(m·K) for M1, 1.2549 W/(m·K) for M2, and 1.2376 W/(m·K) for M3, respectively. Compared to the reference sample, this represents a decrease in the thermal conductivity coefficient of 0.1473 W/(m·K) (10.94%) for series M1; 0.0912 W/(m·K) (6.77%) for series M2; and 0.1085 W/(m·K) (8.06%) for series M3. The lowest value for parameter (λ), and thus the most favorable thermal insulation properties, was obtained for series M1. The linear trend equation (y = −0.027x + 1.327) indicates a general decreasing tendency; however, the low coefficient of determination (R^2^ = 0.311) suggests a poor fit of the linear model to the data. The findings suggest that incorporating waste mineral wool fibers can enhance concrete’s thermal insulation. However, this improvement does not exhibit a clear linear correlation with the amount of additive used (Table S11).

Figure 10 presents the results of thermal parameter tests, specifically the volumetric heat capacity (c_v_), for both the reference series and the modified materials.

The chart displays the volumetric heat capacity (c_v_) values for reference concrete samples and those modified with waste mineral wool fibers in amounts of 12%, 16%, and 20% of the cement binder. For the reference series R, a value of 1.7751·10^6^ J/(m^3^·K) was obtained, while for the modified series, respectively, 1.6823·10^6^ J/(m^3^·K) for series M1; 1.7072·10^6^ J/(m^3^·K) for series M2; and 1.6618·10^6^ J/(m^3^·K) for series M3. Compared to the reference sample, this means a decrease in the tested parameter by 5.23% for series M1; 3.83% for series M2; and 6.38% for series M3. The average value for all analyzed series was 1.7066·10^6^ J/(m^3^·K); the median was 1.6948·10^6^ J/(m^3^·K); the range was equal to 0.1133·10^6^ J/(m^3^·K). The linear trend equation (y = −0.032x + 1.785) and the determination coefficient (R^2^ = 0.681) indicate a general decreasing trend. The obtained results suggest that the use of waste mineral wool fibers reduces the thermal capacity of concrete, although the course of changes is not linear (Table S12).

The results of the thermal parameter tests—i.e., the thermal diffusivity of the material (a) for the reference series and the modified series—are presented in Figure 11.

The chart displays the thermal diffusivity (a) values for reference concrete samples and those modified with waste mineral wool fibers, incorporated at 12%, 16%, and 20% of the cement binder mass. The reference series R yielded a value of 0.7583·10^−6^ m^2^/s. For the modified series, the values were 0.7126·10^−6^ m^2^/s for series M1; 0.7351·10^−6^ m^2^/s for series M2; and 0.7448·10^−6^ m^2^/s for series M3. Compared to the reference sample, this represents a decrease in the measured parameter of 6.03% for series M1, 3.06% for series M2, and 1.78% for series M3. The average value for all analyzed series was 0.7377·10^−6^ m^2^/s, the median was 0.7400·10^−6^ m^2^/s, and the range was 0.0457 10^−6^ m^2^/s. The linear trend equation (y = −0.002x + 0.7421), with a coefficient of determination (R^2^ = 0.015), indicates a very weak linear relationship between the modification level and thermal diffusivity (a). The results suggest that using waste mineral wool fibers reduces the thermal diffusivity of concrete compared to the reference sample; however, this effect does not show a regular trend with increasing additive content (Table S13).

The moisture content of the specimens was not determined gravimetrically at the time of testing. Therefore, the reported thermal parameters characterize the specimens under the laboratory-conditioned state used in the experimental program and should not be directly compared with the literature values obtained under explicitly oven-dry or fully saturated conditions.

### 5.7. Microstructural Observations

Qualitative microscopic observations showed visible differences between the selected fracture surfaces of the reference and mineral-wool-modified concretes. The representative images of the modified series revealed individual mineral-wool fibers and fiber bundles embedded in the cement matrix, as well as local air voids and regions of matrix heterogeneity. Selected images, particularly those obtained for the higher mineral-wool dosages, also showed visible macropores adjacent to the cement matrix and fibrous component.

These observations are consistent with, but do not quantitatively prove, the interpretation that the fibrous additive affected mixture compactness and the pore structure of the hardened concrete. The observed features may contribute to the lower bulk density, increased water absorption, and reduced compressive strength of the modified concretes. Similarly, the presence of fibers crossing or located near fracture paths provides qualitative support for a possible microcrack-bridging mechanism in series M1 and M2. However, the frequency of such features, pore volume fraction, fiber orientation, and degree of fiber dispersion were not quantified.

Figure 12, Figure 13, Figure 14, Figure 15, Figure 16, Figure 17, Figure 18, Figure 19, Figure 20, Figure 21, Figure 22, Figure 23, Figure 24 and Figure 25 present selected representative digital optical microscopy images of fracture surfaces from the reference and mineral-wool-modified concrete series. The images were selected to illustrate the principal qualitative features discussed in the text and do not constitute a quantitative image-analysis dataset.

In series M1 and M2, no clear signs of strong aggregate detachment from the cement paste were observed. The observed cracks in granite aggregate grains may indicate good adhesion between the aggregate and the cement matrix. In series M3, the excessive proportion of the additive likely disrupted the structural continuity and reduced the composite’s mechanical efficiency.

## 6. Discussion

The obtained results indicate a clear relationship between the amount of waste mineral wool and the performance properties of cement concrete. It is crucial to note that mineral wool does not behave like a typical mineral filler. Due to its fibrous and porous structure, it simultaneously affects the workability of the mix, compaction efficiency, porosity, moisture transport, heat conduction, and the cracking mechanism [1,2,5].

The decrease in compressive strength was an expected phenomenon. Introducing low-density, fibrous material into a dense cement matrix can lead to an increase in void volume and a deterioration of structural homogeneity. Additionally, the presence of fibers can make it harder to compact the mixture, particularly with higher dosages. The M3 series clearly confirms this, showing the most significant reduction in compressive strength. Similar trends have been previously reported for both mortars containing recycled mineral wool and concretes with added waste glass wool [2,5].

The splitting tensile strength results, however, showed a different trend. The increased values of this parameter for the M1 and M2 series suggest that a moderate amount of fibrous additive can positively influence the cracking mechanism. Mineral wool can act as dispersed micro-reinforcement, limiting the development of microcracks and partially bridging fracture zones. This effect has been described in studies on cementitious materials modified with mineral fibers and waste glass fibers [5,7]. The best result obtained for M2 indicates that a 16% cement mass content may be close to the optimum for indirect tensile strength.

The thermal properties confirmed the functional potential of waste mineral wool. The M1 series achieved the lowest thermal conductivity coefficient, not the series with the highest additive content. This is an important observation because it shows that simply increasing the amount of insulating waste does not automatically lead to a proportional improvement in the concrete’s thermal insulation. At higher dosages, workability, homogeneity, and pore structure may deteriorate, thereby limiting the expected thermal effect. Similar conclusions were presented in studies on mortars and cement composites with added mineral wool waste, where the thermal effect depended not only on the amount of additive but also on its distribution and influence on the material’s porosity [2,6].

From a practical standpoint, the M1 series can be considered the most beneficial for reducing thermal conductivity, while the M2 series demonstrates the best effect in terms of splitting tensile strength. The M3 series can be considered over-modified under the adopted formulation, as it led to the largest reduction in compressive strength without simultaneously providing the best thermal performance. The selection of the optimal mix therefore depends on the material’s intended function. For elements requiring enhanced thermal insulation but having a limited structural role, the M1 series may be more advantageous. For applications where scratch resistance and indirect tensile strength are more critical, the M2 series can be considered.

The formation of the locally visible air macropores may be associated with several interacting mechanisms. The irregular, low-density fibrous morphology of the post-service mineral wool may hinder the rearrangement of aggregate particles and the release of entrapped air during vibration. Fiber bundles may also create locally less compact regions and increase the surface area that must be coated by cement paste. Because the water and superplasticizer contents were kept constant while mineral wool was introduced as an additional component, the available paste may have been less effective in filling local spaces around fibers and aggregate particles. These effects could promote incomplete local compaction and the retention of air voids, which may contribute to reduced compressive strength, increased water absorption, and lower freeze–thaw resistance. However, these mechanisms should be regarded as plausible interpretations rather than directly verified causes, because no quantitative pore analysis, fresh-state rheological measurements, or compaction-energy assessment was performed.

It should be emphasized, however, that the increased water absorption limits the direct use of such concretes in environments exposed to moisture and frost. In practice, this material should primarily be considered for sheltered elements, non-load-bearing layers, prefabricated components with limited structural function, or in components where improving thermal properties and utilizing waste are more important than maximizing compressive strength. This interpretation aligns with the multi-criteria approach advocated in more recent studies on the recycling of mineral waste in cementitious materials [1,3,4,18,19,20].

## 7. Conclusions

Decreases in mechanical strength values were observed—specifically, a (22–37)% reduction in mean cube compressive strength (f_cm,cube_) compared to reference series samples and an (8–11)% increase in splitting tensile strength (f_ct_). The selected microscopic images revealed local air macropores, including individual pores with visible dimensions exceeding approximately 300 μm. These observations may partly explain the reduction in compressive strength; however, the pore-size distribution and total pore volume were not quantified. The selected microscopic fields showed individual fibers and fiber bundles embedded in the cement matrix. The degree of fiber dispersion and the occurrence of agglomeration were not quantified.

The modified concretes exhibited increased water absorption and insufficient freeze–thaw resistance under the adopted test conditions, indicating that further mixture optimization is required before considering their use in environmentally exposed elements.

Microscopic analyses of the cement composite’s structure revealed macrostructural alterations, specifically the presence of detrimental air macropores with diameters exceeding 300 μm. These macropores contributed to a reduction in the concrete’s mean cube compressive strength (f_cm,cube_) by (22–37)% compared to reference samples 26/LBW/4-R. Furthermore, the existence of these air macropores led to an increase in the water absorption (n_w_) of the concrete samples, with observed values ranging from 6.0% to 8.68%. Given this situation, any structural component made from the proposed concrete mix will necessitate an additional protective coating to prevent water penetration, which acts as an environmental aggressor to the structure. This coating (or coatings) must ensure the material maintains a specific level of watertightness. Cracks were observed within the mineral aggregate (2.0/8.0 mm fraction granite), suggesting strong adhesion between the cement matrix and the crushed aggregate particles. No material imperfections were detected in the concrete’s microstructure, specifically no agglomeration of granite dust particles on the surface of the crushed coarse granite aggregate grains.

Laboratory tests on the material’s thermal properties clearly demonstrated the advantages of incorporating waste mineral wool fibers into concrete mixes. The material’s thermal conductivity coefficient (λ) decreased, which is advantageous as it improves the material’s thermal insulation properties (a lower (λ) parameter indicates better thermal insulation for vertical and/or horizontal building partitions). Reference samples R recorded a lambda (λ) value of 1.3461 W/(m·K), which was the highest value observed in this series of tests. The concrete samples from series 26/LBW/4-M1 yielded the most favorable results, with a lambda (λ) value of 1.1988 W/(m·K), marking an 11% reduction in the (λ) parameter. Composite samples demonstrated reductions in lambda (λ) values of 7% and 8%, respectively, when compared to the reference samples. The reduction in the thermal conductivity coefficient is advantageous for the composite, as it enhances its thermal insulation properties. The principal scientific contribution of the study is the identification of a material trade-off in which the reduction in thermal conductivity was accompanied by less favorable compressive-strength, water-absorption, and freeze–thaw-resistance performance.

Waste mineral wool, recovered from the demolition of heating system insulation, can be incorporated as an additive or admixture in cement concrete mixes. For this project, mineral wool was added at 12%, 16%, and 20% by mass of the CEM I 32.5R Portland cement binder (a hydraulic binder free of powdery additives such as siliceous fly ash, silica fume, and ground granulated blast-furnace slag). Dosing above 20% by mass of CEM I cement binder will necessitate a liquid chemical admixture to enhance the concrete mix’s fluidity, for instance, using polycarboxylate or melamine-based superplasticizers. The inclusion of waste mineral wool fibers was qualitatively associated with greater difficulty in placing and compacting the modified mixtures; however, the additional compaction effort was not quantified within the experimental program. Extended compaction on the vibrating table can lead to “bleeding” and segregation of the 2.0/8.0 mm coarse crushed granite aggregate. This means that some of the cement paste migrates to the upper layers of the mix, resulting in cement paste exuding on the sample’s top surface and side walls, which compromises the aesthetic quality of the samples and concrete surface, and causes an uneven distribution of cement paste within the concrete mix’s microstructure. The material modification using waste mineral wool led to a reduction in the workability properties of the concrete mix, specifically its workability, pumpability, and the ability to incorporate the mix into formwork containing densely spaced structural reinforcement. Additionally, at the interface between the mineral wool fiber and the binder matrix, zones of increased porosity (detrimental air macropores) can form, disrupting the continuity of the cement composite’s structure (material imperfections). With higher proportions of the additive, fiber agglomeration may also occur, leading to localized structural inhomogeneities and a further increase in water absorption.

The developed concrete mix formulations provide a foundation for further scientific investigation into the composite material’s durability across a wider spectrum. This includes examining extended concrete durability under UVA and UVB radiation, its performance in elevated salt spray concentrations (representing excessive chloride ion aggression), and its response to thermal shock (extreme temperatures ranging from Tmin = −80 °C to Tmax = 200 °C) within both reduced and elevated temperature conditions (cryogenic concrete).

However, the practical application of the investigated concretes should be considered with caution, as the modified series exhibited increased water absorption, reduced mean compressive strength, and insufficient frost resistance under the adopted test conditions.

The investigated concrete mixtures may have potential for selected prefabricated applications, particularly in sheltered elements with limited structural function, where reduced thermal conductivity and the utilization of waste materials are relevant. Their use in exposed or load-bearing elements would require further optimization of the mixture composition and verification of the relevant mechanical and durability requirements. Further optimization of workability would also be required before considering applications involving highly flowable or self-compacting concretes.

The investigated formulations may provide a basis for further mix optimization for specific applications. Any adjustment to particular environmental exposure conditions would require additional verification of the relevant mechanical, moisture-related, and durability properties.

Further research is also required to determine the relationships between mineral-wool dosage, mechanical performance, and thermal conductivity, as well as to identify a dosage range that provides a favorable balance between thermal benefits and mechanical and durability requirements.

In a broader perspective, future studies could also employ data-driven optimization and microstructure-informed modeling to support the selection of mixture compositions and predict the resulting mechanical, thermal, and durability properties. Active-learning strategies may help identify the most informative experimental configurations, thereby reducing extensive trial-and-error testing. In contrast, models incorporating quantitatively characterized pore and fiber distributions could improve the interpretation and prediction of structure–property relationships in mineral-wool-modified cementitious composites [21,22].

## Figures and Tables

**Figure 12:**
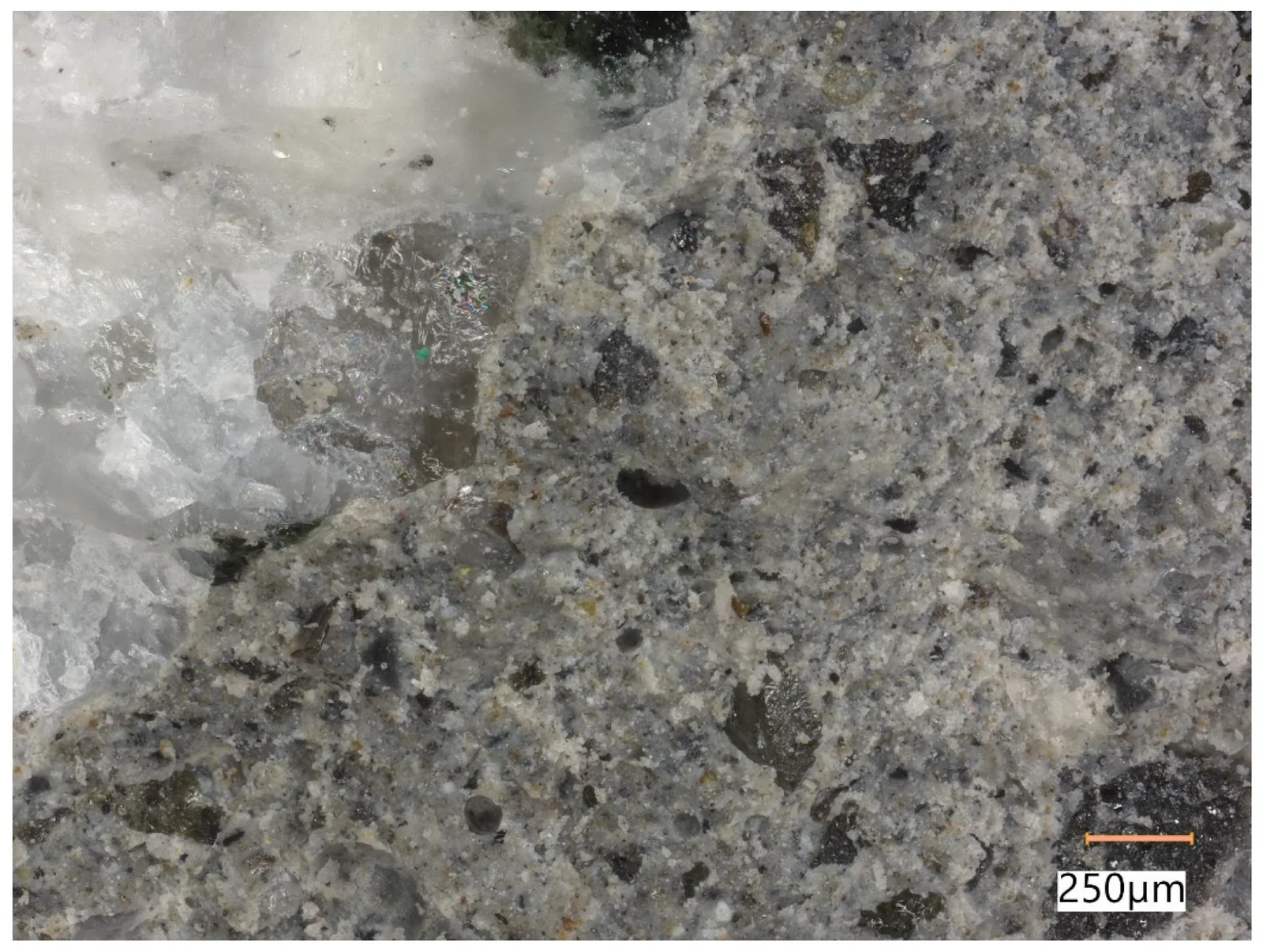
Microstructure image of the fracture surface of a cement concrete sample without material modification. An example of concrete microstructure imaging, showing the boundary between a coarse granite aggregate (2.0/8.0 mm) and the cement matrix (author’s own work).

**Figure 13:**
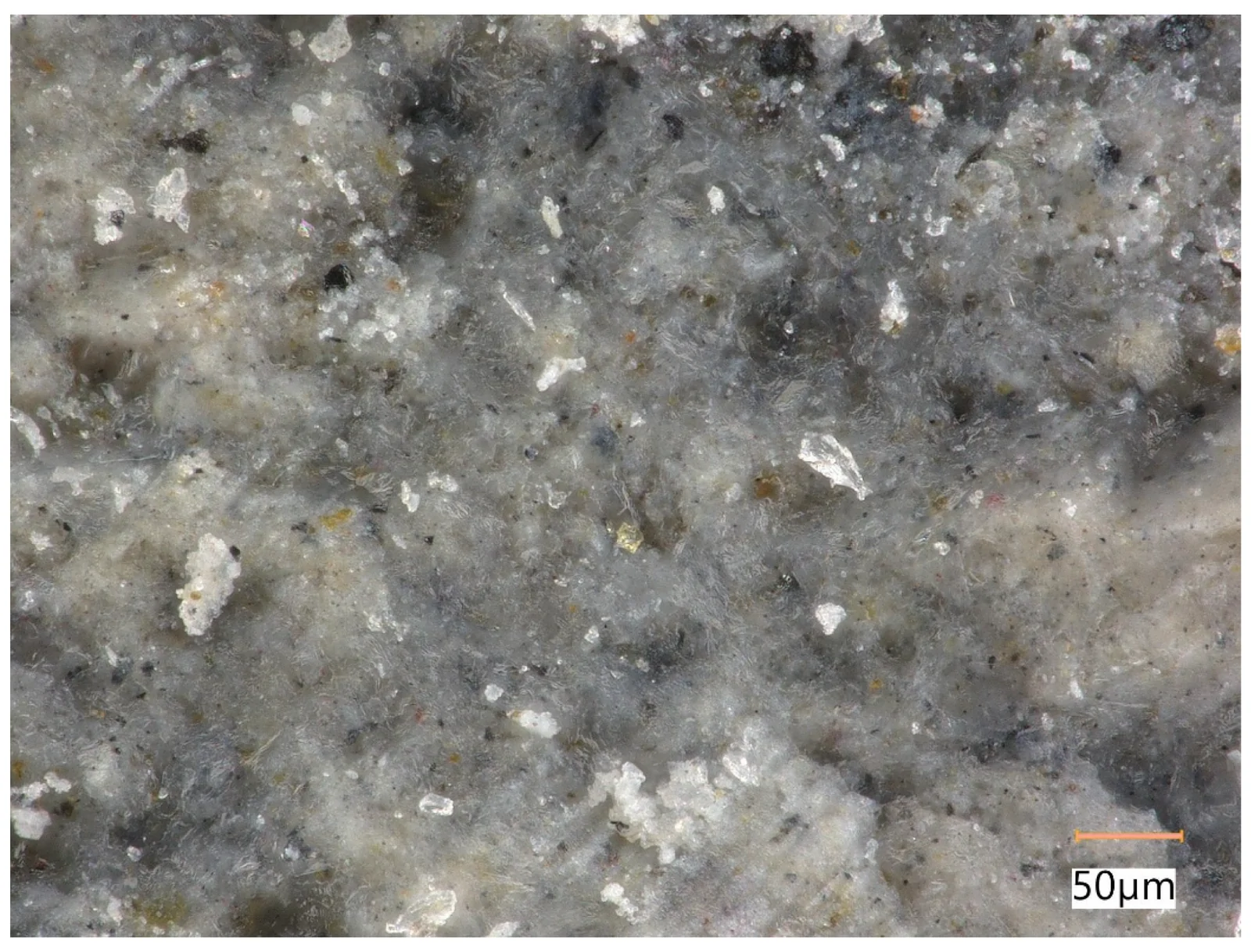
Microstructure image of the fracture surface of a cement concrete sample without material modification. An example of concrete microstructure imaging, showing the cement matrix (author’s own work).

**Figure 14:**
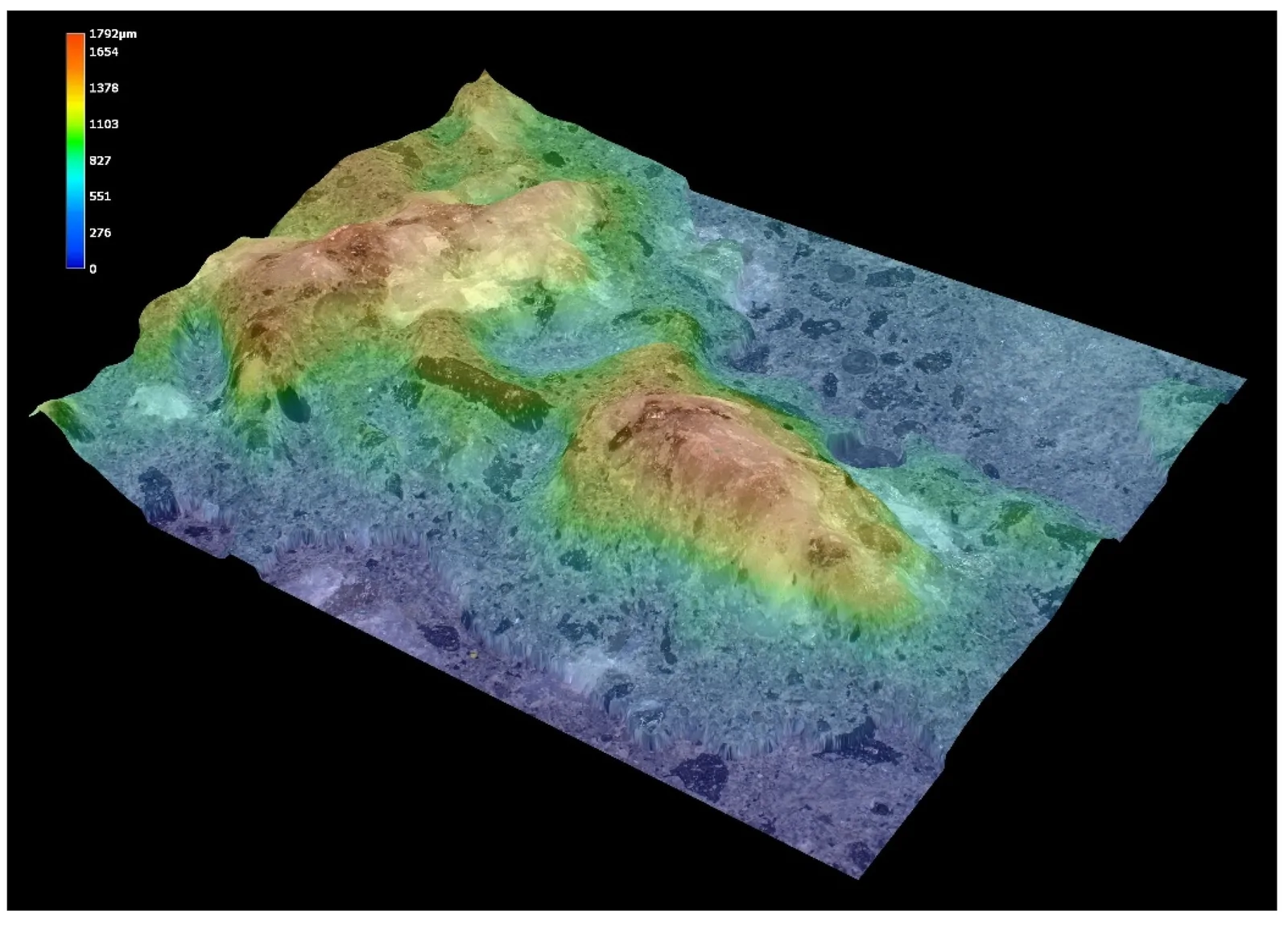
A 3D image obtained by scanning the microstructure of a fractured cement concrete sample without material modification. An example of 3D imaging of concrete microstructure surface topography in “rainbow” colors on a hypsometric scale (author’s own work).

**Figure 15:**
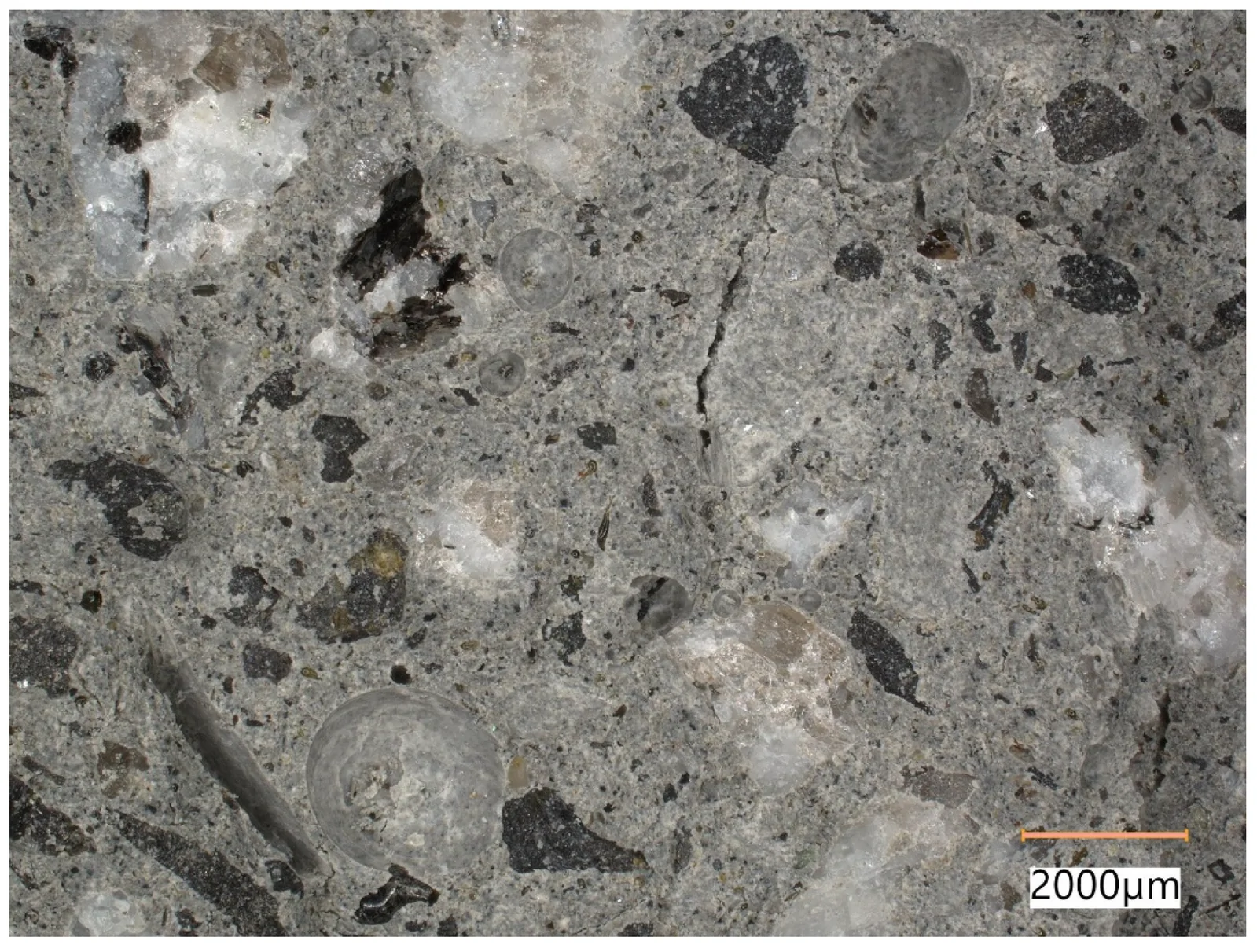
Representative fracture-surface image of the M1 concrete containing 12% mineral wool, showing granite and basalt aggregate grains, the cement matrix, and local air macropores (author’s own work).

**Figure 16:**
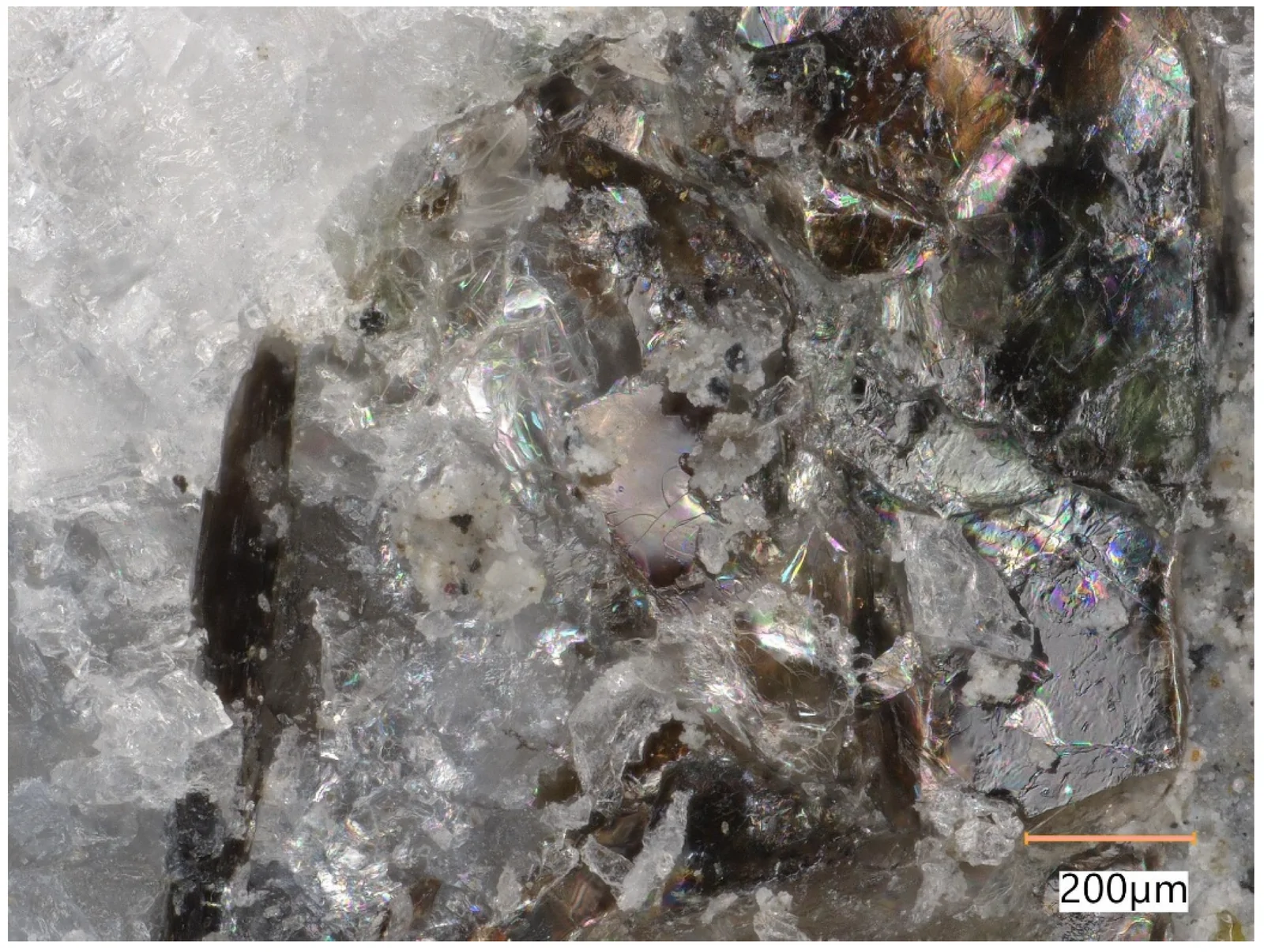
Representative fracture-surface image of the M1 concrete containing 12% mineral wool, showing the surface morphology of a coarse granite aggregate grain (author’s own work).

**Figure 17:**
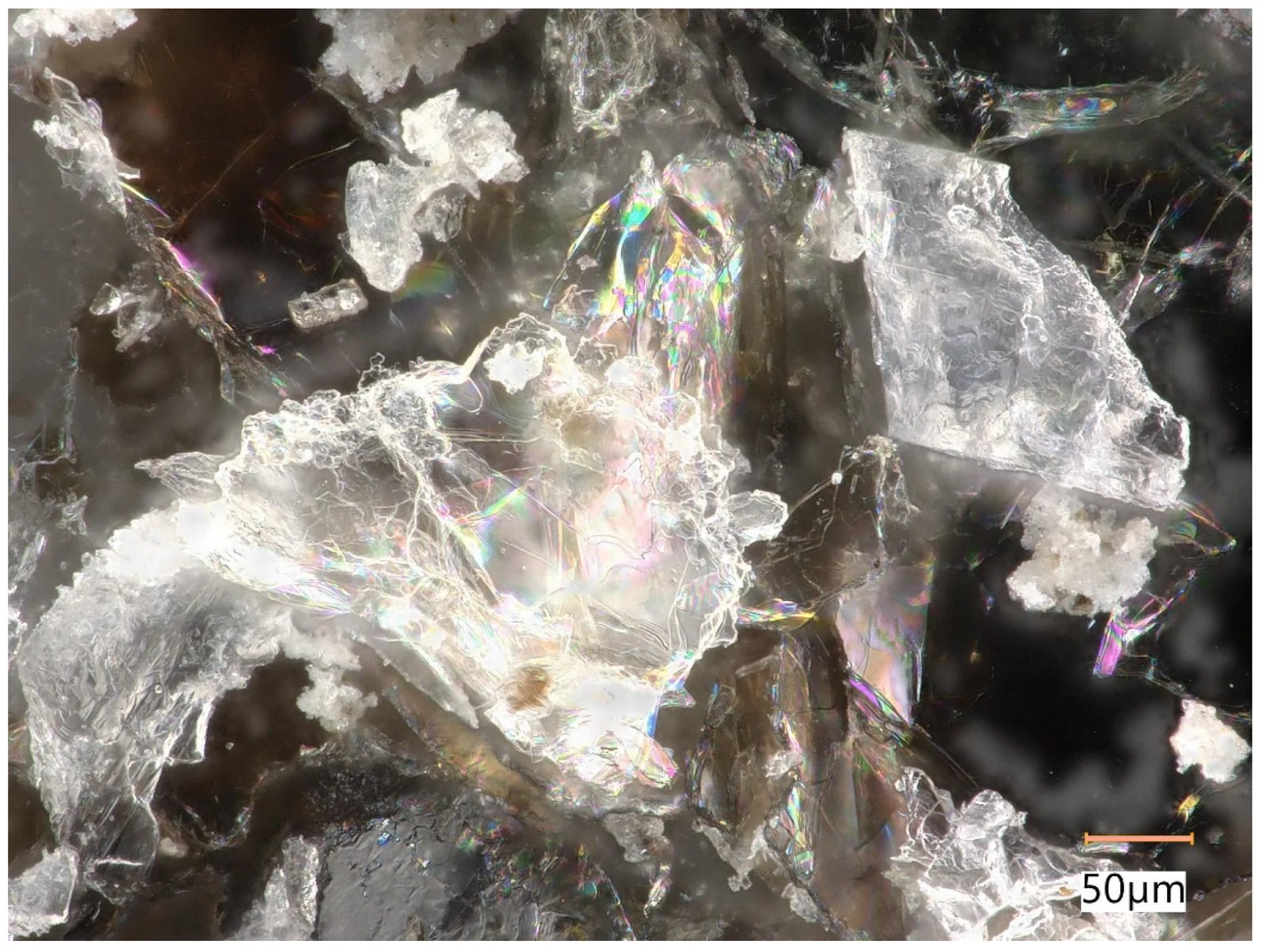
Representative fracture-surface image of the M1 concrete containing 12% mineral wool, showing fine basalt aggregate grains and the cement matrix (author’s own work).

**Figure 18:**
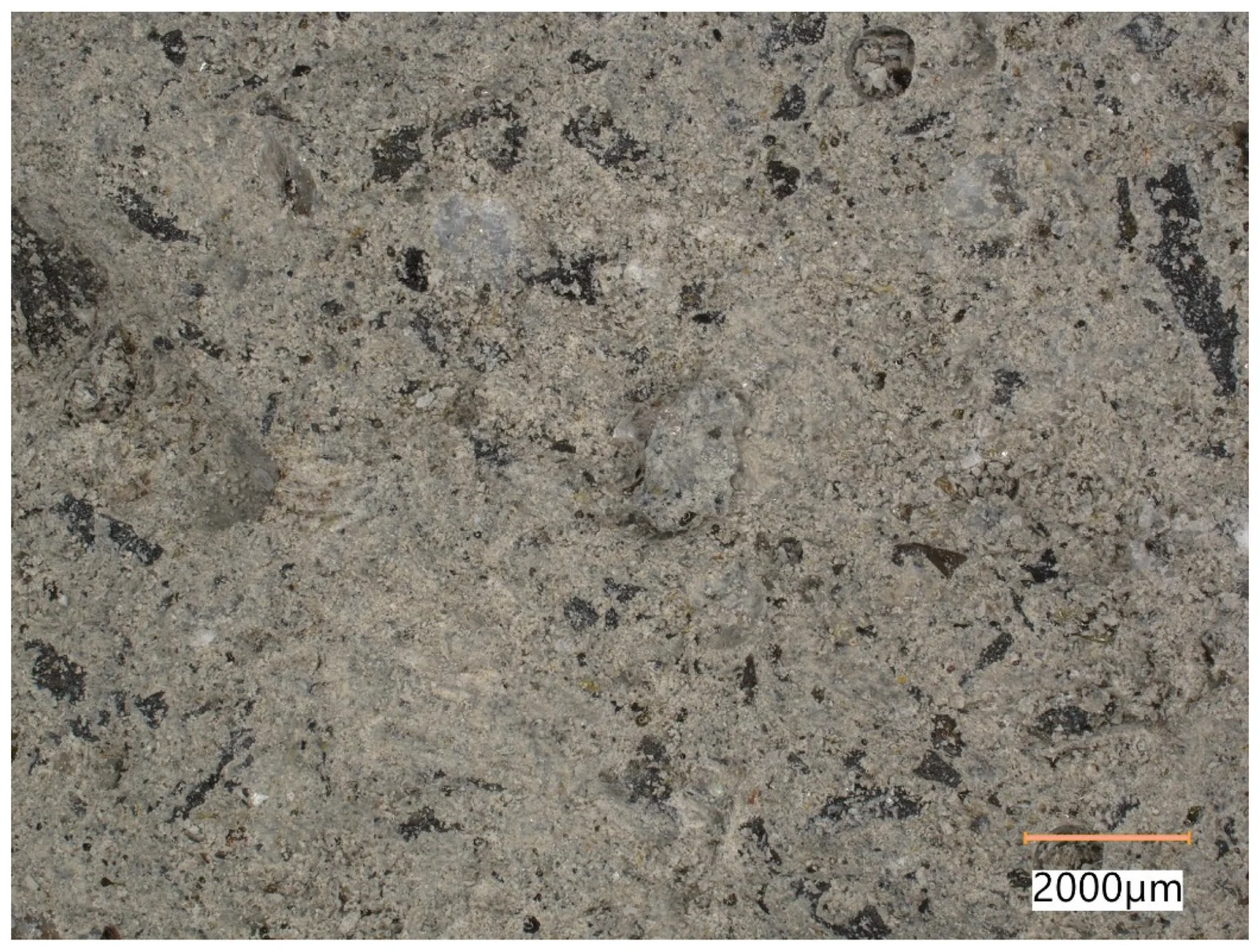
Representative fracture-surface image of the M2 concrete containing 16% mineral wool, showing fine basalt aggregate grains and the cement matrix (author’s own work).

**Figure 19:**
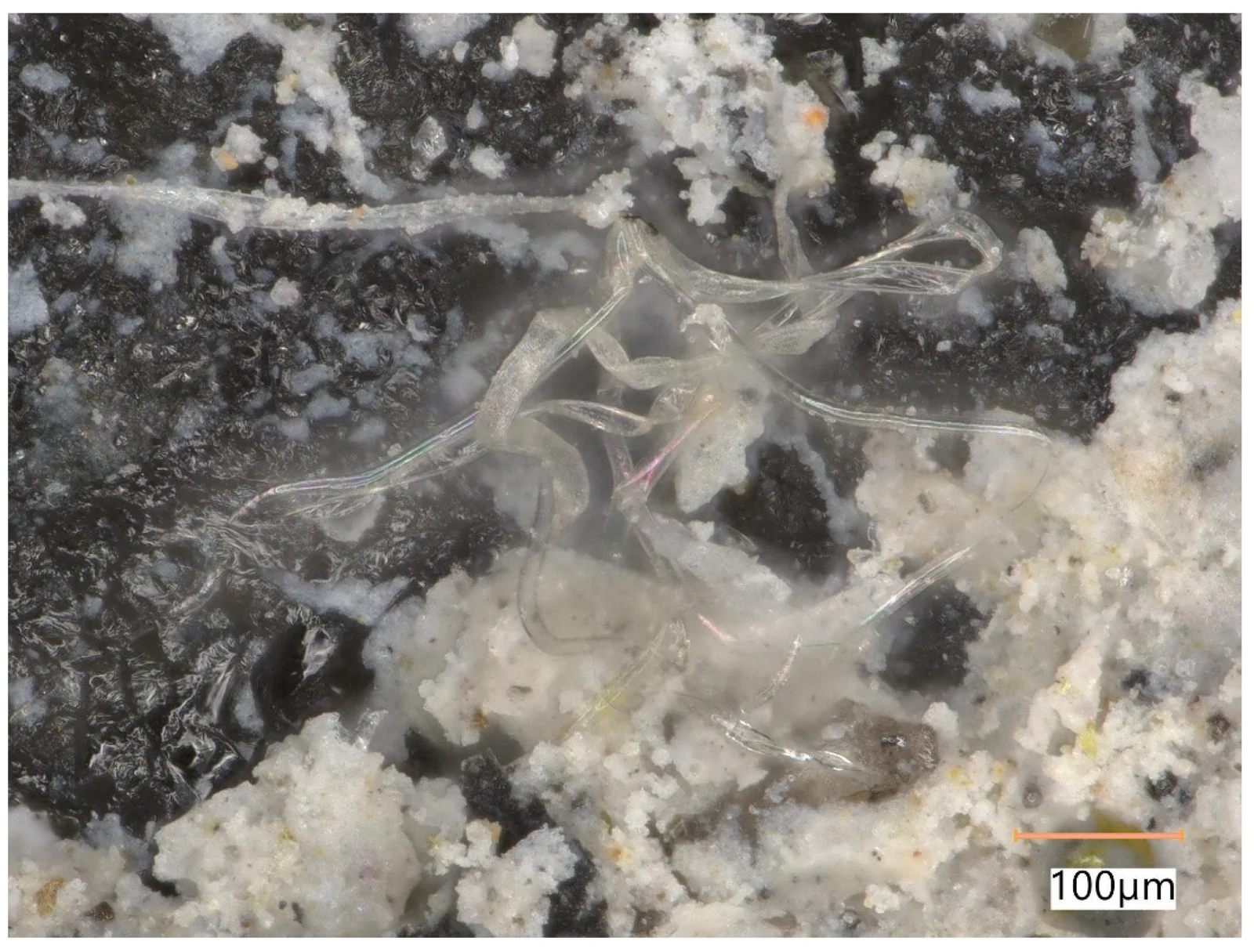
Representative fracture-surface image of the M2 concrete containing 16% mineral wool, showing fine basalt aggregate grains, the cement matrix, and mineral-wool fibers (author’s own work).

**Figure 20:**
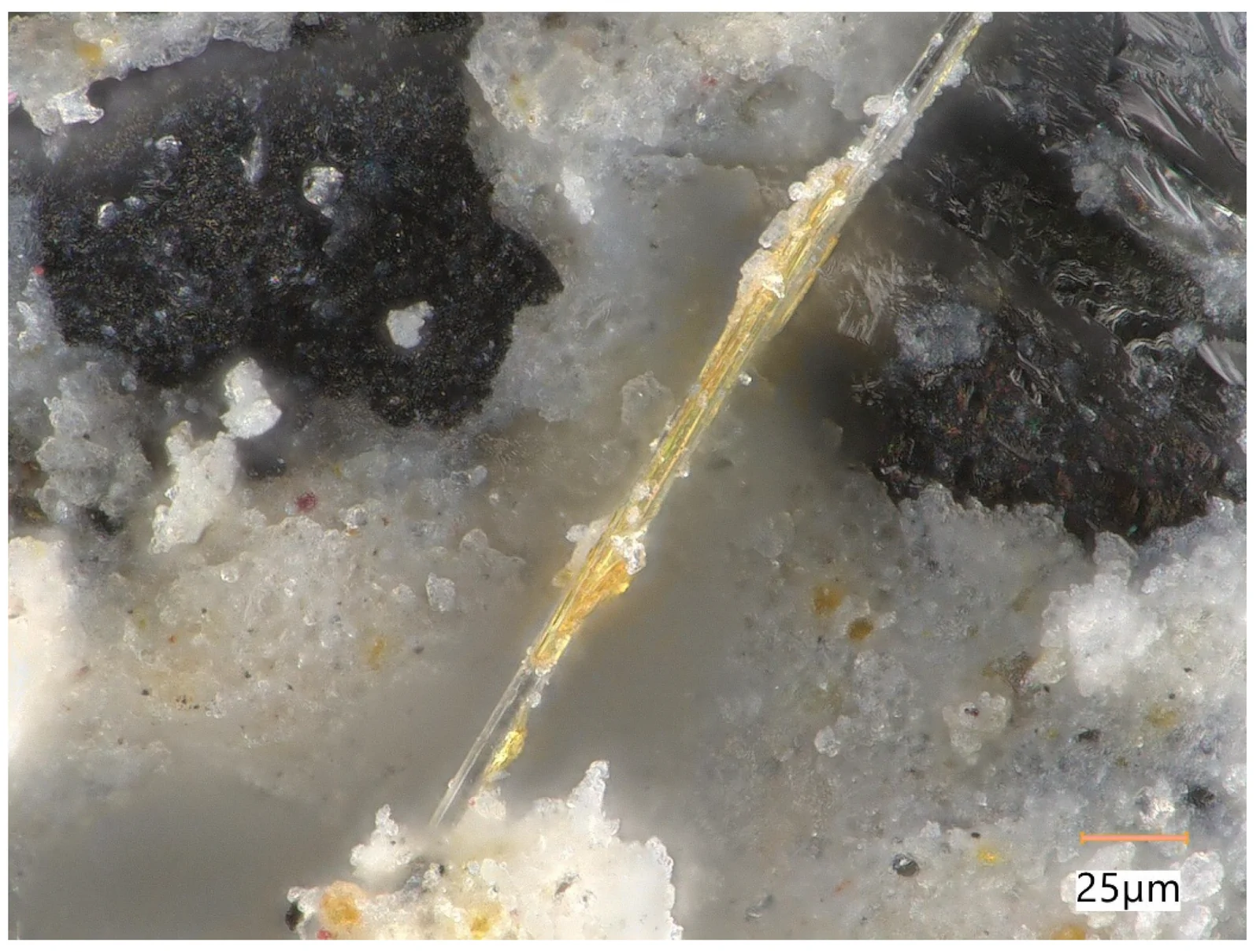
Representative fracture-surface image of the M2 concrete containing 16% mineral wool, showing an individual mineral-wool fiber embedded in the cement matrix (author’s own work).

**Figure 21:**
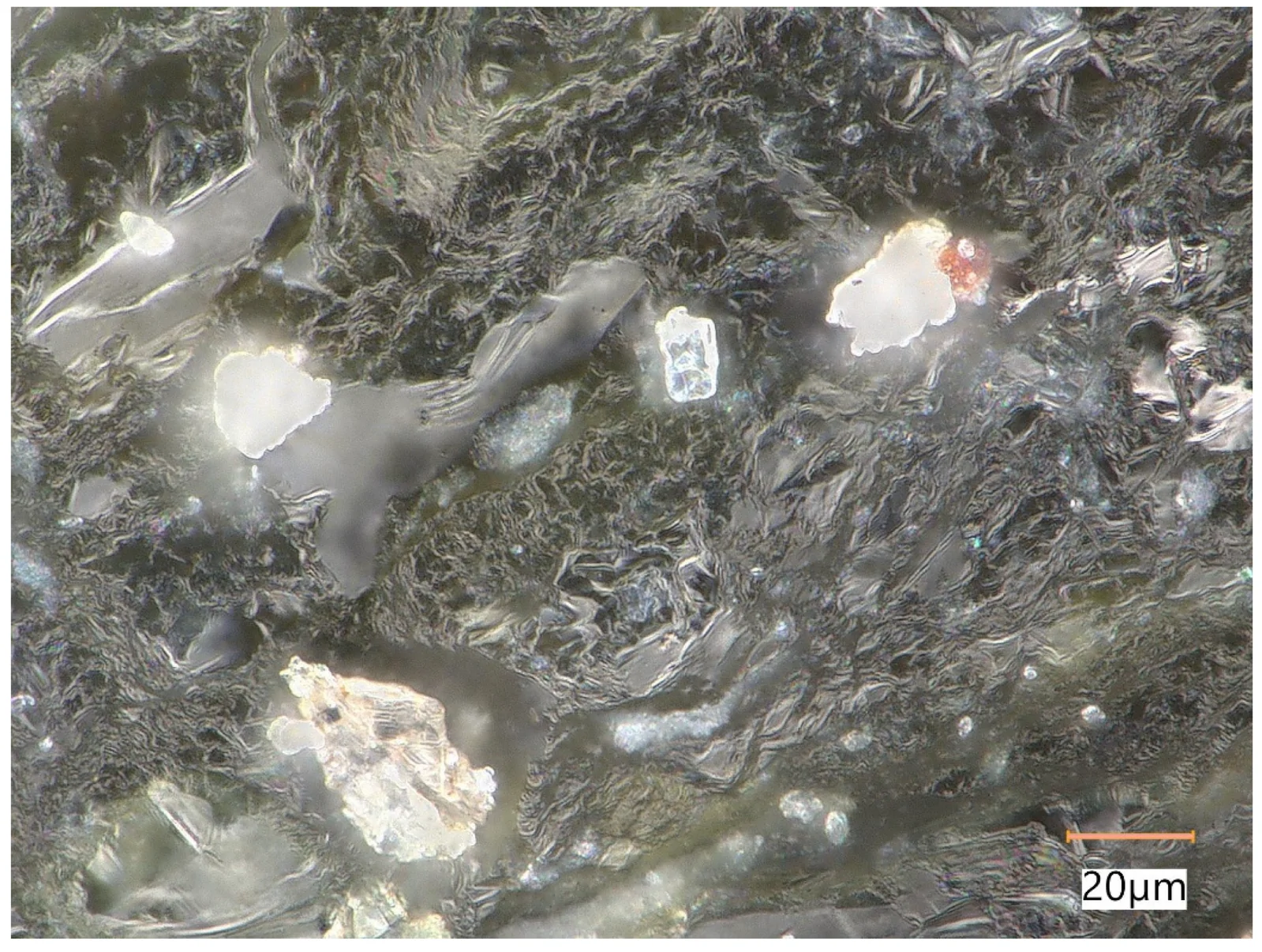
Representative fracture-surface image of the M2 concrete containing 16% mineral wool, showing fine basalt aggregate grains within the cement matrix (author’s own work).

**Figure 22:**
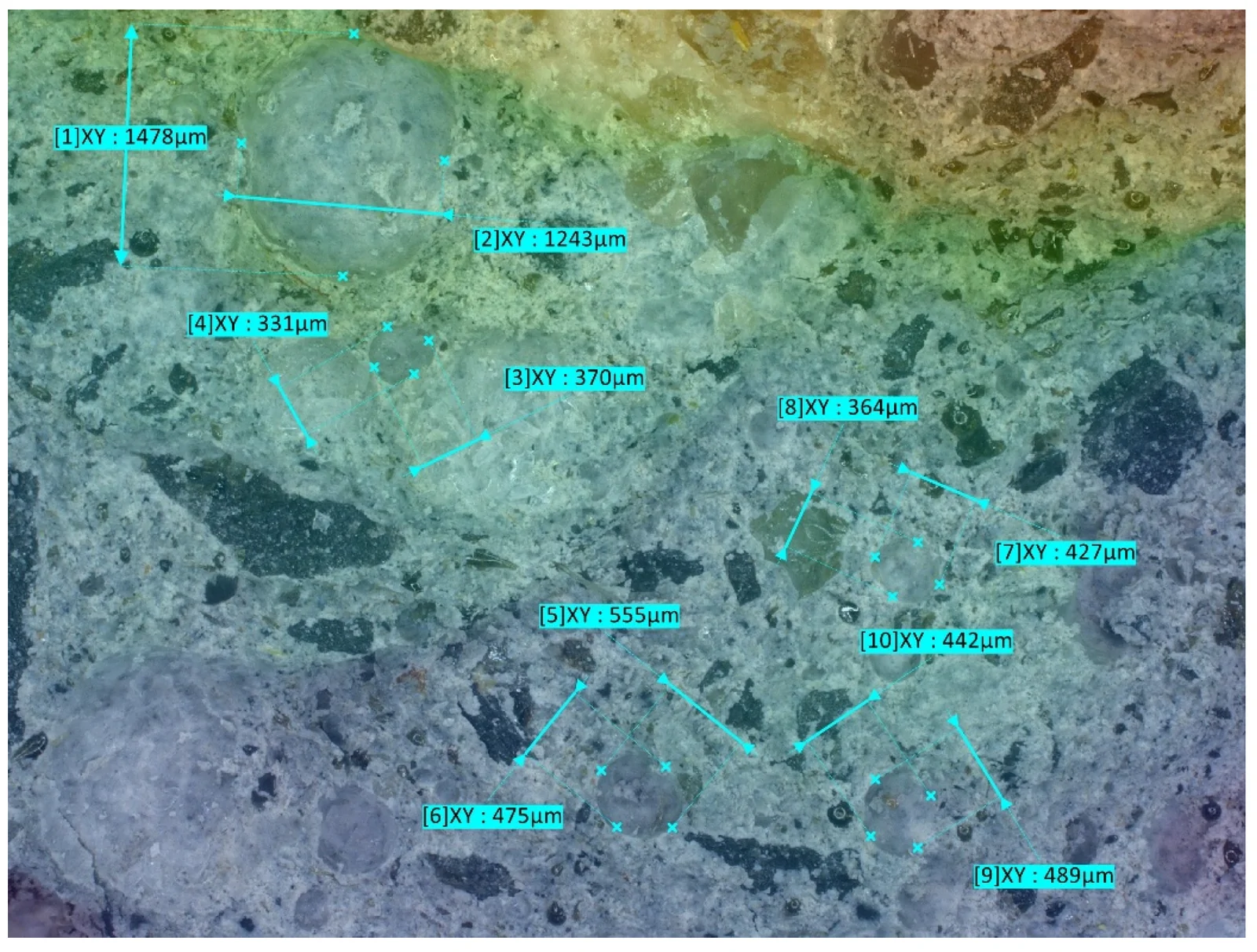
Hypsometric image of the M3 concrete fracture surface containing 20% mineral wool, showing a local air macropore and an indicative measurement of its diameter (author’s own work).

**Figure 23:**
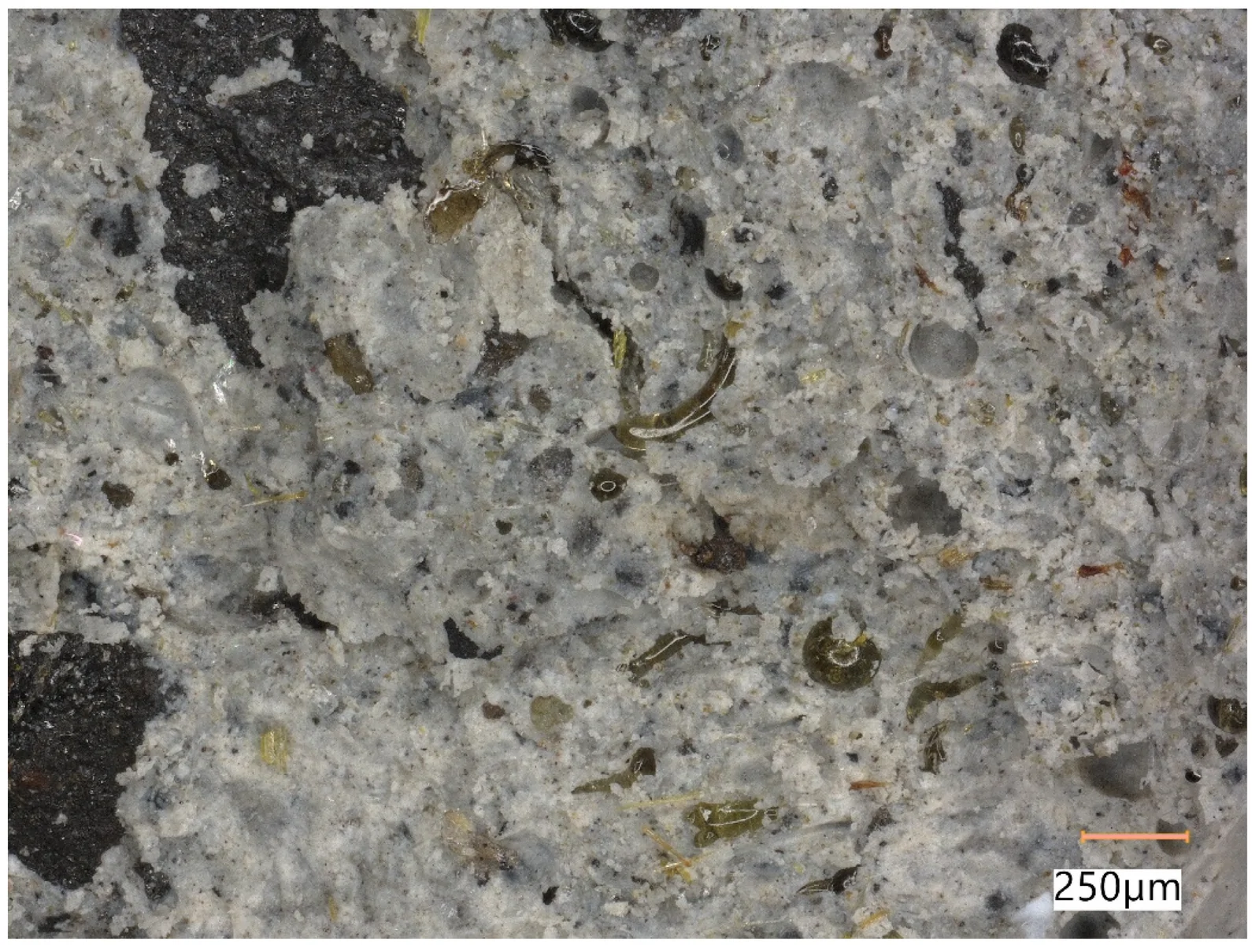
Representative fracture-surface image of the M3 concrete containing 20% mineral wool, showing mineral-wool fibers embedded in the cement matrix (author’s own work).

**Figure 24:**
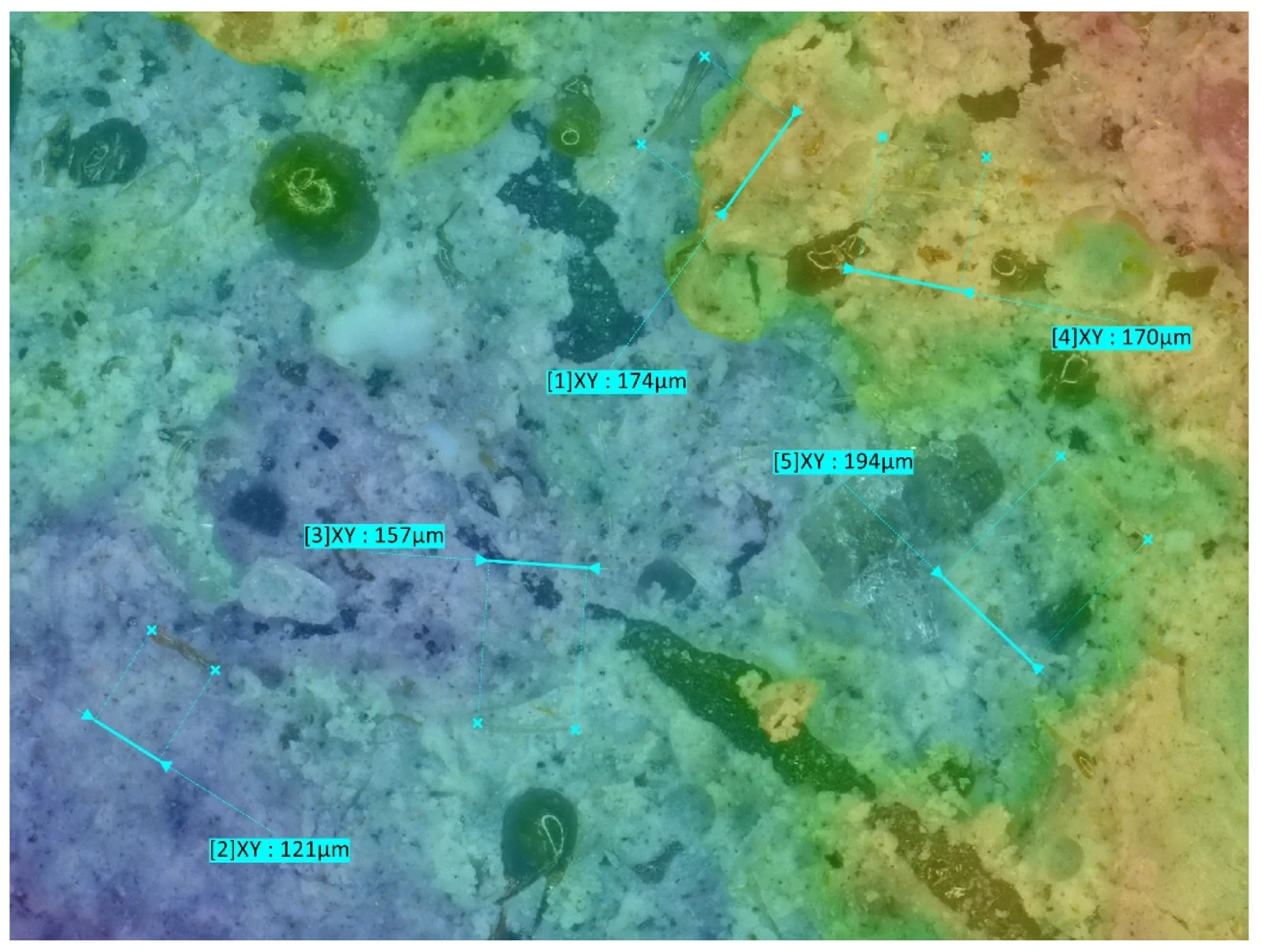
Representative fracture-surface image of the M3 concrete containing 20% mineral wool, showing an individual visible mineral-wool fiber segment with an indicative length measurement (author’s own work).

**Figure 25:**
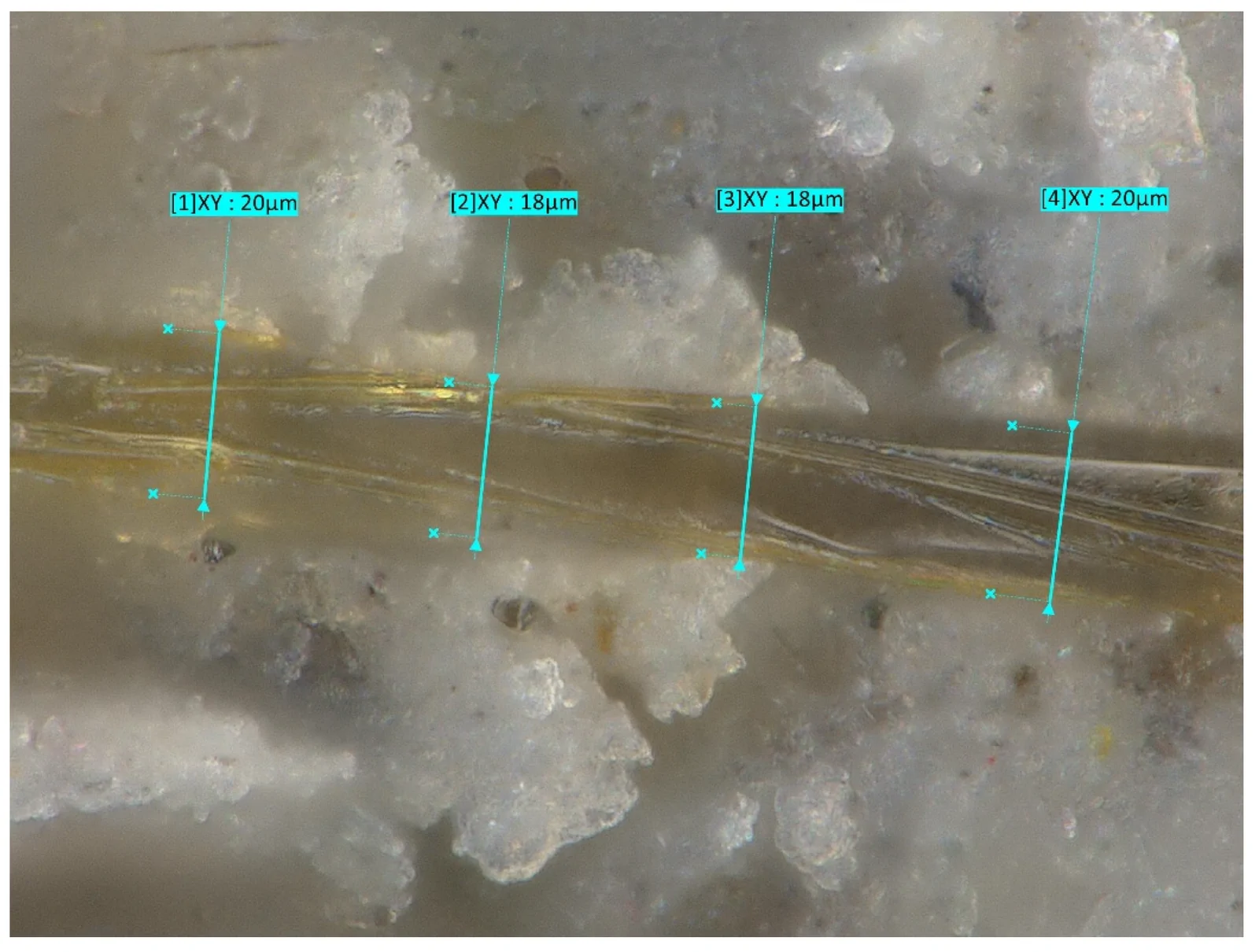
Representative fracture-surface image of the M3 concrete containing 20% mineral wool, showing fine basalt aggregate grains, the cement matrix, and mineral-wool fibers (author’s own work).

**Table 1:** Concrete mix compositions (author’s own work).

No.	Concrete Mix Component	Component Quantity in Concrete Mix [kg/m^3^]			
		R	M1	M2	M3
**1.**	Mixing water, tap water; pH = 7.0	197	197	197	197
**2.**	Hydraulic Binder—General Purpose Cement CEM I 32.5R	405	405	405	405
**3.**	Liquid Admixture No. 1—A highly plasticizing chemical admixture based on an aqueous solution of modified polycarboxylic ethers (1.50% by mass of cement binder)	6.08	6.08	6.08	6.08
**4.**	Crushed fine aggregate—Basalt, 0.0/2.0 mm fraction group	936	936	936	936
**5.**	Crushed coarse aggregate—Granite, 2.0/8.0 mm fraction group	972	972	972	972
**6.**	Waste mineral wool (12%, 16%, and 20% of the reference mass of the cement binder)	0	48.6	64.8	81.0
**7.**	Air content in the concrete mixture	4.5	4.6	4.7	4.7
**8.**	Air content in concrete	5.0	5.2	5.4	5.4

## Data Availability

The original contributions presented in this study are included in the article/Supplementary Material. Further inquiries can be directed to the corresponding author.

## Supplementary Materials

The following supporting information can be downloaded at https://www.mdpi.com/article/10.3390/ma19163502/s1. Table S1: Technology for concrete samples of series R, M1, M2, and M3. Categorization by stage and task types; Table S2: Tabular summary of the designed concrete mix compositions (including quantitative adjustments post-laboratory testing): reference formulation (R) and three material-modified formulations using a waste component (M1, M2, M3); Table S3: Descriptive statistics for the measured characteristic compressive strength (f_ck,cube_) values for samples from series R, M1, M2, and M3; Table S4: Descriptive statistics for measured splitting tensile strength (f_ct_) values for series R, M1, M2, and M3 samples.; Table S5: Descriptive statistics for measured bulk density (ρ) values for samples from series R, M1, M2, and M3; Table S6: Descriptive statistics for the measured surface water absorption (n_w_) values of samples from series R, M1, M2, and M3; Table S7: Descriptive statistics for the measured characteristic compressive strength (f_ck,cube_) values of samples from series R, M1, M2, and M3 (internal concrete frost resistance test before 100 freeze–thaw cycles); Table S8: Descriptive statistics for the characteristic compressive strength after 100 F-T cycles (internal frost resistance of concrete) (f_ck,cube_); Table S9: Descriptive statistics for the measured mass change values of water-saturated samples from series R, M1, M2, and M3 (external frost resistance test of concrete before 100 F-T cycles); Table S10: Descriptive statistics for the measured mass change values of water-saturated samples from series R, M1, M2, and M3 (external frost resistance test of concrete after 100 F-T cycles); Table S11: Descriptive statistics for the measured thermal conductivity coefficient (λ) values for samples from series R, M1, M2, and M3; Table S12: Descriptive statistics for the measured specific heat (c_p_) values of material for R, M1, M2, and M3 series samples; Table S13: Descriptive statistics for the measured thermal diffusivity (a) values observed in samples from series R, M1, M2, and M3.
