# Supplementary material for: Concretes Modified with Insulation Wool Recovered from Recycled Heating Pipes
Source: Materials (Basel). 2026 Aug 18;19(16):3502. doi: 10.3390/ma19163502 (PMC13514968; doi:10.3390/ma19163502)
Supplement: Supplementary file 1 [file materials-19-03502-s001.zip › materials-4452610-supplementary.pdf]

SUPPLEMENTARY MATERIAL

For the article "Concrete Modified With Insulation Wool Recycled From Heating Pipes"

1) CONCRETE SAMPLE TECHNOLOGY—SUMMARY DESCRIPTION

A summary description is provided for the technology used to create cement concrete samples, which were designed according to formulations R, M1, M2, and M3. A tabular description was employed, categorizing information by stage type, task number within each stage, task type, the ingredients used, and the main and auxiliary equipment necessary for completing tasks at each stage of the developed technology.

Table S1. Technology for concrete samples of series R, M1, M2, and M3. Categorization by stage and task types (author's own work).

**Technology for the reference mix and material-modified mixes—R, M1, M2, M3.**								
No.	Stage Number	Stage Type	Task Number	Task Type	Ingredient Type	Equipment Type	Task Duration	Stage Duration
							[s], [min], [h], day	[s], [min], [h], day
1.	1	Preparation of weighed quantities of all concrete mix components	1	Preparation of weighed quantities of liquid components	Mixing water, tap water; pH = 7.0	Graduated laboratory cylinder and beaker, calibrated precision balance	10–15 min	20–30 min
2.					Liquid Admixture No. 1—A highly plasticizing chemical admixture based on an aqueous solution of modified polycarboxylic ethers (1.50% by mass of cement binder)	Graduated laboratory beaker, calibrated analytical balance, calibrated glass pipette		
4.			2	Preparation of weighed quantities of dry components	Portland cement CEM I 32.5R	Graduated laboratory cylinder, calibrated precision balance, spatula	10–15 min	
5.					Basalt, 0.0/2.0 mm fraction group	Graduated laboratory cylinder, calibrated precision balance, spatula		

6.					Granite, 2.0/8.0 mm fraction group	Graduated laboratory cylinder, calibrated precision balance, spatula		
7.	2	Preparation of molds for concrete mix samples	1	Checking the shape and dimensions of molds for standard 150x150x150 mm type "B" cubic samples, in accordance with standard requirements	- - - - -	Calibrated linear gauge with a measuring range of up to 500 mm	Depending on the number of molds	Depending on the number of molds
8.						Calibrated angle gauge		
9.						Calibrated feeler gauge with a measuring range of 0.03–1.00 mm		
10.			2	Coating the inner walls of the molds with an anti-adhesive agent—specifically, for 150x150x150 mm type "B" cubic sample molds—applied by spray	- - - - -	Plastic molds	Depending on the number of molds	
11.			3	Checking the shape and dimensions of molds for standard 100x100x100 mm type "C" cubic samples, in accordance with standard requirements	- - - - -	Calibrated linear gauge with a measuring range of up to 500 mm	Depending on the number of molds	
12.						Calibrated angle gauge		
13.						Calibrated feeler gauge with a measuring range of 0.03–1.00 mm		
14.			4	Coating the inner walls of the molds with an anti-adhesive agent—specifically, for 100x100x100 mm type "C" cubic sample molds—applied by spray	- - - - -	Plastic molds	Depending on the number of molds	
15.			5	Checking the shape and dimensions of the molds for standard (100x100x500) mm beam samples, as per standard requirements	- - - - -	Calibrated linear gauge with a measuring range of up to 1000 mm	Depending on the number of molds	
16.						Calibrated angle gauge		
17.						Calibrated feeler gauge with a measuring range of 0.03–1.00 mm		

18.			6	Spraying the inner walls of the (100x100x500) mm beam sample molds with an anti-adhesive agent	- - - - -	Plastic molds	Depending on the number of molds	
19.	3	Preparing a dry mixture of the formulation ingredients	1	Loading the 2.0/8.0 mm granite crushed aggregate into the hopper of the horizontal laboratory mixer	Granite, 2.0/8.0 mm fraction group	Horizontal laboratory mixer	2–4 min	11–20 min
20.			2	Mixing crushed granite aggregate of the 2.0/8.0 mm fraction group	Granite, 2.0/8.0 mm fraction group	Horizontal laboratory mixer	1–2 min	
21.			3	Adding basalt of the 0.0/2.0 mm fraction group to the horizontal laboratory mixer's container (hopper)	Basalt, 0.0/2.0 mm fraction group	Horizontal laboratory mixer	1–2 min	
22.			4	Mixing crushed fine basalt aggregate of the 0.0/2.0 mm fraction group	Basalt, 0.0/2.0 mm fraction group	Horizontal laboratory mixer	1–2 min	
23.			5	Adding Portland cement to the horizontal laboratory mixer's container (hopper) CEM I 32.5R	Portland cement CEM I 32.5R	Horizontal laboratory mixer	1–2 min	
24.			6	Mixing Portland cement CEM I 32.5R	Portland cement CEM I 32.5R	Horizontal laboratory mixer	1–2 min	
25.			7	Mixing all dry ingredients in the hopper of the horizontal laboratory mixer	Granite, 2.0/8.0 mm fraction group	Horizontal laboratory mixer	4–6 min	
26.					Soda-lime glass granules, 0.0/2.0 mm fraction group			
27.					Portland cement CEM I 32.5R			
28.	4	Preparation of the concrete mix	1	Mixing the aqueous solution with admixture No. 1	Mixing water, tap water; pH = 7.0	Graduated laboratory beaker	12–16 min	16–24 min
						Calibrated analytical balance		

29.					Liquid Admixture No. 1—A highly plasticizing chemical admixture based on an aqueous solution of modified polycarboxylic ethers (1.50% by mass of cement binder)	Calibrated glass pipette		
30.						Magnetic stirrer		
31.			3	Adding tap water (mixing water) with a pH of 7.0 to the container (hopper) of the horizontal laboratory mixer	Mixing water, tap water; pH = 7.0	Horizontal laboratory mixer	1–2 min	
36.			4	Mixing all dry ingredients and tap water (mixing water) with a pH of 7.0 in the container (hopper) of the horizontal laboratory mixer	Mixing water, tap water; pH = 7.0	Horizontal laboratory mixer	1–2 min	
37.					Portland cement CEM I 32.5R			
38.					Soda-lime glass granules, 0.0/2.0 mm fraction group			
39.					Granite, 2.0/8.0 mm fraction group			
40.			5	Adding the aqueous solution with Admixture No. 1 to the container (hopper) of the horizontal laboratory mixer	Aqueous solution with liquid admixture No. 1—A highly plasticizing chemical admixture based on an aqueous solution of modified polycarboxylic ethers (1.50% by mass of cement binder)	Horizontal laboratory mixer	1–2 min	
41.								
42.								
44.			6	Mixing all dry and liquid components in the horizontal laboratory mixer's hopper (feed bin)	Mixing water, tap water; pH = 7.0	Horizontal laboratory mixer	1–2 min	
45.					Aqueous solution with liquid admixture No. 1—A highly plasticizing chemical admixture based on an aqueous solution of modified polycarboxylic ethers (1.50% by mass of cement binder)			
46.					Portland cement CEM I 32.5R			

48.					Granite, 2.0/8.0 mm fraction group			
49.					Granite, 2.0/8.0 mm fraction group			
50.		Preparation of concrete mix samples	1	Placing the concrete mix into molds	Mixing water, tap water; pH = 7.0	Plastic molds	10–15 min	10 min and 20 s to 15 min and 30 s
51.					Aqueous solution with liquid admixture No. 1—A highly plasticizing chemical admixture based on an aqueous solution of modified polycarboxylic ethers (1.50% by mass of cement binder)			
52.					Portland cement CEM I 32.5R	Trowel		
54.					Granite, 2.0/8.0 mm fraction group			
55.					Granite, 2.0/8.0 mm fraction group			
56.			2	Mechanical compaction of the concrete mix for layer No. 1	Mixing water, tap water; pH = 7.0	Vibrating table	10–15 s	
57.					Aqueous solution with liquid admixture No. 1—A highly plasticizing chemical admixture based on an aqueous solution of modified polycarboxylic ethers (1.50% by mass of cement binder)			
58.					Portland cement CEM I 32.5R	Steel float for finishing (standard "C" type cubic samples (100x100x100) mm)		
60.					Granite, 2.0/8.0 mm fraction group			
61.					Granite, 2.0/8.0 mm fraction group			
62.			3	Mechanical compaction of the concrete mix for layer No. 2	Mixing water, tap water; pH = 7.0	Vibrating table	10–15 s	
63.					Aqueous solution with liquid admixture No. 1—A highly plasticizing chemical admixture based on an aqueous solution of modified polycarboxylic ethers (1.50% by mass of cement binder)			
64.					Portland cement CEM I 32.5R	Steel trowel for finishing (standard type "B" cubic samples (150x150x150) mm,		
66.					Basalt, 0.0/2.0 mm fraction group			

67.					Granite, 2.0/8.0 mm fraction group	beam samples (150x150x600) mm)		
68.	6	Preparation of concrete samples	1	Demolding of concrete samples	Concrete	Compressor	Depending on the number of molds	> 672 h (demolding and 28 days)
69.						Compressor instrumentation		
70.			2	Curing of concrete samples	Concrete	Calibrated climatic chamber or pallet box with tap water	672 h (28 days)	
71.						Calibrated electronic thermo-hygrometer		
72.								

No.	Stage Number	Stage Type	Task Number	Task Type	Ingredient Type	Equipment Type	Task Duration	Stage Duration
							[s], [min], [h], day	[s], [min], [h], day
1.	1	Preparation of weighed quantities of all concrete mix components	1	Preparation of weighed quantities of liquid components	Mixing water, tap water; pH = 7.0	Graduated laboratory cylinder and beaker, calibrated precision balance	10–15 min	20–30 min
2.					Liquid Admixture No. 1—A highly plasticizing chemical admixture based on an aqueous solution of modified polycarboxylic ethers (1.50% by mass of cement binder)	Graduated laboratory beaker, calibrated analytical balance, calibrated glass pipette		
4.			2	Preparation of weighed quantities of dry components	Portland cement CEM I 32.5R	Graduated laboratory cylinder, calibrated precision balance, spatula	10–15 min	
5.					Basalt, 0.0/2.0 mm fraction group	Graduated laboratory cylinder, calibrated precision balance, spatula		
6.					Granite, 2.0/8.0 mm fraction group	Graduated laboratory cylinder, calibrated precision balance, spatula		
7.	2	Preparation of molds for concrete mix samples	1	Checking the shape and dimensions of molds for standard 150x150x150 mm type "B" cubic samples, in accordance with standard requirements	- - - - -	Calibrated linear gauge with a measuring range of up to 500 mm	Depending on the number of molds	Depending on the number of molds
8.						Calibrated angle gauge		

9.						Calibrated feeler gauge with a measuring range of 0.03–1.00 mm		
10.			2	Coating the inner walls of the molds with an anti-adhesive agent—specifically, for 150x150x150 mm type "B" cubic sample molds—applied by spray	- - - - -	Plastic molds	Depending on the number of molds	
11.			3	Checking the shape and dimensions of molds for standard 100x100x100 mm type "C" cubic samples, in accordance with standard requirements	- - - - -	Calibrated linear gauge with a measuring range of up to 500 mm	Depending on the number of molds	
12.						Calibrated angle gauge		
13.						Calibrated feeler gauge with a measuring range of 0.03–1.00 mm		
14.			4	Coating the inner walls of the molds with an anti-adhesive agent—specifically, for 100x100x100 mm type "C" cubic sample molds—applied by spray	- - - - -	Plastic molds	Depending on the number of molds	
15.			5	Checking the shape and dimensions of the molds for standard (100x100x500) mm beam samples, as per standard requirements	- - - - -	Calibrated linear gauge with a measuring range of up to 1000 mm	Depending on the number of molds	
16.						Calibrated angle gauge		
17.						Calibrated feeler gauge with a measuring range of 0.03–1.00 mm		
18.			6	Spraying the inner walls of the (100x100x500) mm beam sample molds with an anti-adhesive agent	- - - - -	Plastic molds	Depending on the number of molds	
19.	3	Preparing a dry mixture of the formulation ingredients	1	Loading the 2.0/8.0 mm granite crushed aggregate into the hopper of the horizontal laboratory mixer	Granite, 2.0/8.0 mm fraction group	Horizontal laboratory mixer	2–4 min	11–20 min

20.			2	Mixing crushed granite aggregate of the 2.0/8.0 mm fraction group	Granite, 2.0/8.0 mm fraction group	Horizontal laboratory mixer	1–2 min	
21.			3	Adding basalt of the 0.0/2.0 mm fraction group to the horizontal laboratory mixer's container (hopper)	Basalt, 0.0/2.0 mm fraction group	Horizontal laboratory mixer	1–2 min	
22.			4	Mixing crushed fine basalt aggregate of the 0.0/2.0 mm fraction group	Basalt, 0.0/2.0 mm fraction group	Horizontal laboratory mixer	1–2 min	
23.			5	Adding Portland cement to the horizontal laboratory mixer's container (hopper) CEM I 32.5R	Portland cement CEM I 32.5R	Horizontal laboratory mixer	1–2 min	
24.			6	Mixing Portland cement CEM I 32.5R	Portland cement CEM I 32.5R	Horizontal laboratory mixer	1–2 min	
25.			7	Mixing all dry ingredients in the hopper of the horizontal laboratory mixer	Granite, 2.0/8.0 mm fraction group	Horizontal laboratory mixer	4–6 min	
26.					Soda-lime glass granules, 0.0/2.0 mm fraction group			
27.					Portland cement CEM I 32.5R			
28.	4	Preparation of the concrete mix	1	Mixing the aqueous solution with admixture No. 1	Mixing water, tap water; pH = 7.0	Graduated laboratory beaker	12–16 min	16–24 min
29.						Calibrated analytical balance		
30.					Liquid Admixture No. 1—A highly plasticizing chemical admixture based on an aqueous solution of modified polycarboxylic ethers (1.50% by mass of cement binder)	Calibrated glass pipette		
31.						Magnetic stirrer		
36.			3	Adding tap water (mixing water) with a pH of 7.0 to the container (hopper) of the horizontal laboratory mixer	Mixing water, tap water; pH = 7.0	Horizontal laboratory mixer	1–2 min	

37.			4	Mixing all dry ingredients and tap water (mixing water) with a pH of 7.0 in the container (hopper) of the horizontal laboratory mixer	Mixing water, tap water; pH = 7.0	Horizontal laboratory mixer	1–2 min	
38.					Portland cement CEM I 32.5R			
39.					Soda-lime glass granules, 0.0/2.0 mm fraction group			
40.					Granite, 2.0/8.0 mm fraction group			
41.			5	Adding the aqueous solution with Admixture No. 1 to the container (hopper) of the horizontal laboratory mixer	Aqueous solution with liquid admixture No. 1—A highly plasticizing chemical admixture based on an aqueous solution of modified polycarboxylic ethers (1.50% by mass of cement binder)	Horizontal laboratory mixer	1–2 min	
42.								
44.								
45.			6	Mixing all dry and liquid components in the horizontal laboratory mixer's hopper (feed bin)	Mixing water, tap water; pH = 7.0	Horizontal laboratory mixer	1–2 min	
46.					Aqueous solution with liquid admixture No. 1—A highly plasticizing chemical admixture based on an aqueous solution of modified polycarboxylic ethers (1.50% by mass of cement binder)			
48.					Portland cement CEM I 32.5R			
49.					Granite, 2.0/8.0 mm fraction group			
50.					Granite, 2.0/8.0 mm fraction group			
51.	5	Preparation of concrete mix samples	1	Placing the concrete mix into molds	Mixing water, tap water; pH = 7.0	Plastic molds	10–15 min	10 min and 20 s to 15 min and 30 s
52.					Aqueous solution with liquid admixture No. 1—A highly plasticizing chemical admixture based on an aqueous solution of modified polycarboxylic ethers (1.50% by mass of cement binder)			

54.					Portland cement CEM I 32.5R	Trowel		
55.					Granite, 2.0/8.0 mm fraction group			
56.					Granite, 2.0/8.0 mm fraction group			
57.			2	Mechanical compaction of the concrete mix for layer No. 1	Mixing water, tap water; pH = 7.0	Vibrating table	10–15 s	
58.					Aqueous solution with liquid admixture No. 1—A highly plasticizing chemical admixture based on an aqueous solution of modified polycarboxylic ethers (1.50% by mass of cement binder)			
60.					Portland cement CEM I 32.5R	Steel float for finishing (standard "C" type cubic samples (100x100x100) mm)		
61.					Granite, 2.0/8.0 mm fraction group			
62.					Granite, 2.0/8.0 mm fraction group			
63.			3	Mechanical compaction of the concrete mix for layer No. 2	Mixing water, tap water; pH = 7.0	Vibrating table	10–15 s	
64.					Aqueous solution with liquid admixture No. 1—A highly plasticizing chemical admixture based on an aqueous solution of modified polycarboxylic ethers (1.50% by mass of cement binder)			
66.					Portland cement CEM I 32.5R	Steel trowel for finishing (standard type "B" cubic samples (150x150x150) mm, beam samples (150x150x600) mm)		
67.					Basalt, 0.0/2.0 mm fraction group			
68.					Granite, 2.0/8.0 mm fraction group			
69.		Preparation of concrete samples	1	Demolding of concrete samples	Concrete	Compressor	Depending on the number of molds	> 672 h (demolding and 28 days)
70.						Compressor instrumentation		
71.			2	Curing of concrete samples	Concrete	Calibrated climatic chamber or pallet box with tap water	672 h (28 days)	
72.						Calibrated electronic thermo-hygrometer		

2) MAIN EQUIPMENT AND ANCILLARY EQUIPMENT

Mechanical strength laboratory tests included the following main and auxiliary equipment:

- Calibrated Form+Test MEGA 6-3000-150 static testing machine, with a measuring range of (100–3000) kN, and a module for flexural tensile strength testing with a range of (3–150) kN (main equipment).
- Calibrated compression plates for the Form+Test MEGA 6-3000-150 static testing machine (auxiliary equipment).
- Calibrated K-015 Toropol thermostatic chamber (frost resistance chamber) used for freeze–thaw cycles (main equipment).
- Calibrated Limit double-sided electronic caliper with a depth gauge, offering a range of (0–200) mm and an elementary division of d = 0.01 mm (auxiliary equipment).
- Calibrated 90° double-arm square, Limit brand, with dimensions (200x130) mm (auxiliary equipment).
- Calibrated electronic thermo-hygrometer, type LB-523/SMA LAB-EL (auxiliary equipment).
- Calibrated RADWAG WLC 10/A2 non-automatic electronic balance with a measuring range of (0–10) kg (auxiliary equipment).
- Calibrated RADWAG mass standard with a nominal mass of 1.0 kg; F2 class standard, with an adjustment cavity in the cylinder (auxiliary equipment).
- Calibrated RADWAG mass standard with a nominal mass of 2.0 kg; F2 class standard, with an adjustment cavity in the cylinder (auxiliary equipment).
- Calibrated RADWAG mass standard with a nominal mass of 5.0 kg; F2 class standard, with an adjustment cavity in the cylinder (auxiliary equipment).
- Calibrated setting master for external dimensions with a nominal length of 100.00 mm (auxiliary equipment).
- Calibrated Insize feeler gauge with a measuring range of (0.03–1.00) mm; 32 calibrated measuring blades (auxiliary equipment).

Laboratory tests concerning thermal parameters involved the following main and auxiliary equipment, namely:

- Uncalibrated portable, non-stationary Isomet 2114 Logger with a surface probe (main equipment).
- Calibrated electronic thermo-hygrometer, type LB-523/SMA LAB-EL (auxiliary equipment).
- Calibrated RADWAG WLC 10/A2 non-automatic electronic balance with a measuring range of (0–10) kg (auxiliary equipment).
- Calibrated RADWAG mass standard with a nominal mass of 1.0 kg; F2 class standard, with an adjustment cavity in the cylinder (auxiliary equipment).
- A calibrated Limit double-sided electronic caliper with a depth gauge, offering a range of (0–200) mm and an elementary division of d = 0.01 mm (auxiliary equipment).
- Calibrated setting master for external dimensions with a nominal length of 100.00 mm (auxiliary equipment).

Laboratory tests, conducted according to the designed mix formulations, to image the macro-, meso-, and microstructure of concrete and cement pastes, utilized the following main and auxiliary equipment:

- Keyence VHX 7200 digital optical microscope with accessories (a set of lenses providing (20–2500)x optical magnification).

3) ENVIRONMENTAL CONDITIONS

Environmental conditions were established for the tests on mechanical strength, physical parameters, durability, and microscopic observation of concrete structure, as follows:
- Laboratory room: air temperature T = (22±2)°C and relative air humidity W = (54±2)%;
- Laboratory room—sample preparation stage: air temperature T = (22±2)°C and relative air humidity W = (58±2)%;
- Curing of concrete samples for 28 days: temperature of circulating tap water in the pallet box T_w = (18±2)°C;
- Freezing chamber (surface and internal frost resistance test): freezing air temperature T_f = (-18±2)°C and water temperature for thawing samples T_w = (18±2)°C.

Environmental conditions were established for the tests involving thermal parameter measurements, as follows:
- Laboratory room: air temperature T = (22±2)°C and relative air humidity W = (58±2)%.
- Laboratory room—sample preparation and testing stages: air temperature T = (21±1)°C and relative air humidity W = (56±2)%.
- Component temperatures: total mixing water T_w = (20±2)°C; general purpose cement CEM I 32,5R – Tc = (22±2)°C; liquid chemical admixture no. 1 (superplasticizer) – Td1=(20±2)°C.

4) CONCRETE MIX FORMULATIONS

The formulations for the reference mix (R) and the mixes materially modified with mineral wool from demolished heating system insulation (M1, M2, M3) were designed using B. Bukowski's method and the concept of hybrid aggregate mixtures, adhering to standard grading limit curves (standard grading limit curves up to 8.0 mm; D_{max} = 8.0 mm). Table 2 details the compositions of the reference (R) and material-modified (M1, M2, M3) formulations. This table, originally Table 1 in the main article, is included here as supplementary material for translation clarity.

Table S2. Tabular summary of the designed concrete mix compositions (including quantitative adjustments post-laboratory testing): reference formulation (R) and three material-modified formulations using a waste component (M1, M2, M3) (author's own work).

No.	Concrete Mix Component	Component Quantity in Concrete Mix [kg/m^3]			
		R	M1	M2	M3
1.	Mixing water, tap water; pH = 7.0	197	197	197	197
2.	Hydraulic Binder—General Purpose Cement CEM I 32.5R	405	405	405	405
3.	Liquid Admixture No. 1—A highly plasticizing chemical admixture based on an aqueous solution of modified polycarboxylic ethers (1.50% by mass of cement binder)	6.08	6.08	6.08	6.08
8.	Crushed fine aggregate—Basalt, 0.0/2.0 mm fraction group	936	936	936	936
9.	Crushed coarse aggregate—Granite, 2.0/8.0 mm fraction group	972	972	972	972
**10.**	**Waste mineral wool (12%, 16%, and 20% of the reference mass of the cement binder)**	**0**	**48.6**	**64.8**	**81.0**
11.	Air content in the concrete mixture	4.5	4.6	4.7	4.7
12.	Air content in concrete	5.0	5.2	5.4	5.4

A calculation example for the reference concrete mix formulation is presented below, utilizing B. Bukowski's method and standard grading limit curves for concrete mixes with a maximum aggregate size of D_{max} = 8.0 mm. The simplified calculation process is outlined in 10 steps, presented concisely without detailed calculations or component characteristics. The complex, comprehensive method for the qualitative and quantitative selection of concrete mix formulations has also been omitted.

1. Granulometric analysis of aggregates from the 0.0/8.0 mm aggregate mixture—a dry sieve analysis performed using a laboratory shaker with three representative analytical samples of 0.0/2.0 mm basalt fraction and 2.0/8.0 mm coarse crushed granite fraction (using a set of standard square-mesh sieves).

Basalt 0.0/2.0 mm—Pp1							
No.	Sieve mesh size # [mm]	Aggregate fraction [mm/mm]	Analytical sample No. 1 [g]	Analytical sample No. 2 [g]	Analytical sample No. 3 [g]	Average mass retained on sieve from three screenings [g]	Average mass retained on sieve from three screenings [%]
1.	63.0	63.0/125	0.00	0.00	0.00	0.00	0.00
2.	31.5	31.5/63.0	0.00	0.00	0.00	0.00	0.00
3.	16.0	16.0/31.5	0.00	0.00	0.00	0.00	0.00
4.	8.0	8.0/16.0	0.00	0.00	0.00	0.00	0.00
5.	4.0	4.0/8.0	0.00	0.00	0.00	0.00	0.00
6.	2.0	2.0/4.0	26.47	13.66	21.45	20.53	1.03
7.	1.0	1.0/2.0	745.76	715.66	752.44	737.95	**37.02**
8.	0.500	0.500/1.0	452.45	428.72	445.85	442.34	**22.19**
9.	0.250	0.250/0.500	367.82	374.59	347.73	363.38	**18.23**
10.	0.125	0.125/0.250	268.59	316.66	276.34	287.20	**14.41**
11.	0.063	0.063/0.125	116.45	121.45	126.34	121.41	**6.09**
12.	pan (0)	0/0.063	17.45	21.33	23.56	20.78	**1.04**
TOTAL :			**1994.99**	**1992.07**	**1993.71**	**1993.59**	**100.00**

1A. Sand point Pp1:	98.97	%
2A. Aggregate density Pp1, ρk1:	3	kg/dm^3

Granite 2.0/8.0 mm—Pp2							
No.	Sieve mesh size # [mm]	Aggregate fraction [mm/mm]	Analytical sample No. 1 [g]	Analytical sample No. 2 [g]	Analytical sample No. 3 [g]	Average mass retained on sieve from three screenings [g]	Average mass retained on sieve from three screenings [%]
1.	63.0	63.0/125	0.00	0.00	0.00	0.00	0.00
2.	31.5	31.5/63.0	0.00	0.00	0.00	0.00	0.00
3.	16.0	16.0/31.5	0.00	0.00	0.00	0.00	0.00
4.	8.0	8.0/16.0	13.66	9.44	15.67	12.92	0.65
5.	4.0	4.0/8.0	1189.54	1216.78	1218.44	1208.25	60.49
6.	2.0	2.0/4.0	725.45	713.49	719.45	719.46	36.02
7.	1.0	1.0/2.0	33.54	22.56	18.42	24.84	**1.24**
8.	0.500	0.500/1.0	16.43	14.59	17.55	16.19	**0.81**
9.	0.250	0.250/0.500	14.65	16.32	8.23	13.07	**0.65**

10.	0.125	0.125/0.250	3.45	4.37	0.00	2.61	**0.13**
11.	0.063	0.063/0.125	0.00	0.00	0.00	0.00	**0.00**
12.	pan (0)	0/0.063	0.00	0.00	0.00	0.00	**0.00**
TOTAL :			**1996.72**	**1997.55**	**1997.76**	**1997.34**	**100.00**

1B. Sand point Pp2:	2.84	%
2B. Aggregate density Pp2, ρk2:	2.65	kg/dm^3

2. Determination of auxiliary calculation parameter X1—using sand equivalent values Pp1 and Pp2 for crushed fine aggregate K1 and coarse aggregate K2. Assumption of the sand point (P) for a mixture of crushed fine aggregate K1 and coarse aggregate K2. Determination of the density for the aggregate mixture in a hybrid granular stack.

3. Assumed sand point of the aggregate mixture Pp (for the structural layer—single-layer material):	50	%
4. Auxiliary calculation parameter X1:	X1 = (Pp1 - Pp) / (Pp - Pp2) =	1.04
5. Proportion of fine aggregate relative to the total aggregate K1 / K:	K1 / K = 1 / (1 + X1) =	0.49
6. Proportion of coarse aggregate relative to the total aggregate K2 / K:	K2 / K = X1 / (1 + X1) =	**0.51**

3. Determination of the reference granular stack composed of a mixture of crushed aggregates K1 and K2. Percentage shares of individual fractions in the construction of the hybrid granular stack. Sieve analysis values as a function of sieve mesh size. Theoretical density of the aggregate mixture K1 and K2.

**Granular stack—a mixture of fine aggregate (soda-lime glass granulate 0.0/2.0 mm) and coarse aggregate (granite 2.0/8.0 mm)**									
No.	Sieve mesh size # [mm]	Aggregate fraction [mm/mm]	K1—fraction content [%]	K2—fraction content [%]	K—fraction content [%]	Screening [%]	Upper grading limit curve [%]	Lower grading limit curve [%]	Middle grading limit curve [%]
1.	63.0	63.0/125	0.00	0.00	0.00	**100.00**	100	100	100
2.	31.5	31.5/63.0	0.00	0.00	0.00	**100.00**	100	100	100
3.	16.0	16.0/31.5	0.00	0.00	0.00	**100.00**	100	100	100
4.	8.0	8.0/16.0	0.00	0.33	0.33	**99.67**	100	100	100
5.	4.0	4.0/8.0	0.00	30.82	30.82	**68.85**	85	61	75
6.	2.0	2.0/4.0	0.51	18.35	18.85	**50.00**	71	36	58
7.	1.0	1.0/2.0	18.16	0.63	18.79	**31.21**	57	21	40
8.	0.500	0.500/1.0	10.89	0.41	11.30	**19.91**	39	14	26
9.	0.250	0.250/0.500	8.94	0.33	9.28	**10.63**	21	5	11
10.	0.125	0.125/0.250	7.07	0.07	7.13	**3.50**	9	1	5

11.	0.063	0.063/0.125	2.99	0.00	2.99	**0.51**	5	0,5	2,5
12.	pan (0)	0/0.063	0.51	0.00	0.51	**0.00**	0	0	0
				TOTAL :	**100.00**				

7. Sand point of the aggregate blend Ppk:	50	%	
8. Theoretical density of aggregate K after mixing two aggregates ρk:	ρk = ρk1 * (K1 / K) + ρk2 * (K2 / K) =	2.82	kg/dm^3

4. Design of the grading curve for a hybrid aggregate mixture with a 0.0/8.0 mm particle size range (optimal grading zones). Examples of characteristic points on the designed grading curve—i.e., PP—fines point, PpP—fines-sand point, and Pp—sand point.

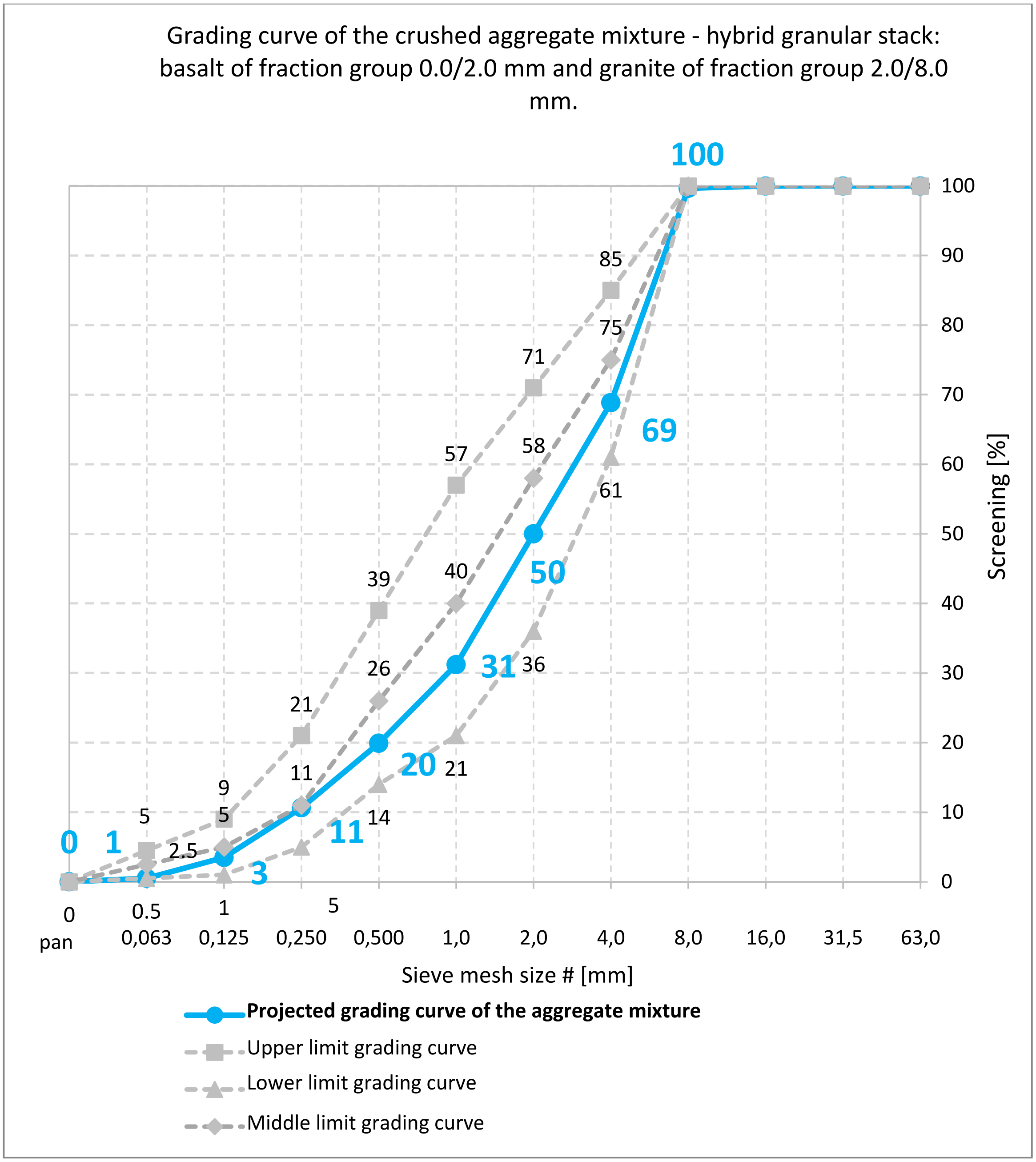

Grading curve of the crushed aggregate mixture - hybrid granular stack: basalt of fraction group 0.0/2.0 mm and granite of fraction group 2.0/8.0 mm.
Screening [%]
Sieve mesh size # [mm]
pan
0,063
0,125
0,250
0,500
1,0
2,0
4,0
8,0
16,0
31,5
63,0
Projected grading curve of the aggregate mixture
Upper limit grading curve
Lower limit grading curve
Middle limit grading curve

5. Calculation of water demand indices (concrete mix fluidity indicators) for 0.0/2.0 mm basalt aggregate. Fluidity indicators according to O. Stern's tables (moist consistency and mix consistency modified using two compatible liquid chemical admixtures).

**9. Aggregate water demand K1:**					
**Consistency V0—VeBe Method [s]—above 31 [s]: moist consistency V0**					
**Aggregate water demand K1—Basalt 0.0/2.0 mm**					
No.	Sieve mesh size # [mm]	Aggregate fraction [mm/mm]	Retained on sieve [%]	Water indices [dm^3/kg]—stiff-plastic consistency (Stern's table)	Product of aggregate retained on sieve and water indices (pi * wki)
1.	63.0	63.0/125	0.00	0.012	**0.00**
2.	31.5	31.5/63.0	0.00	0.012	**0.00**
3.	16.0	16.0/31.5	0.00	0.015	**0.00**
4.	8.0	8.0/16.0	0.00	0.018	**0.00**
5.	4.0	4.0/8.0	0.00	0.023	**0.00**
6.	2.0	2.0/4.0	1.03	0.029	**0.03**
7.	1.0	1.0/2.0	37.02	0.039	**1.44**
8.	0.500	0.500/1.0	22.19	0.053	**1.18**
9.	0.250	0.250/0.500	18.23	0.076	**1.39**
10.	0.125	0.125/0.250	14.41	0.109	**1.57**
11.	0.063	0.063/0.125	6.09	0.215	**1.31**
12.	pan (0)	0/0.063	1.04	0.215	**0.22**
TOTAL :			**100.00**	**0.82**	**7.14**

10. Water demand of aggregate K1 before correction:	$Wk01 = (1 / 100) * \sum (pi * wki) =$	0.071	[dm^3/kg]
11. Correction for aggregate K1 density:	$Wk1 = (2.65 / \rho k1) * Wk01 =$	0.063	[dm^3/kg]

6. Calculation of water demand indices (concrete mix workability indices) for crushed granite coarse aggregate, fraction group 2.0/8.0 mm. Fluidity indicators according to O. Stern's tables (moist consistency and mix consistency modified using two compatible liquid chemical admixtures).

**12. Water demand of aggregate K2:**					
**Consistency V0—VeBe Method [s]—above 31 [s]: moist consistency V0**					
**Water demand of K2 aggregate—Granite 2.0/8.0 mm**					
No.	Sieve mesh size # [mm]	Aggregate fraction [mm/mm]	Retained on sieve [%]	Water indices [dm^3/kg]—stiff-plastic consistency (Stern's table)	Product of aggregate retained on sieve and water indices (pi * wki)
1.	63.0	63.0/125	0.00	0,012	**0.00**
2.	31.5	31.5/63.0	0.00	0,012	**0.00**
3.	16.0	16.0/31.5	0.00	0,015	**0.00**
4.	8.0	8.0/16.0	0.65	0,018	**0.01**
5.	4.0	4.0/8.0	60.49	0,023	**1.39**
6.	2.0	2.0/4.0	36.02	0,029	**1.04**
7.	1.0	1.0/2.0	1.24	0,039	**0.05**
8.	0.500	0.500/1.0	0.81	0,053	**0.04**
9.	0.250	0.250/0.500	0.65	0,076	**0.05**
10.	0.125	0.125/0.250	0.13	0,109	**0.01**
11.	0.063	0.063/0.125	0.00	0,215	**0.00**
12.	pan (0)	0/0.063	0.00	0,215	**0.00**
TOTAL :			**100.00**	**0.82**	**2.60**

13. Water demand of K2 aggregate before correction:	Wk02 = (1 / 100) * ∑ (pi * wki) =	0.026	[dm^3/kg]
14. Correction based on aggregate K2 density:	Wk2 = (2.65 / ρk2) * Wk02 =	0.026	[dm^3/kg]
15. Water demand of K aggregate (K1 and K2):	Wk = Wk1 * (K1 / K) + Wk2 * (K2 / K) =	0.044	[dm^3/kg]

7. Varying the adopted environmental exposure classes for concrete. Accepted types of environmental hazards, namely: carbonation-induced aggression, chemical aggression, threat from non-marine chlorides, threat from freeze–thaw (F-T) cycles without de-icing salts, and carbonation corrosion (carbonation of the concrete structure). Selection of extreme values for exposure classes.

Parameter	Varying the adopted exposure classes (environmental aggression)				
	XF3	XM2	XA2	XD2	XC3

Maximum values of the water-cement ratio (W/C)	0.55	0.55	0.5	0.5	0.6
Minimum cement content (C)	320	300	320	320	280
Minimum concrete strength class (CX/Y)	C30/37	C30/37	C30/37	C30/37	C20/25

8. Design assumptions for the concrete mix. Adopting the minimum concrete strength class, determining the water-cement ratio (w/c) value, establishing standard limit values for mass of the cement binder, and defining the assumed consistency class of the concrete mix during the reference mix design phase.

**16. Design assumptions for the concrete mix formulation:**		
Exposure class:	XF3, XM2, XA2, XD2, XC3	-----
Minimum strength class:	C 30 / 37	-----
Water demand index – Maximum W/C:	0.50	-----
Minimum cement content:	320	[kg/m^3]
Minimum air content:	≥ 5.5%, or no aeration is required for V0 consistency and W/C ≤ 0.40	[%]
Consistency class:	Stiff plastic	-----
Strength class:	C 30 / 37	-----
Characteristic compressive strength f_{ck}:	37	[MPa]

9. Calculated and adopted parameters for concrete mix components—technical parameters of the cement binder. The water demand index and density of the cement binder, along with the percentage ratios of fine and coarse crushed aggregate to the total aggregate in the hybrid aggregate mixture.

**17. Cement properties:**		
Bulk density ρc:	3.16	[kg/dm^3]
Water demand of cement Wc:	0.28	reading from Stern's tables
Cement type and class:	CEM I 32.5R	

**17. Aggregates properties:**		
K1 / K:	0.49	-----
K2 / K:	0.51	-----
ρk1:	3	[kg/dm^3]
ρk2:	2.65	[kg/dm^3]
Pp1:	98.97	[%]

Pp2:	2.84	[%]
ρk:	2.82	[kg/dm^3]
Wk:	0.044	[dm^3/kg]

10. Calculation of the concrete mix formulation. Setting safety margins "x" and "y", adjusting strength based on the total air content percentage in the concrete mix and the hardened concrete, applying a strength correction with a reserve, and determining the "ω" coefficient as the inverse of the water-cement ratio (expressed as c/w). Calculating the mass of mix formulation components for 1.0 m^3 of concrete mix.

**19. Concrete mix formulation calculations:**				
Parameter "x"—strength safety margin:	"x" from 6 to 12		6	[MPa]
Strength correction due to "y" mix aeration (5.5% entrained air * 4 [MPa] strength drop for each percent of air):	4	5.5	22	[MPa]
Average compressive strength fcm:	$f_{cm} = f_{ck} + x + y =$		65	[MPa]

**20. Coefficient ω**:		
W/C ratio read from the graph showing the relationship between $f_{cm,cube}$ strength and W/C values (after 28 days of sample curing and for CEM 32.5 R):	0.49	-----
Coefficient ω: ω = 1 / (W/C) =	2.04	-----

**21. Calculating the concrete mix formulation components:**		
A. Aggregate—aggregate mixture (K1 + K2):		
K = 1000 / [(Wk / (1 - Wc * ω)) * (ω / ρc + 1) + (1 / ρk)] =	1908	[kg/m^3]
B. Mixing and tap water (W):		
W = [Wk / (1 - Wc * ω)] * K =	197	[kg/m^3]
C. CEM V cement (C):		
C = W * ω =	402	[kg/m^3]
D. Fine aggregate K1 (basalt, 0.0/2.0 mm):		
K1 = K * (K1 / K) =	936	[kg/m^3]
E. Coarse aggregate K2 (granite, 2.0/8.0 mm):		
K2 = K * (K2 / K) =	972	[kg/m^3]
Air volume (V):	45	[dm^3]
Bulk density of concrete mix (ρ):		
ρ_{bulk} = C + K1 + K2 + W =	2506	[kg/m^3]

5) ANALYSIS AND INTERPRETATION OF IN-HOUSE TEST RESULTS

Measurement values were quantitatively analyzed using descriptive and mathematical statistics. Descriptive statistics (statistical measures) were employed. These included mean values (trimmed, arithmetic, geometric, and harmonic), along with the Winsorized mean (used optionally for a minimum number of samples to eliminate the influence of extreme outliers). Also calculated were the standard errors of the measured quantity (shown as standard error bars), the medians (a measure of central tendency, or the second quartile, Q2), the sample and population standard deviations (measures of dispersion), and the standard errors dependent on the sample and population standard deviations.

Calculations were carried out for confidence values (-95% and 95%), the mode (modal values), extreme values (Min–Max), quantiles (lower Q1 and upper Q3 quartiles, and percentiles in the 10–90% range with 10% increments), variances (describing data variability within datasets), and coefficients of variation. The confidence interval values for standard deviations (-95% and 95%) were included, along with measures of variable distribution asymmetry (left- and right-skewness, standard error of skewness, and asymmetric distributions). Trend lines were developed for the final laboratory test results, indicating constant decrease, increase, or stability, and characterized by linearity.

Linear equations and R-squared (R^2) coefficients of determination were used to describe the regression's fit to the data. This provided a forecast of data change direction from the representative set, indicating the model's prediction level. Measurement uncertainty was estimated (using partial test reports) for a series of measured quantities, specifically: standard measurement uncertainty (u(x)), combined standard measurement uncertainty (uc(x)), and expanded measurement uncertainty ($U_p(x)$, based on the normal distribution cumulative distribution function); along with the expansion coefficient (k) and a confidence level (p) of 0.95 (95%) (a statistical method employing Type "A" measurement uncertainty).

- CHARACTERISTIC COMPRESSIVE STRENGTH ($f_{ck,cube}$) OF CONCRETE SAMPLES

Descriptive statistics for the characteristic compressive strength ($f_{ck,cube}$) were compiled and are presented in Table S3.

Table S3. Descriptive statistics for the measured characteristic compressive strength ($f_{ck,cube}$) values for samples from series R, M1, M2, and M3 (author's own work).

Variable	Descriptive Statistics—Characteristic Compressive Strength ($f_{ck,cube}$)				
	N Valid Samples	% Valid Samples	Mean	Confidence (-95.0%)	Confidence (95.0%)
26/LBW/4-(R):	3	100	84.9	78.7	91.1
26/LBW/4-(M1):	3	100	63.8	63.5	64.2
26/LBW/4-(M2):	3	100	66.4	63.9	68.9
26/LBW/4-(M3):	3	100	53.3	51.3	55.3

Variable	Trimmed Mean (5.0%)	Winsorized Mean (5.0%)	Geometric (Mean)	Harmonic (Mean)	Median
26/LBW/4-(R):	84.9	84.9	84.9	84.9	85.0
26/LBW/4-(M1):	63.8	63.8	63.8	63.8	63.8
26/LBW/4-(M2):	66.4	66.4	66.4	66.4	66.2

26/LBW/4-(M3):	53.3	53.3	53.3	53.3	53.4

Variable	Mode	Frequency (of Mode)	Minimum	Maximum	Lower (Quartile)
26/LBW/4-(R):	Multiple	1	82.4	87.4	82.4
26/LBW/4-(M1):	Multiple	1	63.7	64.0	63.7
26/LBW/4-(M2):	Multiple	1	65.5	67.5	65.5
26/LBW/4-(M3):	Multiple	1	52.4	54.0	52.4

Variable	Upper (Quartile)	Percentile (10.0)	Percentile (20.0)	Percentile (30.0)	Percentile (40.0)
26/LBW/4-(R):	87.4	82.4	82.4	82.4	85.0
26/LBW/4-(M1):	64.0	63.7	63.7	63.7	63.8
26/LBW/4-(M2):	67.5	65.5	65.5	65.5	66.2
26/LBW/4-(M3):	54.0	52.4	52.4	52.4	53.4

Variable	Percentile (50.0)	Percentile (60.0)	Percentile (70.0)	Percentile (80.0)	Percentile (90.0)
26/LBW/4-(R):	85.0	85.0	87.4	87.4	87.4
26/LBW/4-(M1):	63.8	63.8	64.0	64.0	64.0
26/LBW/4-(M2):	66.2	66.2	67.5	67.5	67.5
26/LBW/4-(M3):	53.4	53.4	54.0	54.0	54.0

Variable	Range	Quartile Range	Variance	Standard deviation	Standard deviation confidence interval (-95.000%)
26/LBW/4-(R):	5.0	5.0	6.3	2.5	1.3
26/LBW/4-(M1):	0.3	0.3	0.0	0.2	0.1
26/LBW/4-(M2):	2.0	2.0	1.0	1.0	0.5
26/LBW/4-(M3):	1.6	1.6	0.7	0.8	0.4

Variable	Standard deviation confidence interval (+95.000%)	Coefficient of variation	Standard error (Error)	Skewness	Standard error (Skewness)
26/LBW/4-(R):	15.7	2.9	1.4	-0.1	1.2
26/LBW/4-(M1):	1.0	0.2	0.1	0.9	1.2
26/LBW/4-(M2):	6.4	1.5	0.6	0.9	1.2
26/LBW/4-(M3):	5.1	1.5	0.5	-0.7	1.2

Table S3 presents descriptive statistics for the test results obtained from four sample series: R, M1, M2 and M3. For each series, three valid samples were analyzed, representing 100% data completeness for the study. The compilation included basic measures of location and dispersion, such as arithmetic mean, median, trimmed mean, Winsorized mean, minimum and maximum values, quartiles, percentiles, range, variance, standard deviation, coefficient of variation, standard error, and skewness. The highest mean value was obtained for the reference series R at 84.9 MPa. Lower values were recorded for the modified series: 63.8 MPa for series M1; 66.4 MPa for series M2; and 53.3 MPa for series M3. All series with added waste mineral wool fibers showed a lower characteristic compressive strength ($f_{ck,cube}$) than the reference sample R, with the most unfavourable result obtained for series M3.

Similar values for the mean, median, and robust means indicate an absence of significant outliers in the test results and a high level of consistency in the ($f_{ck,cube}$) results for the series.

Table S3, with its descriptive statistics, allowed for observations regarding the characteristic compressive strength ($f_{ck,cube}$) specifically:

- The final test results were influenced by three valid standard cubic samples for each series considered.
- The highest arithmetic mean characteristic compressive strength ($f_{ck,cube}$) of 84.9 MPa was obtained for series R samples (note significant variation in final results for other mean values).
- Mode values (the most frequently occurring values) were determined to be multiple, indicating that no identical measured values occurred (i.e., there was no single most frequent mode value for $f_{ck,cube}$).
- Median values (M_e) and arithmetic mean values for characteristic compressive strength ($f_{ck,cube}$) were numerically similar.
- A positive skewness value of (+0.9) for M1 and M2 series samples resulted in a right-skewed distribution (a positively skewed distribution of measured values).
- Negative skewness values of (-0.1) and (-0.7) for R and M3 series samples, respectively, resulted in a left-skewed distribution (most data below the mean value, a negatively skewed distribution of measured values).
- No symmetrical skewness distribution was observed (i.e., no uniform distribution of characteristic compressive strength ($f_{ck,cube}$) values).

- SPLITTING TENSILE STRENGTH (f_{ct}) OF CONCRETE SAMPLES—BRAZILIAN METHOD

Descriptive statistics for splitting tensile strength (f_{ct}) were compiled and are presented in Table S4.

Table S4. Descriptive statistics for measured splitting tensile strength (f_{ct}) values for series R, M1, M2, and M3 samples (author's own work).

Variable	Descriptive Statistics—Splitting Tensile Strength (f_{ct})				
	N Valid Samples	% Valid Samples	Mean	Confidence (-95.0%)	Confidence (95.0%)
26/LBW/4-(R):	3	100	3.5	2.7	4.3
26/LBW/4-(M1):	3	100	3.8	2.4	5.2
26/LBW/4-(M2):	3	100	3.9	3.2	4.6
26/LBW/4-(M3):	3	100	3.5	2.7	4.3

Variable	Trimmed Mean (5.0%)	Winsorized Mean (5.0%)	Geometric (Mean)	Harmonic (Mean)	Median
26/LBW/4-(R):	3.5	3.5	3.5	3.5	3.6
26/LBW/4-(M1):	3.8	3.8	3.8	3.7	3.8
26/LBW/4-(M2):	3.9	3.9	3.9	3.9	3.8
26/LBW/4-(M3):	3.5	3.5	3.5	3.5	3.6

Variable	Mode	Frequency (of Mode)	Minimum	Maximum	Lower (Quartile)
26/LBW/4-(R):	Multiple	1	3.2	3.9	3.2

26/LBW/4-(M1):	Multiple	1	3.3	4.4	3.3
26/LBW/4-(M2):	Multiple	1	3.8	4.3	3.8
26/LBW/4-(M3):	Multiple	1	3.2	3.8	3.2

Variable	Upper (Quartile)	Percentile (10.0)	Percentile (20.0)	Percentile (30.0)	Percentile (40.0)
26/LBW/4-(R):	3.9	3.2	3.2	3.2	3.6
26/LBW/4-(M1):	4.4	3.3	3.3	3.3	3.8
26/LBW/4-(M2):	4.3	3.8	3.8	3.8	3.8
26/LBW/4-(M3):	3.8	3.2	3.2	3.2	3.6

Variable	Percentile (50.0)	Percentile (60.0)	Percentile (70.0)	Percentile (80.0)	Percentile (90.0)
26/LBW/4-(R):	3.6	3.6	3.9	3.9	3.9
26/LBW/4-(M1):	3.8	3.8	4.4	4.4	4.4
26/LBW/4-(M2):	3.8	3.8	4.3	4.3	4.3
26/LBW/4-(M3):	3.6	3.6	3.8	3.8	3.8

Variable	Range	Quartile Range	Variance	Standard Deviation	Confidence interval for the standard deviation (-95.0%)
26/LBW/4-(R):	0.7	0.7	0.1	0.3	0.2
26/LBW/4-(M1):	1.1	1.1	0.3	0.6	0.3
26/LBW/4-(M2):	0.5	0.5	0.1	0.3	0.1
26/LBW/4-(M3):	0.6	0.6	0.1	0.3	0.2

Variable	Confidence interval for the standard deviation (+95.0%)	Coefficient of variation	Standard error (Error)	Skewness	Standard error (Skewness)
26/LBW/4-(R):	2.0	9.2	0.2	-0.2	1.2
26/LBW/4-(M1):	3.5	14.5	0.3	0.0	1.2
26/LBW/4-(M2):	1.7	7.0	0.2	1.7	1.2
26/LBW/4-(M3):	2.0	8.9	0.2	-1.3	1.2

Table S4 presents descriptive statistics for the test results of four sample series: R, M1, M2, and M3. For each series, three valid samples were analyzed, corresponding to 100% completeness of data. The compilation included basic measures of central tendency and dispersion, such as the arithmetic mean, median, trimmed mean, Winsorized mean, minimum, maximum, quartiles, percentiles, range, variance, standard deviation, coefficient of variation, standard error, and skewness. An analysis of the mean values indicates that the tested parameter (f_{ct}) reached approximately 3.5–4.0 MPa; series M1 and M2 achieved the highest values, while series R and M3 had similar, lower values, as shown in the table. The median for all series was 3.6 MPa, indicating a high concentration (clustering) of test results around the central value. Furthermore, the similar values of the mean, median, and robust means suggest an absence of distinct observations of outliers.

An assessment of the dispersion measures reveals that series M1 exhibited the highest variability, with a recorded range of 1.1, a variance of 0.3, a standard deviation of 0.6, and a coefficient of variation

equalling 14.5%. For the other series, these values were lower and more similar to each other, indicating greater homogeneity in the results. For series R, M2, and M3, the range was approximately 0.6, the variance about 0.1, and the standard deviation approximately 0.3. The confidence intervals for the mean were wide, measuring 2.7–4.3 for series R, 2.4–5.2 for M1, 3.2–4.8 for M2, and 2.7–4.3 for M3, which is due to the small sample size. It was found that series M2 exhibited the most favorable level of the tested parameter (f_{ct}) with moderate variability in results. In comparison, series M1, despite having a more favorable mean value than the reference series R, showed the greatest scatter of results.

A table of descriptive statistics was prepared concerning the final results of splitting tensile strength tests (f_{ct}). The statistical values were compiled based on individual readings from the measuring device. Table 4 presents descriptive statistics for samples from series 26/LBW/4-R, 26/LBW/4-M1, 26/LBW/4-M2, and 26/ LBW/4-M3. Based on the results presented in the table, the following was found:

- Descriptive statistics values were calculated based on three valid samples representative of the research method (100% of samples considered valid for the final test results).

- The highest arithmetic mean ($X_{śr}$) value was (3.9±0.2) MPa, and the lowest arithmetic mean ($X_{śr}$) value was (3.5±0.2) MPa.

- The trimmed mean (T_m) value was equal to the arithmetic mean ($X_{śr}$).

- For the Winsorized mean, there was no rejection of minimum and maximum outliers (extreme values) from the set of obtained strength values (f_{ct}), showing a high susceptibility to errors related to the extremes of the obtained indicator values (no replacement with the nearest values for the extreme minimum and maximum strength values).

- The middle value (central tendency index resistant to ranked extreme values) for an odd number of samples—median M_e (Mdn., quartile of order 1/2, second quartile of Q2): minimum value equal to M_e = 3.6 MPa (50.00% of ranked strength values (f_{ct}) located below and above the value of 3.6 MPa); maximum value M_e = 3.8 MPa (50.00% of ranked strength values (f_{ct}) located below and above the value of 3.8 MPa).

- The value of the dominant (D, modal value, mode), (modes of the final results) as multiple—no occurrence of the dominant observation of the strength value (f_{ct}), no value (f_{ct}) common in the group of final results (no dominant feature in the data set, mode count equal to 1).

- Coefficient of variation of data (CV)—the highest observed value of variation equal to CV = 14.5 for the samples of the 26/LBW/4-M1 series, indicating high variation and heterogeneity of the results of the strength values (f_{ct}) in relation to the other values of the data dispersion measure (CV) for the samples of the 26/LBW/4-R, 26/LBW/4-M2 and 26/LBW/4-M3 series).

- Asymmetry of distribution in the data sets, i.e., skewness of strength values (f_{ct})—the occurrence of negative skewness for samples of the 26/LBW/4-R and 26/LBW/4-M3 series (results with $A_s<0$, left-skewed distribution of test results) and positive skewness for samples of the 26/LBW/4-M2 series (results with $A_s>0$, right-skewed distribution of test results). A symmetrical distribution was observed for the strength values (f_{ct}) in the test results of series 26/LBW/4-M1, relative to a hypothetical normal distribution.

- BULK DENSITY (ρ) OF CONCRETE SAMPLES

Descriptive statistics for bulk density (ρ) were compiled and are presented in Table S5.

Table S5. Descriptive statistics for measured bulk density (ρ) values for samples from series R, M1, M2, and M3 (author's own work).

Variable	Descriptive Statistics—Bulk Density (ρ)				
	N Valid Samples	% Valid Samples	Mean	Confidence (-95.0%)	Confidence (95.0%)
26/LBW/4-(R):	3	100	2344	2263	2425
26/LBW/4-(M1):	3	100	2265	2148	2383
26/LBW/4-(M2):	3	100	2289	2188	2389
26/LBW/4-(M3):	3	100	2251	2123	2378

Variable	Trimmed Mean (5.0%)	Winsorized Mean (5.0%)	Geometric (Mean)	Harmonic (Mean)	Median
26/LBW/4-(R):	2344	2344	2344	2343	2361
26/LBW/4-(M1):	2265	2265	2265	2265	2270
26/LBW/4-(M2):	2289	2289	2288	2288	2309
26/LBW/4-(M3):	2251	2251	2250	2250	2256

Variable	Mode	Frequency (of Mode)	Minimum	Maximum	Lower (Quartile)
26/LBW/4-(R):	Multiple	1	2306	2364	2306
26/LBW/4-(M1):	Multiple	1	2216	2310	2216
26/LBW/4-(M2):	Multiple	1	2242	2315	2242
26/LBW/4-(M3):	Multiple	1	2197	2299	2197

Variable	Upper (Quartile)	Percentile (10.0)	Percentile (20.0)	Percentile (30.0)	Percentile (40.0)
26/LBW/4-(R):	2364	2306	2306	2306	2361
26/LBW/4-(M1):	2310	2216	2216	2216	2270
26/LBW/4-(M2):	2315	2242	2242	2242	2309
26/LBW/4-(M3):	2299	2197	2197	2197	2256

Variable	Percentile (50.0)	Percentile (60.0)	Percentile (70.0)	Percentile (80.0)	Percentile (90.0)
26/LBW/4-(R):	2361	2361	2364	2364	2364
26/LBW/4-(M1):	2270	2270	2310	2310	2310
26/LBW/4-(M2):	2309	2309	2315	2315	2315
26/LBW/4-(M3):	2256	2256	2299	2299	2299

Variable	Range	Quartile Range	Variance	Standard Deviation	Confidence interval for the standard deviation (-95.0%)
26/LBW/4-(R):	58	58	1066	33	17
26/LBW/4-(M1):	94	94	2225	47	25
26/LBW/4-(M2):	73	73	1642	41	21
26/LBW/4-(M3):	102	102	2622	51	27

Variable	Confidence interval for the standard deviation (+95.0%)	Coefficient of variation	Standard error (Error)	Skewness	Standard error (Skewness)
26/LBW/4-(R):	205	1	19	-2	1
26/LBW/4-(M1):	296	2	27	0	1
26/LBW/4-(M2):	255	2	23	-2	1
26/LBW/4-(M3):	322	2	30	0	1

This table presents descriptive statistics for the bulk density (ρ) of concrete samples from series R, M1, M2, and M3. Three samples were analyzed for each series, and data completeness reached 100%. The average bulk density for the reference series R was 2344 kg/m³, whereas the modified series yielded 2265 kg/m³ for series M1; 2289 kg/m³ for series M2; and 2251 kg/m³ for series M3. This indicates a decrease in the analyzed parameter compared to the reference series by 79 kg/m³, 55 kg/m³, and 93 kg/m³, respectively, which translates to reductions of approximately 3.37%, 2.35% and 3.97%. The measures of central tendency are similar, suggesting an absence of significant outliers. The variability of the results is low, as indicated by a standard deviation in the range of 33–51 kg/m³ and a coefficient of variation at a level of 1–2%. These results suggest that incorporating waste mineral fibers reduces the bulk density of concrete; the least adverse effect was observed for series M2, while the most adverse was for series M3.

A table of descriptive statistics was compiled for the final bulk density (ρ) test results. The statistical values were derived from individual readings obtained from the measuring device. Table 18 presents descriptive statistics for samples from series 26/LBW/4-R, 26/LBW/4-M1, 26/LBW/4-M2, and 26/LBW/4-M3. Based on the results presented in the table, the following was observed:

- Descriptive statistics values were calculated based on three valid samples representative of the research method (100% of samples considered valid for the final test results).

- The highest arithmetic mean ($X_{śr}$) was (2344±19) kg/m³, and the lowest arithmetic mean ($X_{śr}$) was (2251±30) kg/m³.

- The trimmed mean (T_m) value was equal to the arithmetic mean ($X_{śr}$).

- For the Winsorized mean, there was no rejection of minimum and maximum outliers (extreme values) from the set of obtained bulk density values (p), indicating a high susceptibility to errors related to the extremes of the obtained indicator values (no replacement with the nearest values for the extreme minimum and maximum bulk density values).

- The middle value (central tendency index resistant to ranked extreme values) for an odd number of samples—the median M_e (Mdn, quartile of order 1/2, second quartile of Q2): minimum value equal to M_e = 2256 kg/m³ (50.00% of ranked bulk density (ρ) values, located below and above the value of 2256 kg/m³); maximum value M_e = 2361 kg/m³ (50.00% of ranked bulk density (ρ) values, located below and above the value of 2361 kg/m³).

- The value of the dominant (D, modal value, mode), (modes of the final results) as multiple—no occurrence of a dominant observation of the value of bulk density (ρ), the absence of the value (ρ) common in the group of final results (no dominant feature in the data set, mode count equal to 1).

- Coefficient of variation of data (CV)—the highest observed value of variation equal to CV = 2 for the samples of the 26/LBW/4-M1, 26/LBW/4-M2 and 26/LBW/4-M3 series, indicating low variation and homogeneity of bulk density (ρ) value results.

- Asymmetry of distribution in the data sets, i.e., skewness of bulk density (ρ) values—the occurrence of negative skewness for the samples of the 26/LBW/4-R series and for 26/LBW/4-M2 (two results with $A_s<0$, left-skewed distribution of test results). A symmetrical distribution was observed for the final bulk density (ρ) test results of samples from series 26/LBW/4-M1 and 26/LBW/4-M3, relative to a hypothetical normal distribution.

- SURFACE WATER ABSORPTION (n_w) OF CONCRETE SAMPLES

Descriptive statistics for surface water absorption (n_w) were compiled and are presented in Table S6.

Table S6. Descriptive statistics for the measured surface water absorption (n_w) values of samples from series R, M1, M2, and M3 (author's own work).

Variable	Descriptive Statistics—Surface Water Absorption (n_w)				
	N Valid Samples	% Valid Samples	Mean	Confidence (-95.0%)	Confidence (95.0%)
26/LBW/4-(R):	3	100	6.0	5.7	6.3
26/LBW/4-(M1):	3	100	7.7	6.9	8.6
26/LBW/4-(M2):	3	100	7.9	7.2	8.5
26/LBW/4-(M3):	3	100	8.7	8.5	8.9

Variable	Trimmed Mean (5.0%)	Winsorized Mean (5.0%)	Geometric (Mean)	Harmonic (Mean)	Median
26/LBW/4-(R):	6.0	6.0	6.0	6.0	6.0
26/LBW/4-(M1):	7.7	7.7	7.7	7.7	7.9
26/LBW/4-(M2):	7.9	7.9	7.8	7.8	8.0
26/LBW/4-(M3):	8.7	8.7	8.7	8.7	8.7

Variable	Mode	Frequency (of Mode)	Minimum	Maximum	Lower (Quartile)
26/LBW/4-(R):	Multiple	1	5.9	6.1	5.9
26/LBW/4-(M1):	Multiple	1	7.4	8.0	7.4
26/LBW/4-(M2):	Multiple	1	7.5	8.0	7.5
26/LBW/4-(M3):	Multiple	1	8.6	8.7	8.6

Variable	Upper (Quartile)	Percentile (10.0)	Percentile (20.0)	Percentile (30.0)	Percentile (40.0)
26/LBW/4-(R):	6.1	5.9	5.9	5.9	6.0
26/LBW/4-(M1):	8.0	7.4	7.4	7.4	7.9
26/LBW/4-(M2):	8.0	7.5	7.5	7.5	8.0
26/LBW/4-(M3):	8.7	8.6	8.6	8.6	8.7

Variable	Percentile (50.0)	Percentile (60.0)	Percentile (70.0)	Percentile (80.0)	Percentile (90.0)
26/LBW/4-(R):	6.0	6.0	6.1	6.1	6.1
26/LBW/4-(M1):	7.9	7.9	8.0	8.0	8.0
26/LBW/4-(M2):	8.0	8.0	8.0	8.0	8.0
26/LBW/4-(M3):	8.7	8.7	8.7	8.7	8.7

Variable	Range	Quartile Range	Variance	Standard Deviation	Confidence interval for the standard deviation (-95.0%)
26/LBW/4-(R):	0.2	0.2	0.0	0.1	0.1
26/LBW/4-(M1):	0.6	0.6	0.1	0.3	0.2
26/LBW/4-(M2):	0.5	0.5	0.1	0.3	0.1
26/LBW/4-(M3):	0.1	0.1	0.0	0.1	0.0

Variable	Confidence interval for the standard deviation (+95.0%)	Coefficient of variation	Standard error (Error)	Skewness	Standard error (Skewness)
26/LBW/4-(R):	0.7	1.9	0.1	-1.0	1.2
26/LBW/4-(M1):	2.1	4.3	0.2	-1.5	1.2
26/LBW/4-(M2):	1.7	3.5	0.2	-1.7	1.2
26/LBW/4-(M3):	0.4	0.8	0.0	-0.8	1.2

Table S6 presents the descriptive statistics for surface water absorption (n_w) for sample series R, M1, M2, and M3. Three samples were analyzed for each series, and the data completeness was 100%. The reference series R had an average surface water absorption of 6.0%; the modified series, however, yielded 7.7% for M1, 7.9% for M2, and 8.7% for M3, respectively. An increase in the analyzed parameter (n_w) was observed relative to the reference sample by 1.8%, 1.9%, and 2.7%, respectively. Similar values for the mean, median, and robust means indicate good data consistency and the absence of significant outliers. Measures of dispersion, such as variance (0.0–0.1), standard deviation (0.1–0.3), and a low coefficient of variation, confirm the high repeatability of the results. The results indicate that using waste mineral wool fibers leads to increased surface water absorption in concrete, thereby deteriorating the material's impermeability and structural compactness. The statistical analysis conducted showed that all sample series had complete data, with three samples analyzed for each. The reference series R exhibited the lowest average surface water absorption at 6.0%. In contrast, all series modified with waste mineral wool fibers showed higher values: 7.7% for series M1, 7.9% for series M2, and 8.7% for series M3. The tested parameter (n_w) increased by 29.2%, 31.1%, and 44.9%, respectively, compared to the reference sample R. The consistency among the primary measures of central tendency suggests the absence of significant outliers, while low values for dispersion measures and the coefficient of variation confirm the high repeatability of the results.

A table of descriptive statistics was compiled for the final results of the surface water absorption (n_w) tests. The statistical values were derived from individual readings obtained from the measuring device. Table 6 presents the descriptive statistics for samples from series 26/LBW/4-R, 26/LBW/4-M1,

26/LBW/4-M2, and 26/LBW/4-M3. Based on the results presented in the table, the following was observed:

- Descriptive statistics values were calculated based on three valid samples representative of the research method (100% of samples considered valid for the final test results).

- The highest arithmetic mean ($X_{śr}$) was (8.7±0.1)%, and the lowest arithmetic mean ($X_{śr}$) was (6.0±0.1)%.

- The trimmed mean (T_m) value was equal to the arithmetic mean ($X_{śr}$).

- For the Winsorized mean, there was no rejection of minimum and maximum outliers (extreme values) from the set of obtained bulk density values (p), indicating a high susceptibility to errors related to the extremes of the obtained indicator values (no replacement with the nearest values for the extreme minimum and maximum surface water absorption values (n_w).

- The middle value (central tendency index resistant to ranked extreme values) for an odd number of samples—median M_e (Mdn., quartile of order 1/2, second quartile of Q2): minimum value equal to M_e = 6.0% (50.0% of ranked values of surface water absorption (n_w) located below and above the value of 6.0%); maximum value M_e = 8.7% (50.0% of ranked values of surface water absorption (n_w) located below and above the value of 8.7%).

- The value of the dominant (D, modal value, mode), (modes of the final results) as multiple—no occurrence of the dominant observation of the value of surface water absorption (n_w), no value (n_w) common in the group of final results (no dominant feature in the data set, mode count equal to 1).

- Coefficient of variation of data (CV)—the highest observed value of variation equal to CV = 4.3 for the samples of the 26/LBW/4-M1 series, indicating high variation and heterogeneity of the surface absorbability values (n_w) in relation to the other values of the data dispersion measure (CV) for the samples of the 26/LBW/4-R, 26/LBW/4-M2 and 26/LBW/4-M3 series).

- Asymmetry of distribution in the data sets, i.e., skewness of the surface water absorption values (n_w)—the occurrence of negative skewness for all series of samples (four results with $A_s<0$, left-skewed distribution of test results).

- EXTERNAL AND INTERNAL FROST RESISTANCE OF CONCRETE SAMPLES

Descriptive statistics for the characteristic compressive strength ($f_{ck,cube}$) before 100 freeze–thaw cycles (internal concrete frost resistance) are presented in Table S7.

Table S7. Descriptive statistics for the measured characteristic compressive strength ($f_{ck,cube}$) values of samples from series R, M1, M2, and M3 (internal concrete frost resistance test before 100 freeze–thaw cycles) (author's own work).

Variable	Descriptive Statistics—Internal Frost Resistance—Characteristic Compressive Strength ($f_{ck,cube}$) Before 100 F-T Cycles				
	N Valid Samples	% Valid Samples	Mean	Confidence (-95.0%)	Confidence (95.0%)
26/LBW/4-(R):	6	100.0	93.7	80.7	106.7
26/LBW/4-(M1):	6	100.0	76.0	73.8	78.3
26/LBW/4-(M2):	6	100.0	74.6	71.3	77.9
26/LBW/4-(M3):	6	100.0	59.9	55.4	64.3

Variable	Trimmed Mean (5.0%)	Winsorized Mean (5.0%)	Geometric (Mean)	Harmonic (Mean)	Median
26/LBW/4-(R):	93.7	93.7	93.0	92.2	100.6
26/LBW/4-(M1):	76.0	76.0	76.0	76.0	75.8
26/LBW/4-(M2):	74.6	74.6	74.6	74.5	74.7
26/LBW/4-(M3):	59.9	59.9	59.7	59.6	61.3

Variable	Mode	Frequency (of Mode)	Minimum	Maximum	Lower (Quartile)
26/LBW/4-(R):	Multiple	1	76.7	103.6	79.0
26/LBW/4-(M1):	Multiple	1	72.9	78.9	75.0
26/LBW/4-(M2):	Multiple	1	70.8	78.1	71.7
26/LBW/4-(M3):	Multiple	1	52.7	64.1	57.1

Variable	Upper (Quartile)	Percentile (10.0)	Percentile (20.0)	Percentile (30.0)	Percentile (40.0)
26/LBW/4-(R):	102.0	76.7	79.0	79.0	99.6
26/LBW/4-(M1):	77.8	72.9	75.0	75.0	75.6
26/LBW/4-(M2):	77.8	70.8	71.7	71.7	73.3
26/LBW/4-(M3):	62.8	52.7	57.1	57.1	61.0

Variable	Percentile (50.0)	Percentile (60.0)	Percentile (70.0)	Percentile (80.0)	Percentile (90.0)
26/LBW/4-(R):	100.6	101.5	102.0	102.0	103.6
26/LBW/4-(M1):	75.8	76.0	77.8	77.8	78.9
26/LBW/4-(M2):	74.7	76.1	77.8	77.8	78.1
26/LBW/4-(M3):	61.3	61.5	62.8	62.8	64.1

Variable	Range	Quartile Range	Variance	Standard Deviation	Confidence interval for the standard deviation (-95.0%)
26/LBW/4-(R):	26.9	23.0	153.5	12.4	7.7
26/LBW/4-(M1):	6.0	2.8	4.5	2.1	1.3
26/LBW/4-(M2):	7.3	6.1	9.9	3.1	2.0
26/LBW/4-(M3):	11.4	5.7	17.9	4.2	2.6

Variable	Confidence interval for the standard deviation (+95.0%)	Coefficient of variation	Standard error (Error)	Skewness	Standard error (Skewness)
26/LBW/4-(R):	30.4	13.2	5.1	-0.9	0.8
26/LBW/4-(M1):	5.2	2.8	0.9	-0.1	0.8
26/LBW/4-(M2):	7.7	4.2	1.3	-0.1	0.8
26/LBW/4-(M3):	10.4	7.1	1.7	-1.1	0.8

Variable	Kurtosis	Standard error (Kurtosis)
26/LBW/4-(R):	-1.8	1.7
26/LBW/4-(M1):	-0.2	1.7
26/LBW/4-(M2):	-2.3	1.7
26/LBW/4-(M3):	0.5	1.7

A table of descriptive statistics was compiled for the final results of characteristic compressive strength tests ($f_{ck,cube}$) on standard "C" type concrete samples (100x100x100) mm (internal concrete frost resistance test before 100 F-T cycles). Table 7 presents descriptive statistics for samples from series 26/LBW/4-R, 26/LBW/4-M1, 26/LBW/4-M2, and 26/LBW/4-M3. Based on the results presented in the table, the following was observed:

- Descriptive statistics values were calculated based on six valid samples representative of the research method (100% of samples considered valid for the final test results).

- The highest arithmetic mean ($X_{śr}$) was (93.7±5.1) MPa, while the lowest arithmetic mean ($X_{śr}$) was (59.9±1.7) MPa.

- The trimmed mean (T_m) value was equal to the arithmetic mean ($X_{śr}$).

- For the Winsorized mean, minimum and maximum outliers were rejected from the set of characteristic compressive strength ($f_{ck,cube}$) values obtained, showing low susceptibility to errors from extreme readings (by replacing the extreme minimum and maximum characteristic compressive strength ($f_{ck,cube}$) values with the nearest non-outlier values).

- The middle value (central tendency index resistant to ranked extreme values) for an odd number of samples—the median M_e (Mdn, quartile of order 1/2, second quartile of Q2): minimum value equal to M_e = 61.3 MPa (50.00% of the ranked values of the characteristic compressive strength ($f_{ck,cube}$) of the R-series samples located below and above the 61.3 MPa value); maximum value M_e = 100.6 MPa (50.00% of the ranked values of the characteristic compressive strength ($f_{ck,cube}$) of the M1-series samples located below and above the 100.6 MPa value).

- The value of the dominant (D, modal value, mode), (modes of the final results) as multiple—no occurrence of the dominant observation of the characteristic compressive strength value ($f_{ck,cube}$), no value ($f_{ck,cube}$) common in the group of final results (no dominant feature in the data set, mode count equal to 1).

- Coefficient of variation of data (CV)—the highest observed value of variation equal to CV = 13.2 for the samples of the 26/LBW/4-R series, which indicates high variation and heterogeneity of the results

of the characteristic compressive strength ($f_{ck,cube}$) in relation to the other values of the data dispersion measure (CV) for the samples of the 26/LBW/4-M1, 26/LBW/4-M2 and 26/LBW/4-M3 series).

- Asymmetry of distribution in the data sets, i.e., skewness of the characteristic compressive strength values ($f_{ck,cube}$)—the occurrence of negative skewness for samples of all sample series (results with $A_s<0$, left-skewed distribution of test results).

Descriptive statistics for the characteristic compressive strength after 100 F-T cycles (internal frost resistance of concrete) ($f_{ck,cube}$) were developed and are presented in Table S8.

Table S8: Descriptive statistics for the characteristic compressive strength after 100 F-T cycles (internal frost resistance of concrete) ($f_{ck,cube}$)

Variable	Descriptive Statistics—Internal Frost Resistance—Characteristic Compressive Strength ($f_{ck,cube}$) After 100 F-T Cycles				
	N Valid Samples	% Valid Samples	Mean	Confidence (-95.0%)	Confidence (95.0%)
26/LBW/4-(R):	6	100.0	93.2	86.0	100.3
26/LBW/4-(M1):	6	100.0	71.0	68.0	74.0
26/LBW/4-(M2):	6	100.0	70.6	66.8	74.4
26/LBW/4-(M3):	6	100.0	57.3	54.3	60.3

Variable	Trimmed Mean (5.0%)	Winsorized Mean (5.0%)	Geometric (Mean)	Harmonic (Mean)	Median
26/LBW/4-(R):	93.2	93.2	93.0	92.7	94.2
26/LBW/4-(M1):	71.0	71.0	71.0	70.9	70.7
26/LBW/4-(M2):	70.6	70.6	70.5	70.4	71.1
26/LBW/4-(M3):	57.3	57.3	57.2	57.2	56.4

Variable	Mode	Frequency (of Mode)	Minimum	Maximum	Lower (Quartile)
26/LBW/4-(R):	Multiple	1	84.0	103.0	87.4
26/LBW/4-(M1):	Multiple	1	67.1	74.9	69.4
26/LBW/4-(M2):	Multiple	1	64.5	74.5	68.8
26/LBW/4-(M3):	54.9	2	54.9	62.2	54.9

Variable	Upper (Quartile)	Percentile (10.0)	Percentile (20.0)	Percentile (30.0)	Percentile (40.0)
26/LBW/4-(R):	96.2	84.0	87.4	87.4	92.3
26/LBW/4-(M1):	73.3	67.1	69.4	69.4	69.8
26/LBW/4-(M2):	73.6	64.5	68.8	68.8	70.8
26/LBW/4-(M3):	59.1	54.9	54.9	54.9	56.2

Variable	Percentile (50.0)	Percentile (60.0)	Percentile (70.0)	Percentile (80.0)	Percentile (90.0)
26/LBW/4-(R):	94.2	96.1	96.2	96.2	103.0
26/LBW/4-(M1):	70.7	71.6	73.3	73.3	74.9
26/LBW/4-(M2):	71.1	71.4	73.6	73.6	74.5
26/LBW/4-(M3):	56.4	56.5	59.1	59.1	62.2

Variable	Range	Quartile Range	Variance	Standard Deviation	Confidence interval for the standard deviation (-95.0%)
26/LBW/4-(R):	19.0	8.8	46.5	6.8	4.3
26/LBW/4-(M1):	7.8	3.9	8.0	2.8	1.8
26/LBW/4-(M2):	10.0	4.8	13.1	3.6	2.3
26/LBW/4-(M3):	7.3	4.2	8.1	2.9	1.8

Variable	Confidence interval for the standard deviation (+95.0%)	Coefficient of variation	Standard error (Error)	Skewness	Standard error (Skewness)
26/LBW/4-(R):	16.7	7.3	2.8	0.0	0.8
26/LBW/4-(M1):	6.9	4.0	1.2	0.1	0.8
26/LBW/4-(M2):	8.9	5.1	1.5	-0.9	0.8
26/LBW/4-(M3):	7.0	5.0	1.2	1.2	0.8

Variable	Kurtosis	Standard error (Kurtosis)
26/LBW/4-(R):	-0.5	1.7
26/LBW/4-(M1):	-0.8	1.7
26/LBW/4-(M2):	0.8	1.7
26/LBW/4-(M3):	0.7	1.7

A table of descriptive statistics was compiled for the final results of characteristic compressive strength tests ($f_{ck,cube}$) on standard "C" type concrete samples (100x100x100) mm (internal concrete frost resistance test after 100 F-T cycles). Table 8 presents descriptive statistics for samples from series 26/LBW/4-R, 26/LBW/4-M1, 26/LBW/4-M2, and 26/LBW/4-M3. Based on the results presented in the table, the following was observed:

- Descriptive statistics values were calculated based on six valid samples representative of the research method (100% of samples considered valid for the final test results).

- The highest arithmetic mean ($X_{śr}$) was (93.2±2.8) MPa, and the lowest arithmetic mean ($X_{śr}$) was (57.3±1.2) MPa.

- The trimmed mean (T_m) value was equal to the arithmetic mean ($X_{śr}$).

- For the Winsorized mean, minimum and maximum outliers were rejected from the set of characteristic compressive strength ($f_{ck,cube}$) values obtained, showing low susceptibility to errors from extreme readings (by replacing the extreme minimum and maximum characteristic compressive strength ($f_{ck,cube}$) values with the nearest non-outlier values).

- The middle value (central tendency index resistant to ranked extreme values) for an odd number of samples—the median Me (Mdn, quartile of order 1/2, second quartile of Q2): minimum value equal to M_e = 56.4 MPa (50.00% of the ranked values of the characteristic compressive strength ($f_{ck,cube}$) of the

R-series samples located below and above the 56.4 MPa value); maximum value M_e = 94.2 MPa (50.00% of the ranked values of the characteristic compressive strength ($f_{ck,cube}$) of the M1-series samples located below and above the 94.2 MPa value).

- The value of the dominant (D, modal value, mode), (modes of the final results) as multiple and equal to 54.9 MPa—the occurrence of the dominant observation of the value of the characteristic compressive strength ($f_{ck,cube}$) for samples of the 26/LBW/4-M3 series, mode count equal to 1 and 2.

- Coefficient of variation of data (CV)—the highest observed value of variation equal to CV = 7.3 for the samples of the 26/LBW/4-R series, which indicates high variation and heterogeneity of the results of the characteristic compressive strength (fck,cube) in relation to the other values of the data dispersion measure (CV) for the samples of the 26/LBW/4-M1, 26/LBW/4-M2 and 26/LBW/4-M3 series).

- Asymmetry of the distribution in the data sets, i.e. skewness of the characteristic compressive strength value ($f_{ck,\ cube}$)—occurrence of positive skewness for the samples of series 26/LBW/4-R, 26/LBW/4-M1 and 26/LBW/4-M3 (results with a value of A_s>0, right-skewed distribution of test results).

Descriptive statistics were compiled for the changes in mass values of samples from series R, M1, M2, and M3 (an external frost resistance test of concrete before 100 F-T cycles), and these are presented in Table S9.

Table S9. Descriptive statistics for the measured mass change values of water-saturated samples from series R, M1, M2, and M3 (external frost resistance test of concrete before 100 F-T cycles) (author's own work).

Variable	Descriptive Statistics—External Frost Resistance—Mass Change in Water-Saturated State Before 100 F-T cycles				
	N Valid Samples	% Valid Samples	Mean	Confidence (-95.0%)	Confidence (95.0%)
26/LBW/4-(R):	6	100	2340	2302	2378
26/LBW/4-(M1):	6	100	2276	2254	2298
26/LBW/4-(M2):	6	100	2289	2255	2322
26/LBW/4-(M3):	6	100	2216	2172	2260

Variable	Trimmed Mean (5.0%)	Winsorized Mean (5.0%)	Geometric (Mean)	Harmonic (Mean)	Median
26/LBW/4-(R):	2340	2340	2340	2340	2339
26/LBW/4-(M1):	2276	2276	2276	2276	2268
26/LBW/4-(M2):	2289	2289	2289	2288	2288
26/LBW/4-(M3):	2216	2216	2216	2215	2227

Variable	Mode	Frequency (of Mode)	Minimum	Maximum	Lower (Quartile)
26/LBW/4-(R):	Multiple	1	2294	2385	2313
26/LBW/4-(M1):	Multiple	1	2260	2315	2263
26/LBW/4-(M2):	Multiple	1	2244	2332	2269
26/LBW/4-(M3):	Multiple	1	2156	2267	2175

Variable	Upper (Quartile)	Percentile (10.0)	Percentile (20.0)	Percentile (30.0)	Percentile (40.0)
26/LBW/4-(R):	2371	2294	2313	2313	2321
26/LBW/4-(M1):	2283	2260	2263	2263	2266
26/LBW/4-(M2):	2312	2244	2269	2269	2275
26/LBW/4-(M3):	2244	2156	2175	2175	2222

Variable	Percentile (50.0)	Percentile (60.0)	Percentile (70.0)	Percentile (80.0)	Percentile (90.0)
26/LBW/4-(R):	2339	2356	2371	2371	2385
26/LBW/4-(M1):	2268	2269	2283	2283	2315
26/LBW/4-(M2):	2288	2301	2312	2312	2332
26/LBW/4-(M3):	2227	2232	2244	2244	2267

Variable	Range	Quartile Range	Variance	Standard Deviation	Confidence interval for the standard deviation (-95.0%)
26/LBW/4-(R):	91	58	1290	36	22
26/LBW/4-(M1):	55	20	429	21	13
26/LBW/4-(M2):	88	43	1029	32	20
26/LBW/4-(M3):	111	69	1792	42	26

Variable	Confidence interval for the standard deviation (+95.0%)	Coefficient of variation	Standard error (Error)	Skewness	Standard error (Skewness)
26/LBW/4-(R):	88	2	15	0	1
26/LBW/4-(M1):	51	1	8	2	1
26/LBW/4-(M2):	79	1	13	0	1
26/LBW/4-(M3):	104	2	17	0	1

Variable	Kurtosis	Standard error (Kurtosis)
26/LBW/4-(R):	-1.9	1.7
26/LBW/4-(M1):	3.0	1.7
26/LBW/4-(M2):	-0.9	1.7
26/LBW/4-(M3):	-1.2	1.7

A table of descriptive statistics was prepared for the final test results of mass changes in water-saturated samples (standard "C" type concrete samples) (100x100x100) mm (internal frost resistance test of concrete before 100 F-T cycles). Table 9 presents descriptive statistics for samples from series 26/LBW/4-R, 26/LBW/4-M1, 26/LBW/4-M2, and 26/LBW/4-M3. Based on the results presented in the table, the following was observed:

- Descriptive statistics values were calculated based on six valid samples representative of the research method (100% of samples considered valid for the final test results).

- The highest arithmetic mean ($X_{śr}$) was (2340±15) g, and the lowest arithmetic mean ($X_{śr}$) was (2216±17) g.

- The trimmed mean (T_m) value was equal to the arithmetic mean ($X_{śr}$).

- For the Winsorized mean, minimum and maximum outliers were rejected from the set of obtained mass change values for water-saturated samples, showing low susceptibility to errors related to extreme measured values (values were replaced with the nearest ones for extreme minimum and maximum mass changes of water-saturated samples (m).

- The middle value (central tendency index resistant to ranked extreme values) for an odd number of samples—the median Me (Mdn, quartile of order 1/2, second quartile of Q2): minimum value equal to Me =2227 g (50.00% of the ranked values of changes in the mass of water-saturated samples (m) of samples of the M3 series located below and above the value of 2227 g); maximum value Me = 2339 g (50.00% of the ranked values of changes in the mass of water-saturated samples (m) for the R series located below and above the value of 2339 g).

- The value of the dominant (D, modal value, mode), (modes of the final results) as multiple—no occurrence of the dominant for the observation of the value of changes in the mass of water-saturated samples (m) for all series, mode count equal to 1.

- Coefficient of variation of data (CV)—the highest observed value of variation equal to CV = 2 for the samples of the 26/LBW/4-M1 series, indicating high variation and heterogeneity in the results of changes in the mass of water-saturated samples (m) in relation to the other values of the data dispersion measure (CV) for the samples of the 26/LBW/4-R, 26/LBW/4-M2 and 26/LBW/4-M3 series).

- Data distribution asymmetry, specifically the skewness of mass change values for water-saturated samples (m), showed positive skewness for samples from series 26/LBW/4-M1 (a single result with $A_s>0$, indicating a right-skewed distribution of test results); no skewness was observed for samples from series 26/LBW/4-R, 26/LBW/4-M2, and 26/LBW/4-M3 (three results with $A_s = 0$, indicating no skewness).

Descriptive statistics concerning mass value changes for samples from series R, M1, M2, and M3 (external frost resistance testing of concrete after 100 F-T cycles) have been compiled and are presented in Table S10.

Table S10. Descriptive statistics for the measured mass change values of water-saturated samples from series R, M1, M2, and M3 (external frost resistance test of concrete after 100 F-T cycles) (author's own work).

Variable	Descriptive Statistics—External Frost Resistance—Mass Change in Water-Saturated State After 100 F-T Cycles				
	N Valid Samples	% Valid Samples	Mean	Confidence (-95.0%)	Confidence (95.0%)
26/LBW/4-(R):	6	100	2336	2298	2373
26/LBW/4-(M1):	6	100	2272	2249	2295
26/LBW/4-(M2):	6	100	2284	2250	2317
26/LBW/4-(M3):	6	100	2210	2166	2254

Variable	Trimmed Mean (5.0%)	Winsorized Mean (5.0%)	Geometric (Mean)	Harmonic (Mean)	Median
26/LBW/4-(R):	2336	2336	2335	2335	2335
26/LBW/4-(M1):	2272	2272	2272	2272	2263
26/LBW/4-(M2):	2284	2284	2283	2283	2280
26/LBW/4-(M3):	2210	2210	2209	2209	2221

Variable	Mode	Frequency (of Mode)	Minimum	Maximum	Lower (Quartile)
26/LBW/4-(R):	Multiple	1	2289	2381	2307
26/LBW/4-(M1):	Multiple	1	2251	2309	2258
26/LBW/4-(M2):	Multiple	1	2240	2329	2264
26/LBW/4-(M3):	Multiple	1	2151	2261	2168

Variable	Upper (Quartile)	Percentile (10.0)	Percentile (20.0)	Percentile (30.0)	Percentile (40.0)
26/LBW/4-(R):	2366	2289	2307	2307	2318
26/LBW/4-(M1):	2289	2251	2258	2258	2261
26/LBW/4-(M2):	2308	2240	2264	2264	2273
26/LBW/4-(M3):	2237	2151	2168	2168	2216

Variable	Percentile (50.0)	Percentile (60.0)	Percentile (70.0)	Percentile (80.0)	Percentile (90.0)
26/LBW/4-(R):	2335	2352	2366	2366	2381
26/LBW/4-(M1):	2263	2264	2289	2289	2309
26/LBW/4-(M2):	2280	2287	2308	2308	2329
26/LBW/4-(M3):	2221	2226	2237	2237	2261

Variable	Range	Quartile Range	Variance	Standard Deviation	Confidence interval for the standard deviation (-95.0%)
26/LBW/4-(R):	92	59	1311	36	23
26/LBW/4-(M1):	58	31	496	22	14
26/LBW/4-(M2):	89	44	1013	32	20
26/LBW/4-(M3):	110	69	1773	42	26

Variable	Confidence interval for the standard deviation (+95.0%)	Coefficient of variation	Standard error (Error)	Skewness	Standard error (Skewness)
26/LBW/4-(R):	89	2	15	0	1
26/LBW/4-(M1):	55	1	9	1	1
26/LBW/4-(M2):	78	1	13	0	1
26/LBW/4-(M3):	103	2	17	0	1

Variable	Kurtosis	Standard error (Kurtosis)
26/LBW/4-(R):	-2	2
26/LBW/4-(M1):	0	2
26/LBW/4-(M2):	-1	2
26/LBW/4-(M3):	-1	2

A table of descriptive statistics was prepared for the final test results of mass changes in water-saturated samples (standard "C" type concrete samples) (100x100x100) mm (internal frost resistance test of concrete after 100 F-T cycles). Table 10 presents the descriptive statistics for samples from series 26/LBW/4-R, 26/LBW/4-M1, 26/LBW/4-M2, and 26/LBW/4-M3. Based on the results presented in the table, the following was observed:

- Descriptive statistics values were calculated based on six valid samples representative of the research method (100% of samples considered valid for the final test results).

- The highest arithmetic mean ($X_{śr}$) was (2336±15) g, and the lowest arithmetic mean ($X_{śr}$) was (2216±17) g.

- The trimmed mean (T_m) value was equal to the arithmetic mean ($X_{śr}$).

- For the Winsorized mean, minimum and maximum outliers were rejected from the set of obtained mass change values for water-saturated samples, showing low susceptibility to errors related to extreme measured values (values were replaced with the nearest ones for extreme minimum and maximum mass changes of water-saturated samples (m).

- The middle value (central tendency index resistant to ranked extreme values) for an odd number of samples—the median M_e (Mdn, quartile of order 1/2, second quartile of Q2): minimum value equal to M_e = 2221 g (50.00% of the ranked values of changes in the mass of water-saturated samples (m) of samples of the M3 series located below and above the value of 2221 g); maximum value M_e = 2335 g (50.00% of the ranked values of changes in the mass of water-saturated samples (m) for the R series located below and above the value of 2335 g).

- The value of the dominant (D, modal value, mode), (modes of the final results) as multiple—no occurrence of the dominant for the observation of the value of changes in the mass of water-saturated samples (m) for all series, mode count equal to 1.

- Coefficient of variation of data (CV)—the highest observed value of variation equal to CV = 2 for the samples of the 26/LBW/4-R and 26/LBW/4-M3 series, indicating high variation and heterogeneity in the results of changes in the mass of water-saturated samples (m) in relation to the other values of the data dispersion measure (CV) for the samples of the 26/LBW/4-M1 and 26/LBW/4-M2 series).

- Distribution asymmetry in data sets, specifically the skewness of mass change values for water-saturated samples (m): positive skewness was observed for samples from series 26/LBW/4-M1 (a single result with A_s>0, indicating a right-skewed distribution of test results), while no skewness was observed for samples from series 26/LBW/4-R, 26/LBW/4-M2, and 26/LBW/4-M3 (three results with A_s = 0, indicating no skewness).

- THERMAL PARAMETERS

Descriptive statistics for the thermal conductivity coefficient (λ) test results are presented in Table S11.

Table S11. Descriptive statistics for the measured thermal conductivity coefficient (λ) values for samples from series R, M1, M2, and M3 (author's own work).

Variable	Descriptive Statistics—Thermal Parameter—Thermal Conductivity Coefficient (λ)				
	N Valid Samples	% Valid Samples	Mean	Confidence (-95.0%)	Confidence (95.0%)
26/LBW/4-(R):	10	100.0	1.3461	1.3411	1.3511
26/LBW/4-(M1):	10	100.0	1.1988	1.1968	1.2008
26/LBW/4-(M2):	10	100.0	1.2549	1.2524	1.2574
26/LBW/4-(M3):	10	100.0	1.2376	1.2348	1.2404

Variable	Trimmed Mean (5.0%)	Winsorized Mean (5.0%)	Geometric (Mean)	Harmonic (Mean)	Median
26/LBW/4-(R):	1.3462	1.3462	1.3461	1.3460	1.3444
26/LBW/4-(M1):	1.1985	1.1986	1.1988	1.1988	1.1978
26/LBW/4-(M2):	1.2555	1.2555	1.2549	1.2549	1.2557
26/LBW/4-(M3):	1.2376	1.2376	1.2376	1.2376	1.2366

Variable	Mode	Frequency (of Mode)	Minimum	Maximum	Lower (Quartile)
26/LBW/4-(R):	Multiple	1	1.3375	1.3541	1.3392
26/LBW/4-(M1):	1.1978	2	1.1955	1.2041	1.1968
26/LBW/4-(M2):	Multiple	1	1.2462	1.2591	1.2536
26/LBW/4-(M3):	Multiple	1	1.2320	1.2434	1.2339

Variable	Upper (Quartile)	Percentile (10.0)	Percentile (20.0)	Percentile (30.0)	Percentile (40.0)
26/LBW/4-(R):	1.3539	1.3382	1.3391	1.3402	1.3428
26/LBW/4-(M1):	1.2012	1.1959	1.1965	1.1970	1.1975
26/LBW/4-(M2):	1.2566	1.2498	1.2535	1.2540	1.2550
26/LBW/4-(M3):	1.2409	1.2329	1.2338	1.2350	1.2361

Variable	Percentile (50.0)	Percentile (60.0)	Percentile (70.0)	Percentile (80.0)	Percentile (90.0)
26/LBW/4-(R):	1.3444	1.3489	1.3536	1.3540	1.3541
26/LBW/4-(M1):	1.1978	1.1988	1.2005	1.2014	1.2029
26/LBW/4-(M2):	1.2557	1.2560	1.2565	1.2573	1.2586
26/LBW/4-(M3):	1.2366	1.2388	1.2408	1.2416	1.2428

Variable	Range	Quartile Range	Variance	Standard Deviation	Confidence interval for the standard deviation (-95.0%)
26/LBW/4-(R):	0.0166	0.0147	0.0000	0.0070	0.0048
26/LBW/4-(M1):	0.0086	0.0044	0.0000	0.0028	0.0019
26/LBW/4-(M2):	0.0129	0.0030	0.0000	0.0035	0.0024
26/LBW/4-(M3):	0.0114	0.0070	0.0000	0.0039	0.0027

Variable	Confidence interval for the standard deviation (+95.0%)	Coefficient of variation	Standard error (Error)	Skewness	Standard error (Skewness)
26/LBW/4-(R):	0.0128	0.5210	0.0022	0.1664	0.6870
26/LBW/4-(M1):	0.0050	0.2305	0.0009	0.7890	0.6870
26/LBW/4-(M2):	0.0065	0.2825	0.0011	-1.7096	0.6870
26/LBW/4-(M3):	0.0072	0.3185	0.0012	0.1518	0.6870

Variable	Kurtosis	Standard error (Kurtosis)
26/LBW/4-(R):	-2.0446	1.3342
26/LBW/4-(M1):	-0.3027	1.3342
26/LBW/4-(M2):	4.1331	1.3342
26/LBW/4-(M3):	-1.4511	1.3342

A table of descriptive statistics was prepared for the final test results of the thermal conductivity coefficient (λ). Table S11 presents descriptive statistics for samples from series 26/LBW/4-R, 26/LBW/M1-R, 26/LBWM2-R, and 26/LBW/4-M3. Based on the results presented in the table, the following was observed:

- Descriptive statistics values were calculated based on 10 valid samples representative of the research method (100% of samples considered valid for the final test results).

- The maximum arithmetic mean ($X_{śr}$) was (1.3411±0,0022)W/m·K, and the minimum arithmetic mean ($X_{śr}$) was (1.1988±0,0009)W/m·K.

- The trimmed mean (T_m) differed from the arithmetic mean ($X_{śr}$).

- For the Winsorized mean, there was no rejection of minimum and maximum outlier (extreme) values from the obtained thermal conductivity coefficient (λ) dataset, showing a high susceptibility to errors associated with extreme observed values (no replacement of extreme minimum and maximum thermal conductivity coefficient (λ) values with their nearest neighbours).

- The middle value (central tendency index resistant to ranked extreme values) for an odd number of samples—the median M_e (Mdn, quartile of order 1/2, second quartile of Q2): minimum value equal to M_e = (1.1978±0.0009)W/m·K (50.00% of the ranked values of thermal conductivity (λ) located below and above the value of (1.1978±0.0009) W/m·K); maximum value M_e = (1.3461±0.0022)W/m·K

(50.00% of the ranked values of thermal conductivity (λ) located below and above the value of 1.3461±0.0022)W/m·K).

- The value of the dominant (D, modal value, mode), (modes of the final results) as multiple and equal to 1.1985 W/m·K) for the samples of the series for 26/LBW/7-M1—dominant occurrence of the observation of the value of thermal conductivity (λ), no value of thermal conductivity (λ) common in the group of final results (no dominant characteristic in the data set, mode count equal to 1 and 2).

- Coefficient of variation of data (CV)—the highest observed value of variation equal to CV = 0.5210 for the samples of the 26/LBW/4-R series, indicating high variation and heterogeneity in the results of the thermal conductivity (λ) values in relation to the other values of the data dispersion measure (CV) for the other sample series.

- Asymmetry of distribution in the data sets, i.e., skewness of the thermal conductivity coefficient (λ) values—the occurrence of positive skewness for samples of the 26/LBW/7-R, 26/LBW/7-M1 and 26/LBW/7-M3 series (single result with $A_s>0$, right-skewed distribution of test results) and negative skewness for samples of the 26/LBW/4-M2 series (single result with $A_s<0$, left-skewed distribution of test results).

Descriptive statistics for the material's specific heat (c_p) test results have been compiled and are presented in Table S12.

Table S12. Descriptive statistics for the measured specific heat (c_p) values of material for R, M1, M2, and M3 series samples (author's own work).

Variable	Descriptive Statistics—Thermal Parameter—Specific Heat (c_p)				
	N Valid Samples	% Valid Samples	Mean	Confidence (-95.0%)	Confidence (95.0%)
26/LBW/4-(R):	10	100.0	1.7751	1.7702	1.7800
26/LBW/4-(M1):	10	100.0	1.6823	1.6798	1.6848
26/LBW/4-(M2):	10	100.0	1.7072	1.7034	1.7111
26/LBW/4-(M3):	10	100.0	1.6618	1.6584	1.6651

Variable	Trimmed Mean (5.0%)	Winsorized Mean (5.0%)	Geometric (Mean)	Harmonic (Mean)	Median
26/LBW/4-(R):	1.7750	1.7753	1.7751	1.7751	1.7755
26/LBW/4-(M1):	1.6826	1.6827	1.6823	1.6823	1.6819
26/LBW/4-(M2):	1.7069	1.7072	1.7072	1.7072	1.7057
26/LBW/4-(M3):	1.6616	1.6618	1.6618	1.6618	1.6612

Variable	Mode	Frequency (of Mode)	Minimum	Maximum	Lower (Quartile)
26/LBW/4-(R):	Multiple	1	1,7651	1,7857	1,7695
26/LBW/4-(M1):	Multiple	1	1,6752	1,6872	1,6805
26/LBW/4-(M2):	Multiple	1	1,7009	1,7161	1,7028
26/LBW/4-(M3):	Multiple	1	1,6560	1,6689	1,6575

Variable	Upper (Quartile)	Percentile (10.0)	Percentile (20.0)	Percentile (30.0)	Percentile (40.0)
26/LBW/4-(R):	1.7790	1.7668	1.7690	1.7699	1.7723
26/LBW/4-(M1):	1.6859	1.6777	1.6803	1.6811	1.6818
26/LBW/4-(M2):	1.7121	1.7016	1.7025	1.7033	1.7045
26/LBW/4-(M3):	1.6656	1.6563	1.6571	1.6585	1.6600

Variable	Percentile (50.0)	Percentile (60.0)	Percentile (70.0)	Percentile (80.0)	Percentile (90.0)
26/LBW/4-(R):	1.7755	1.7773	1.7784	1.7816	1.7850
26/LBW/4-(M1):	1.6819	1.6824	1.6844	1.6860	1.6867
26/LBW/4-(M2):	1.7057	1.7074	1.7104	1.7133	1.7153
26/LBW/4-(M3):	1.6612	1.6625	1.6644	1.6669	1.6686

Variable	Range	Quartile Range	Variance	Standard Deviation	Confidence interval for the standard deviation (-95.0%)
26/LBW/4-(R):	0.0206	0.0095	0.0000	0.0068	0.0047
26/LBW/4-(M1):	0.0120	0.0054	0.0000	0.0035	0.0024
26/LBW/4-(M2):	0.0152	0.0093	0.0000	0.0054	0.0037
26/LBW/4-(M3):	0.0129	0.0081	0.0000	0.0046	0.0032

Variable	Confidence interval for the standard deviation (+95.0%)	Coefficient of variation	Standard error (Error)	Skewness	Standard error (Skewness)
26/LBW/4-(R):	0.0125	0.3843	0.0022	0.1929	0.6870
26/LBW/4-(M1):	0.0064	0.2088	0.0011	-0.5302	0.6870
26/LBW/4-(M2):	0.0098	0.3141	0.0017	0.6046	0.6870
26/LBW/4-(M3):	0.0085	0.2795	0.0015	0.3665	0.6870

Variable	Kurtosis	Standard error (Kurtosis)
26/LBW/4-(R):	-0.9967	1.3342
26/LBW/4-(M1):	0.7491	1.3342
26/LBW/4-(M2):	-1.1112	1.3342
26/LBW/4-(M3):	-1.1958	1.3342

A table of descriptive statistics was compiled for the final test results of the material's specific heat (c_p). Table S12 presents descriptive statistics for samples from series 26/LBW/4-R, 26/LBW/M1-R, 26/LBWM2-R, and 26/LBW/4-M3. Based on the results presented in the table, the following was observed:

- Descriptive statistics values were calculated based on 10 valid samples representative of the research method (100% of samples considered valid for the final test results).

- The maximum arithmetic mean ($X_{śr}$) was (1.7751±0.0022)·10^6 J/m^3·K, and the minimum arithmetic mean ($X_{śr}$) was (1.6618±0.0015)·10^6 J/m^3·K.

- The trimmed mean (T_m) differed from the arithmetic mean ($X_{śr}$).

- For the Winsorized mean, there was no rejection of minimum and maximum outliers (extreme values) from the set of obtained specific heat values (c_p) of the material, indicating a high susceptibility to errors related to the extremes of the obtained indicator values (no replacement with the nearest values for the extreme minimum and maximum specific heat values (c_p) of the material.

- The middle value (central tendency index resistant to ranked extreme values) for an odd number of samples—the median Me (Mdn, quartile of order 1/2, second quartile of Q2): minimum value equal to Me =1.6612-10^6 J/m^3·K (50.00% of the ranked values of material specific heat (cp) located below and above the value of 1.6612-10^6 J/m3·K); maximum value M_e = 1.7755-10^6 J/m^3·K (50.00% of the ranked values of material specific heat (c_p) located below and above the value of 1.7755-10^6 J/m^3·K.

- The value of the dominant (D, modal value, mode), (modes of the final results) as multiple for all series of samples—no occurrence of the dominant observation of the material specific heat (c_p) value, no material specific heat (c_p) value common in the group of final results (no dominant feature in the data set, mode count equal to 1).

- Coefficient of variation of data (CV)—the highest observed value of variation equal to CV = 0.3843 for the samples of the 26/LBW/4-R series, indicating high variation and heterogeneity of the results of the material specific heat (c_p) values in relation to the other values of the data dispersion measure (CV) for the other sample series.

- Asymmetry of distribution in the data sets, i.e., skewness of the material's specific heat (c_p) value—the occurrence of positive skewness for samples of the 26/LBW/7-R, 26/LBW/7-M2 and 26/LBW/7-M3 series (three results with A_s>0, right-skewed distribution of test results) and negative skewness for sample of the 26/LBW/4-M1 series (single result with A_s<0, left-skewed distribution of test results).

Descriptive statistics for the thermal diffusivity (a) material test results were compiled and are presented in Table S13.

Table S13. Descriptive statistics for the measured thermal diffusivity (a) values observed in samples from series R, M1, M2, and M3 (author's own work).

Variable	Descriptive Statistics—Thermal Parameter—Thermal Diffusivity (a)				
	N Valid Samples	% Valid Samples	Mean	Confidence (-95.0%)	Confidence (95.0%)
26/LBW/4-(R):	10	100	0.7583	0.7542	0.7625
26/LBW/4-(M1):	10	100	0.7126	0.7109	0.7143
26/LBW/4-(M2):	10	100	0.7351	0.7328	0.7373
26/LBW/4-(M3):	10	100	0.7448	0.7426	0.7469

Variable	Trimmed Mean (5.0%)	Winsorized Mean (5.0%)	Geometric (Mean)	Harmonic (Mean)	Median
26/LBW/4-(R):	0.7582	0.7581	0.7583	0.7583	0.7590
26/LBW/4-(M1):	0.7125	0.7127	0.7126	0.7126	0.7123
26/LBW/4-(M2):	0.7350	0.7348	0.7350	0.7350	0.7364
26/LBW/4-(M3):	0.7446	0.7446	0.7447	0.7447	0.7443

Variable	Mode	Frequency (of Mode)	Minimum	Maximum	Lower (Quartile)
26/LBW/4-(R):	Multiple	1	0.7506	0.7671	0.7518
26/LBW/4-(M1):	Multiple	1	0.7090	0.7165	0.7109
26/LBW/4-(M2):	0.7369	2	0.7305	0.7402	0.7318
26/LBW/4-(M3):	0.7443	2	0.7407	0.7502	0.7419

Variable	Upper (Quartile)	Percentile (10.0)	Percentile (20.0)	Percentile (30.0)	Percentile (40.0)
26/LBW/4-(R):	0.7617	0.7509	0.7515	0.7542	0.7572
26/LBW/4-(M1):	0.7138	0.7097	0.7107	0.7114	0.7120
26/LBW/4-(M2):	0.7369	0.7308	0.7315	0.7326	0.7349
26/LBW/4-(M3):	0.7470	0.7411	0.7417	0.7429	0.7441

Variable	Percentile (50.0)	Percentile (60.0)	Percentile (70.0)	Percentile (80.0)	Percentile (90.0)
26/LBW/4-(R):	0.7590	0.7609	0.7617	0.7633	0.7660
26/LBW/4-(M1):	0.7123	0.7125	0.7133	0.7150	0.7163
26/LBW/4-(M2):	0.7364	0.7367	0.7369	0.7370	0.7386
26/LBW/4-(M3):	0.7443	0.7453	0.7467	0.7473	0.7489

Variable	Range	Quartile Range	Variance	Standard Deviation	Confidence interval for the standard deviation (-95.0%)
26/LBW/4-(R):	0.0165	0.0099	0.0000	0.0058	0.0040
26/LBW/4-(M1):	0.0075	0.0029	0.0000	0.0024	0.0016
26/LBW/4-(M2):	0.0097	0.0051	0.0000	0.0032	0.0022
26/LBW/4-(M3):	0.0095	0.0051	0.0000	0.0030	0.0021

Variable	Confidence interval for the standard deviation (+95.0%)	Coefficient of variation	Standard error (Error)	Skewness	Standard error (Skewness)
26/LBW/4-(R):	0.0106	0.7644	0.0018	-0.0638	0.6870
26/LBW/4-(M1):	0.0043	0.3324	0.0007	0.4773	0.6870
26/LBW/4-(M2):	0.0058	0.4306	0.0010	-0.1471	0.6870
26/LBW/4-(M3):	0.0055	0.4062	0.0010	0.3610	0.6870

Variable	Kurtosis	Standard error (Kurtosis)
26/LBW/4-(R):	-1.2360	1.3342
26/LBW/4-(M1):	-0.2541	1.3342
26/LBW/4-(M2):	-0.9931	1.3342
26/LBW/4-(M3):	-0.5811	1.3342

A table of descriptive statistics was compiled for the final test results of the material's thermal diffusivity (a). Table S13 presents descriptive statistics for samples from series 26/LBW/4-R, 26/LBW/M1-R, 26/LBWM2-R, and 26/LBW/4-M3. Based on the results presented in the table, the following was observed:

- Descriptive statistics values were calculated based on 10 valid samples representative of the research method (100% of samples considered valid for the final test results).

- The maximum arithmetic mean ($X_{śr}$) was $(0.7583 \pm 0.0018) \cdot 10^{-6}$ m^2/s, and the minimum arithmetic mean ($X_{śr}$) was $(0.7126 \pm 0.0007) \cdot 10^{-6}$ m^2/s.

- The trimmed mean (T_m) differed from the arithmetic mean ($X_{śr}$).

- For the Winsorized mean, there was no rejection of minimum and maximum outliers (extreme values) from the set of obtained thermal diffusivity values (a) of the material, indicating a high susceptibility to errors related to the extremes of the obtained indicator values (no replacement with the nearest values for the extreme minimum and maximum thermal diffusivity values (a) of the material.

- The middle value (central tendency index resistant to ranked extreme values) for an odd number of samples—the median M_e (Mdn, quartile of order 1/2, second quartile of Q2): minimum value equal to $M_e = 0.7123\text{-}10^{-6}$ m^2/s (50.00% of ranked values of thermal diffusivity of material (a) located below and above values of $0.7123\text{-}10^{-6}$ m^2/s); maximum value $M_e = 0.7590\text{-}10^{-6}$ m^2/s (50.00% of ranked values of thermal diffusivity of material (a) located below and above values of $0.7590\text{-}10^{-6}$ m^2/s).

- The value of the dominant (D, modal value, mode), (modes of the final results) as multiple for samples of the 26/LBW/4-R and 26/LBW/4-M1 series—no occurrence of the dominant observation of the thermal diffusivity value of the material (a), no thermal diffusivity value of the material (a) common in the group of final results, the dominant equal to $0.7369\text{-}10^{-6}$ m^2/s and $0.7433\text{-}10^{-6}$ m^2/s for samples of the 26/LBW/4-M2 and 26/LBW/4-M3 series, respectively.

- Coefficient of variation of data (CV)—the highest observed value of variation equal to CV = 0.7644 for the samples of the 26/LBW/4-R series, indicating high variation and heterogeneity of the results of the thermal diffusivity (a) values in relation to the other values of the data dispersion measure (CV) for the other sample series.

- Asymmetry of distribution in the data sets, i.e., skewness of the material's thermal diffusivity value (a) —the occurrence of positive skewness for samples of the 26/LBW/7-M1 and 26/LBW/7-M3 series (two results with $A_s>0$, right-skewed distribution of test results) and negative skewness for samples of the 26/LBW/4-R and 26/LBW/4-M3 series (two results with $A_s<0$, left-skewed distribution of test results).
